# A Map of Recent Positive Selection in the Human Genome

**DOI:** 10.1371/journal.pbio.0040072

**Published:** 2006-03-07

**Authors:** Benjamin F Voight, Sridhar Kudaravalli, Xiaoquan Wen, Jonathan K Pritchard

**Affiliations:** **1**Department of Human Genetics, University of Chicago, Chicago, Illinois, United States of America; University of BathUnited Kingdom

## Abstract

The identification of signals of very recent positive selection provides information about the adaptation of modern humans to local conditions. We report here on a genome-wide scan for signals of very recent positive selection in favor of variants that have not yet reached fixation. We describe a new analytical method for scanning single nucleotide polymorphism (SNP) data for signals of recent selection, and apply this to data from the International HapMap Project. In all three continental groups we find widespread signals of recent positive selection. Most signals are region-specific, though a significant excess are shared across groups. Contrary to some earlier low resolution studies that suggested a paucity of recent selection in sub-Saharan Africans, we find that by some measures our strongest signals of selection are from the Yoruba population. Finally, since these signals indicate the existence of genetic variants that have substantially different fitnesses, they must indicate loci that are the source of significant phenotypic variation. Though the relevant phenotypes are generally not known, such loci should be of particular interest in mapping studies of complex traits. For this purpose we have developed a set of SNPs that can be used to tag the strongest ∼250 signals of recent selection in each population.

## Abstract 2

Applying their newly developed method, the authors search International HapMap Project data representing three populations for signals of recent selection across the human genome.

## Introduction

The evolution of modern human populations has been accompanied by dramatic changes in environment and lifestyle. In the last 100,000 years, behaviorally modern humans have spread from Africa to colonize most of the globe. In that time, humans have been forced to adapt to a wide range of new habitats and climates. Following the end of the last ice age, 14,000 years ago, there was a major warming event that raised global temperatures to roughly their current levels. Further dramatic changes occurred with the transition from hunter-gatherer to agricultural societies, starting about 10,000–12,000 years ago in the Fertile Crescent, and a little later elsewhere. This was also a period marked by rapid increases in human population densities. Increased population density promoted the spread of infectious diseases, as did the new proximity of farmers to animal pathogens [1,2].

Each of these kinds of changes likely resulted in powerful selective pressures for new genotypes that were better suited to the novel environments. Indeed, there are a number of recent reports of genes that show signals of very strong and recent selection in favor of new alleles: for example, in response to malaria [3–5]; at the lactase gene in response to dairy farming [6]; at a salt sensitivity variant in response to climate [7]; and in genes involved in brain development [8,9].

To date, the best examples of recent selection in humans have all been discovered in studies of candidate genes where there was a prior hypothesis of selection. Hence, very little is known about how widespread such signals are; nor is there unbiased information about what kinds of genes or biological processes are most involved in the adaptation of modern humans. It is unclear whether these genes are the same kinds of genes that were most important in the earlier evolution of the *Homo* lineage, as identified from comparisons with chimpanzees [10–12]. It is also not known to what extent recent selective events have been geographically restricted, as opposed to taking place in all populations. A number of recent studies have detected more signals of adaptation in non-African populations than in Africans [13–17], and some of those studies have conjectured that non-Africans might have experienced greater pressures to adapt to new environments than Africans have.

In this study, we use newly available, dense, single nucleotide polymorphism (SNP) data from the International HapMap Project [18] to create a first-generation map of selection across the human genome. Our search is aimed at finding loci where there is strong, very recent, selection in favor of alleles that have not yet reached fixation. By doing so, we aim to provide preliminary answers to these questions about the nature and extent of recent adaptation in modern humans. The loci that we identify will start to fill in the details about the ways in which modern humans have adapted to the selective pressures in the most recent stage of our evolution.

Furthermore, signals of selective sweeps in progress indicate the presence of genetic variants that must have some significant effect on human phenotypic variation. Though the actual target and the nature of the selection are usually not immediately clear, it may well be that many of these variants affect complex phenotypes of medical relevance [19,7,20–22]. For this reason, we believe that a “selection map” of ongoing sweeps in the human genome will become an important aspect of annotation for the human genome sequence. Genome-wide association studies should be sure to tag haplotypes that appear to be targets of selection, and such haplotypes will be of particular interest if they produce association signals in mapping studies. To this end, we have created files of tag-SNPs that can be used to tag the major selection signals in each HapMap population, and a web tool, Haplotter, at http://pritch.bsd.uchicago.edu/data.html, that can be used to query any HapMap SNP for evidence of selection.

## Results

We analyzed genome-wide SNP data from Phase 1 of the International HapMap Project [18]. These data consist of ∼800,000 polymorphic SNPs in a total of 309 unrelated individuals. For the purpose of our analyses, we grouped the data into three distinct population samples of unrelated individuals, as follows (see Materials and Methods): 89 Japanese and Han Chinese individuals from Tokyo and Beijing, respectively, henceforth denoted as East Asian (ASN), 60 individuals of northern and western European origin (CEU), and 60 Yoruba (YRI) from Ibadan, Nigeria. Except where stated, our analyses focused on the autosomes only. Our analysis was based on haplotypes estimated by the HapMap Consortium using the program Phase 2 [18,23]. We estimated genome-wide, high-resolution LD-based recombination maps separately for all 3 samples, using our implementation of the Li and Stephens algorithm [24].

The goal in our study is to identify loci where strong selection has driven new alleles up to intermediate frequency. Such alleles might be on their way to fixation, or might become balanced polymorphisms. The classic signal of strong directional selection is that because the favored allele increases in frequency very fast, it tends to sit on an unusually long haplotype of low diversity. Meanwhile, chromosomes that do not carry the selected allele have levels of diversity and LD that are more typical of the genome as a whole. This type of signal has been used in the past to argue for selection in a number of genes in humans and in *Drosophila* [3,5,6,8,9,25–29].

**Figure 1:**
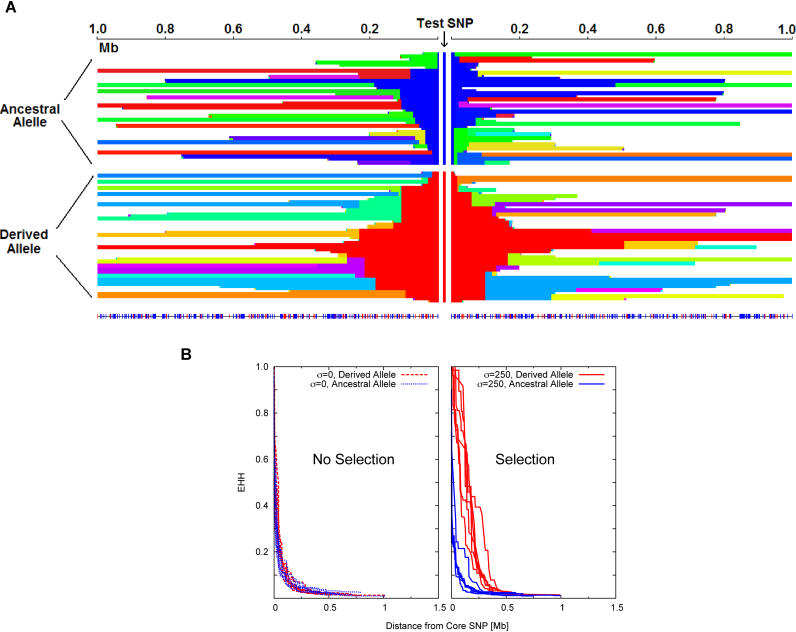
Decay of EHH in Simulated Data for an Allele at Frequency 0.5 (A) Decay of haplotypes in a single region in which a new selected allele (red, center column) is sweeping to fixation, replacing the ancestral allele (blue). Horizontal lines are haplotypes; SNP positions are marked below the haplotype plot using blue for SNPs with intermediate allele frequencies (minor allele >0.2), and red otherwise. For a given SNP, adjacent haplotypes with the same color carry identical genotypes everywhere between that SNP and the central (selected) site. The left- and right-hand sides are sorted separately. Haplotypes are no longer plotted beyond the points at which they become unique. (B) Decay of haplotype homozygosity for ten replicate simulations. When the core SNP is neutral (*s* = 0; left side) the haplotype homozygosity decays at similar rates for both ancestral and derived alleles. When the derived alleles are favored (*s* = 2*Ns* = 250; right side), the haplotype homozygosity decays much slower for the derived alleles than for the ancestral alleles. The discrepancy in the overall areas spanned by these two curves forms the basis of our text for selection (iHS).

Since our data consist of pre-ascertained SNPs, they do not directly contain information about the underlying levels of nucleotide diversity. Nonetheless, we can expect that favored alleles will generally sit within large shared haplotypes, and that these haplotypes will be in sharp contrast with the more variable haplotypes on the unselected background (Figure 1A). In order to pursue this type of signal for genome-wide SNP data, we have developed a new test statistic that we denote iHS (integrated haplotype score). The iHS was chosen after performing extensive simulations to determine the most powerful statistic from a number of new and previously published test statistics (see below and Figure S1).

Our new test begins with the EHH (extended haplotype homozygosity) statistic proposed by Sabeti et al. [5]. The EHH measures the decay of identity, as a function of distance, of haplotypes that carry a specified “core” allele at one end. For each allele, haplotype homozygosity starts at 1, and decays to 0 with increasing distance from the core site (Figure 1B). As shown in the figure, when an allele rises rapidly in frequency due to strong selection, it tends to have high levels of haplotype homozygosity extending much further than expected under a neutral model. Hence, in plots of EHH versus distance, the area under the EHH curve will usually be much greater for a selected allele than for a neutral allele. In order to capture this effect, we compute the integral of the observed decay of EHH away from a specified core allele until EHH reaches 0.05. This *integrated* EHH (iHH) (summed over both directions away from the core SNP) will be denoted iHH*_A_* or iHH*_D_*, depending on whether it is computed with respect to the ancestral or derived core allele. Finally, we obtain our test statistic iHS using



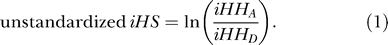



When the rate of EHH decay is similar on the ancestral and derived alleles, iHH*_A_*/iHH*_D_* ≍ 1, and hence the unstandardized iHS is ≍ 0. Large negative values indicate unusually long haplotypes carrying the derived allele; large positive values indicate long haplotypes carrying the ancestral allele. Since in neutral models, low frequency alleles are generally younger and are associated with longer haplotypes than higher frequency alleles, we adjust the unstandardized iHS to obtain our final statistic which has mean 0 and variance 1 regardless of allele frequency at the core SNP:



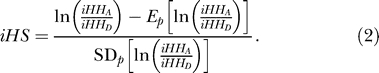



The expectation and standard deviation of ln(iHH*_A_*/iHH*_D_*) are estimated from the empirical distribution at SNPs whose derived allele frequency *p* matches the frequency at the core SNP. The iHS is constructed to have an approximately standard normal distribution and hence the sizes of iHS signals from different SNPs are directly comparable regardless of the allele frequencies at those SNPs. Since iHS is standardized using the genome-wide empirical distributions, it provides a measure of how *unusual* the haplotypes around a given SNP are, relative to the genome as a whole, and it does not provide a formal significance test.

In our data analysis, iHS is computed for every SNP with minor allele frequency > 5%, treating each SNP in turn as a core SNP. The iHS at each SNP measures the strength of evidence for selection acting at or near that SNP. However in simulations we have found that instead of treating each SNP separately, it is more powerful to look for windows of consecutive SNPs that contain *numerous* extreme iHS scores (Figure S2). This is because selective sweeps tend to produce clusters of extreme iHS scores across the sweep region, while under a neutral model, extreme iHS scores are scattered more uniformly (unpublished data).

In principle, we might expect that large negative iHS scores, indicating that a derived allele has swept up in frequency, are of the most interest. However, in simulations, a sweep can also produce large positive iHS values at nearby SNPs if ancestral alleles hitchhike with the selected site. Furthermore, it is plausible that selection may sometimes switch to favor an ancestral allele that has been segregating in the population. For these reasons, we will treat both extreme positive, and extreme negative iHS scores as potentially interesting.

It is now well-known that recombination rates are extremely heterogeneous across the genome, even at fine scales [30–32]. Such rate variation is a potential source of false positives when looking for regions with unusual haplotype structure as we are here. Our test is designed to control for rate variation in two ways. First we estimated high-resolution genetic maps based on LD patterns and used the estimated genetic distances when calculating iHS. By basing analysis on the fine scale genetic maps, the lengths of haplotypes that extend across large recombination coldspots are appropriately downweighted, and haplotypes that cross hotspots are upweighted. Second, since iHS is based on a ratio of haplotype homozygosities, the two alleles serve as internal controls for each other [5]. Hence, inaccuracies in the estimated genetic map will tend to cancel out of the ratio, as will any other factors that cause the extent of haplotype homozygosity to be heterogeneous across the genome.

**Figure 2:**
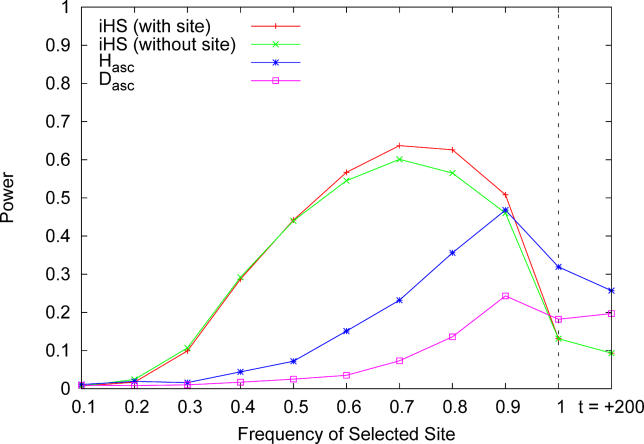
Power to Detect Sweeps-in-Progress at a *p*-Value of 0.01, Using Various Statistics Simulation parameters are matched to the Yoruba data, with *s* = 150. Tests are abased on 51-SNP windows centered on a selected site. The upper curves (iHS) are based on counting the number of SNPs in the window for which |iHS| >2. The green line indicates power when the actual SNP under selection is excluded from the analysis. The lower lines plot power using Fay and Wu's *H*, and Tajima's *D*, both calculated using the ascertained genotype data. The line marked *t* = +200 indicates the power 200 generations after fixation (*N_e_* = 10^4^). Critical values for each statistic at *p* = 0.01 were obtained using identical simulations with *s* = 0.

Figure 2 plots the power of iHS and of two standard tests of selection based on summaries of the frequency spectrum. These simulations, as well as the simulations shown below, are designed to match the properties of the data as closely as possible (Materials and Methods). As shown, the iHS outperforms the frequency spectrum tests, as well as other EHH-based statistics, across a broad range of frequencies of the selective sweep (Figure 2, Figure S2). Furthermore, iHS is robust to regional variation in SNP ascertainment while tests of the frequency spectrum may not be. Nonetheless, iHS has limited ability in the HapMap data to detect low frequency sweeps and to detect sweeps that are very near fixation.

The iHS statistic is constructed to provide a tool for identifying SNPs, or genomic regions, that are unusual relative to the genome as a whole, and not to provide formal significance testing relative to a theoretical model. We will show that in all populations there is an excess of extreme iHS signals relative to simulated models. However, since there is considerable uncertainty in simulated models, we prefer not to assign formal *p*-values to the signals that we find.

### Widespread Signals of Recent Selection

We calculated iHS for all SNPs with minor allele frequency >5%. Figure 3 shows a summary of the extreme values on Chromosome 2, with similar plots across all autosomes and the X chromosome provided in Figures S3–S25. The plotted points are those with |iHS| > 2.5, and correspond to the most extreme 1% of iHS values.

**Figure 3:**
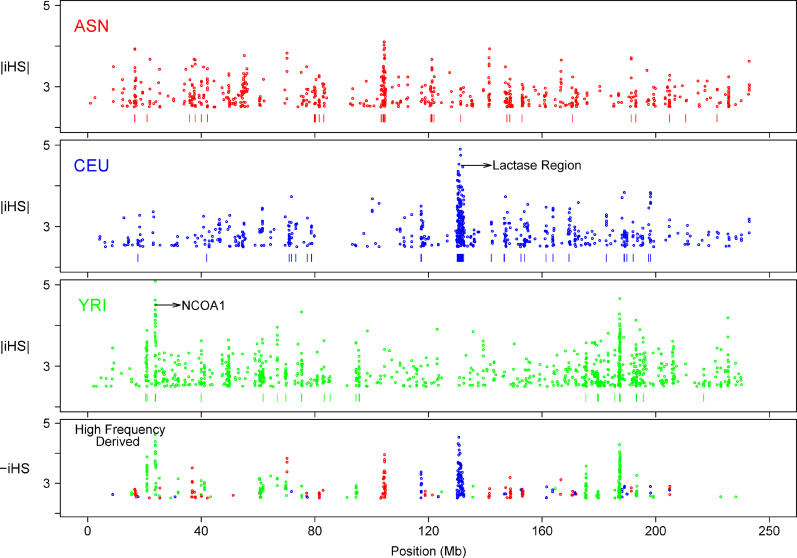
Plots of Chromosome 2 SNPs with Extreme iHS Values Indicate Discrete Clusters of Signals SNPs with |iHS| >2.5 (top 1%) are plotted. The bottom plot combines signals for all three populations, plotting only SNPs with derived frequency >0.5 and iHS <−2.5. Such SNPs correspond to high-frequency-derived SNPs in the range for which our test is most powerful. The short vertical bars below each plot indicate 100-kb windows whose signals are in the top 1% of windows genome-wide.

There is clear clustering of extreme values into distinct regions where many SNPs show evidence for selection. One such region, at 135–138 Mb in Europeans, contains the lactase gene *(LCT),* previously noted as a target of strong selection [6,33]. In principle, clustering of unusual iHS scores might occur even under a completely neutral model. However, several lines of evidence indicate that selection is indeed producing widespread signals in the data.

**Figure 4:**
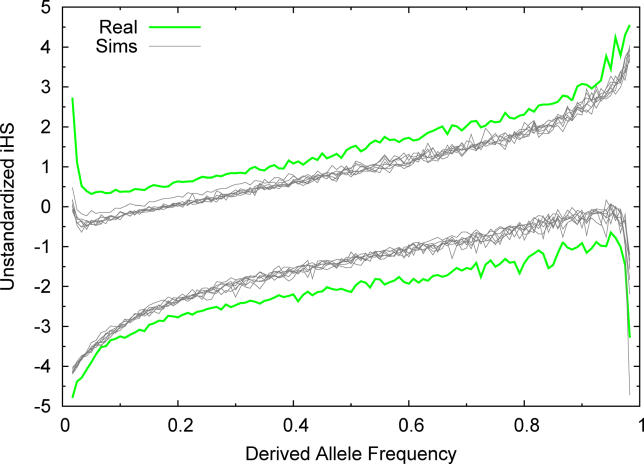
Central 99% Range of Unstandardized iHS for SNPs in the Yoruba Data and for SNPs in Matched Neutral Simulations The upper and lower lines mark the boundaries of the central 99% distribution of the unstandardized iHS ratio, as a function of derived allele frequency. The gray lines plot results for a range of plausible demographic models. The fatter tails in the real data are consistent with the action of selection.

First, simulations show that we observe more extreme values of the unstandardized iHS scores than expected under a range of neutral models (Figure 4). For each population, we performed neutral simulations that matched the observed SNP density and allele frequency spectrum, which included extensive recombination rate variation, including hotspots, and utilized a range of demographic models consistent with previous studies of demographic parameters (see Materials and Methods). The demographic models included a variety of bottleneck models for East Asians and Europeans, and models of constant size with recent growth for Yoruba [34,35]. Since previous genetic studies indicate that the Yoruba are likely to have the least complex demographic history [34–36], we focus mainly on simulation results for that population. We find that extreme values of the unstandardized iHS are more frequent in the real Yoruba data than in *any* of the simulated models, as expected if the largest iHS values are frequently due to selection. There is also an excess of extreme values in the Europeans, but in the East Asians some demographic models show as much variance as the real data (unpublished data).

Second, in all three populations, there is greater clustering of extreme (standardized) iHS values in the real data than in neutral simulations with heterogeneous recombination rates. For example, in simulations matching aspects of the Yoruba data, only 0.1% of windows of 50 consecutive SNPs had more than 16 SNPs with |iHS| > 20. In the actual YRI data, we observed a 14-fold enrichment of such windows. Since this calculation is based on empirically standardized scores, the signal of extra clustering is distinct from the previous signal of overdispersion of the unstandardized scores. Simulations designed to match the East Asian and European datasets also indicate that there is excess clustering of extreme iHS values in the real data, though for these populations the relative enrichment is quantitatively smaller due to the extra variance seen in neutral bottleneck models (the data show 2.3-fold and 2.7-fold enrichment of the top 0.1% of windows, respectively). In summary, the visual sense of clustering of high |iHS| scores in Figure 3 does indeed exceed the level of clustering expected under neutrality, supporting a model in which distinct selective events produce large |iHS| scores across discrete regions.

**Figure 5:**
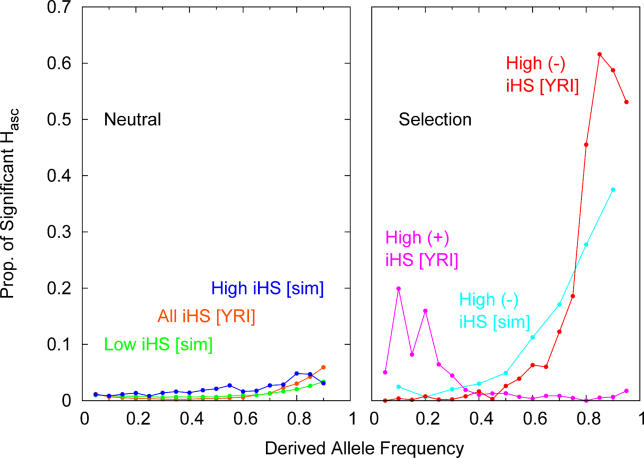
Strong Correlation between iHS and H_asc_ for the Yoruba Data The left-hand plot shows the probability that a 51-SNP window centered on a given SNP is in the lowest 1% of the empirical distribution for Fay and Wu's *H* in neutral simulated data, and in the Yoruba data overall. Notice that in neutral simulations, there is essentially no correlation between iHS and *H*. Right-hand plot: In contrast, in simulations with selection (cyan line, *s* = 100) there is a big increase in the rate of significant *H* values for high-frequency selected alleles with strongly negative iHS (<−2.5). The same pattern is seen for the real data (red line). In the real data, sites with strongly positive iHS (>2.5) show an increase in the rate of positive *H* scores at low derived allele frequencies (magenta line). The latter probably reflects instances of an ancestral allele hitchhiking to high frequency with a selected sweep.

Third, in our data, extreme iHS scores frequently occur in regions where the frequency spectrum also indicates the action of selection (Figure 5). As shown in Figure 2, a version of Fay and Wu's *H* test [37] for ascertained SNPs *(H_asc_)* provides a useful method for detecting sweeps where the selected site has reached high frequency. In simulations we find that high frequency selected sites tend to have both strongly negative iHS scores and strongly negative values of Fay and Wu's *H_asc_*. However in neutral simulations, iHS and *H_asc_* are essentially uncorrelated. In the Yoruba data, as many as one half of high frequency–derived SNPs with large iHS scores fall into the most extreme 1% of windows for *H_asc_*, genome-wide. This correlation argues strongly that many of our extreme iHS scores are in fact the result of positive selection. In our data, an excess of significant *H_asc_* scores is also seen for low-frequency alleles with positive iHS scores. This probably results from ancestral alleles that have hitchhiked to high frequency. Similar, but weaker, correlations are present in both the European and East Asian data (unpublished data).

Fourth, there is a highly significant enrichment for extreme iHS values in genic regions (*p* < 10^−20^ in all populations; Table S1). This is to be expected if selection occurs most often in (or near) genes, though one might not expect a dramatic difference in rates, since simulated selective events tend to produce signals over quite wide regions which would include both genic and non-genic SNPs. The proportion of SNPs with |iHS| > 2 is 1.23-fold higher in genic SNPs than non-genic SNPs in Yoruba, 1.16-fold higher in Europeans, and 1.13-fold higher in East Asians. A further enrichment in extreme iHS values is found in SNPs in overlapping genes compared with SNPs in non-overlapping genes. To check that these results are not an artifact of the higher HapMap SNP density in genes, we thinned genic SNPs at random so that the proportion of genic SNPs matched the proportion of the genome containing genes. After reanalysis, the difference between genic and nongenic iHS values remained about the same as before (unpublished data).

Last, various regions identified previously as likely targets of sweeps-in-progress are detected by our survey, including signals in Europeans in the lactase region [6,33], in the 17q21 inversion [20], and at CYP3A5 [7]; in the ADH cluster on Chromosome 4 in East Asians [38]; and in olfactory receptor clusters on Chromosomes 11p15 and 11q11 in Yoruba [39]. However, we do not detect all previously identified selection candidates, for example failing to find signals at G6PD in Yoruba [3,5], and in two genes involved in brain development that were recently reported to be under recent positive selection [8,9].

### Overview of Selection Regions

**Figure 6:**
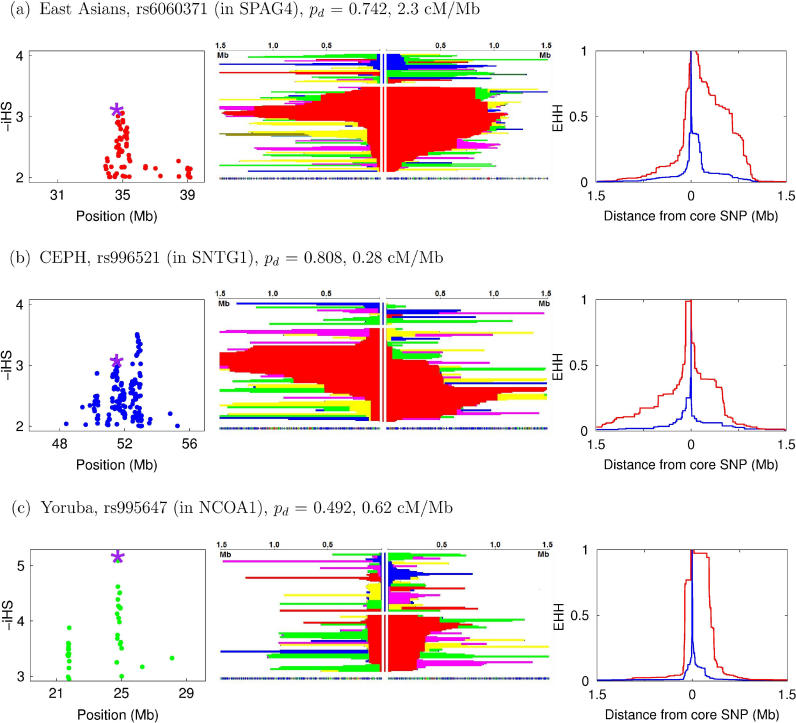
Signals of Selection for Three Candidate Selection Regions Discussed in the Text The columns show (left) scatter plots of negative iHS scores, (center) haplotype plots, and (right) decay of haplotype homozygosity. In each case the core SNP for the center and right-hand plots was chosen as a SNP with high negative iHS score (starred in the scatter plots); the allele marked in red is derived. For each signal, values are listed for the derived allele frequency (*p_d_*) and the local deCode recombination rate estimate.

To identify the strongest signals of selection, we divided the genome into non-overlapping windows of 100 kb. In this analysis windows were defined by physical location to facilitate the comparison of signals across populations. For each population, we identified the windows in the highest 1% of the empirical distribution for proportion of SNPs with |iHS| > 2. The positions of these windows on Chromosome 2 are indicated by vertical bars below each panel of Figure 3. Henceforth, we consider these windows to be candidates for containing selective sweeps. We find that 8 of 14 genomic regions listed as selection candidates by the HapMap Project are among the top 5% of our signals (Table 10 in [18]). A summary of some of our strongest signals is shown in Table 1, and a complete list is provided online in Protocols S1–S3). As an illustration, Figure 6 depicts the haplotype patterns, decay of EHH with distance, and plot of iHS scores for three strong candidate regions identified by our genome-wide scan.

**Table 1:**
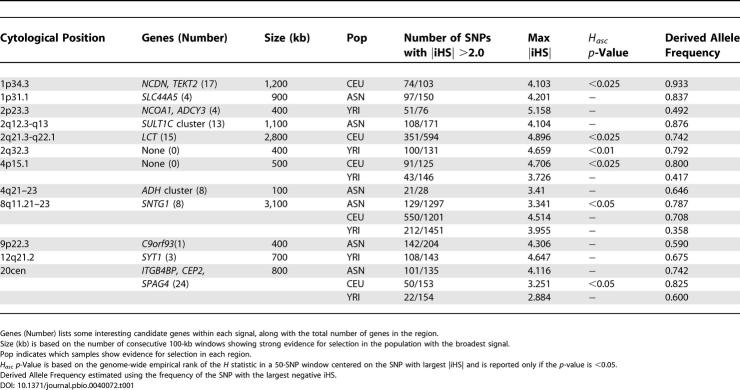
Summary of Some of the Strongest iHS Signals Genome-Wide

Analysis of the haplotype structure in candidate selection regions indicates that the events detected are typically very recent. We calculated the lengths of the haplotypes around the SNP with the largest negative iHS value in each region (Table S2). The average total distance between the first point to the left, and to the right of the core SNP at which EHH on the selected haplotypes drops below 0.25 is 0.52 cM in both East Asians and Europeans, and 0.32 cM in Yoruba. Hence, candidate sweep regions tend to be narrower in Yoruba than in the non-African populations, indicating that typical sweep events may be substantially younger in the non-African populations. (The size of the area affected by a strong sweep depends only weakly on the effective population size [40] and so the larger effective population size in Yoruba is not an explanation of the smaller average sweep size.)

A fully rigorous estimation of the ages of the candidate sweeps is difficult with the current data. However, making the simplistic assumption of a star-shaped genealogy for the favored haplotypes and assuming a generation time of 25 y, suggests average ages of ≍6,600 years and ≍10,800 years in the non-African, and African populations, respectively (Materials and Methods). Simulations using SelSim (Materials and Methods) suggest that these haplotype spans are consistent with selection coefficients of 0.01–0.04, assuming a population size of 10,000. These estimated ages should not be taken to imply a burst of selection at a particular time; instead, these ages and selection coefficients might represent areas in the parameter space in which we have good power. The longest haplotypes around derived alleles at >50% frequency extend 1.39 cM in East Asians (near the Gaucher disease gene, *GBA*), 1.25 cM in Europeans (near *NKX2–2,* which is involved in insulin regulation), and 0.97 cM in Yoruba (in a gene desert on Chromosome 5p15). These long haplotypes indicate extremely strong selection on recent mutations, though it is difficult to be confident about the actual genetic target of the selective events. In summary, the selection events that we detect are generally very recent, substantially postdating the separation times of these populations, and falling mainly within the agricultural phase of human evolution.

**Figure 7:**
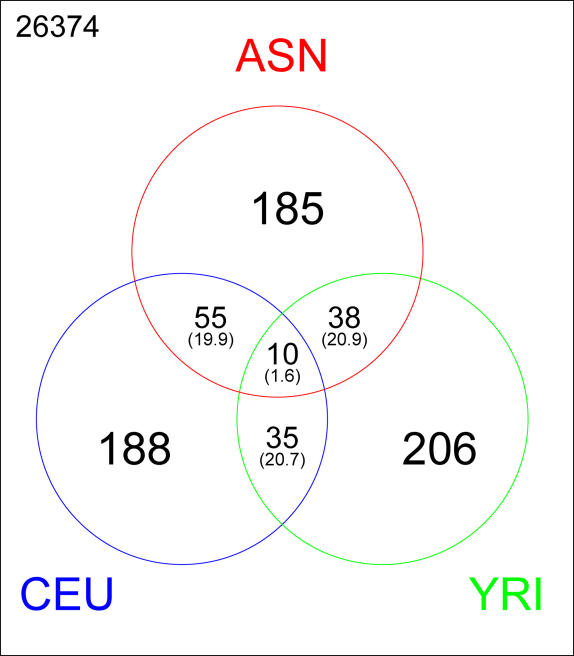
Sharing of iHS Signals between Populations The numbers listed inside circles represent the numbers of 100-kb windows that are in the top 1% of the empirical distributions in at least one population. The numbers in the intersection regions are in the top 1% for one population, and the top 5% for one or both of the other populations. The counts that would be expected if signals were independent across populations are shown in parentheses. The number of windows not in any circle is reported in the upper-left corner.

Figure 7 shows the extent of overlap of sweep regions across the three populations. Most of the sweep regions are found in only one of the three populations, consistent with the estimates indicating that these events generally postdate population separation. Nonetheless, there is a clear excess of sweeps that are shared between pairs of populations, or among all three populations. In principle, sharing of signals between populations might also be due to haplotypes that are inherited from the ancestral populations. However, this is probably a small effect since such unusually long haplotypes would be unlikely to survive the effects of recombination for >1,000 generations, separately in each population. Instead, the data suggest that most of the selective events that we detect are local to a single population, but that a significant fraction of the selective events are experienced by more than one population.

This view is further supported by the relationship between iHS and allele frequency divergence measured using *F_ST_* (Figures S26–S28). SNPs with high |iHS| in one population, but low |iHS| in another are likely to have high *F_ST_*, indicating that the SNP has changed frequency rapidly since population separation. Among the modest number of SNPs that have extreme positive iHS or extreme negative iHS in both populations, there is not an excess of high *F_ST_*, perhaps due to recent gene flow of alleles (and haplotypes) that are favored in multiple populations.

### Types of Genes under Selection

Next, we modified our analysis to study what types of genes are most commonly involved in recent adaptation. For every gene we determined the number of SNPs with high |iHS| in a 50 SNP window centered on the gene (Materials and Methods). The genes in the upper 10% of the empirical distribution for number of significant SNPs were then considered to be candidate targets of selection. Our procedure was designed to be robust to variation in gene size and SNP density across gene categories.

The PANTHER Gene Ontology database provides a classification of genes into 222 nested categories according to biological process [41]. We tested whether any of these categories showed an enrichment of genes with signals of recent selection (Materials and Methods). In this analysis, one might be concerned that there is low power to detect enrichment, since the expected counts for many categories are low, despite considering such a large fraction (10%) of the genes as candidates for selection.

Nonetheless, several categories show up as strongly significant in one or more populations (Table 2), including the related categories of chemosensory perception and olfaction; as well as gametogenesis, spermatogenesis, and fertilization. These types of processes have been identified as targets of selection in previous studies of human-chimpanzee divergence [10,11]. Overall, there is a modest enrichment for genes that show signals both of very recent selection in our study, and selection on the human lineage as a whole [10] (Table S3).

**Table 2:**
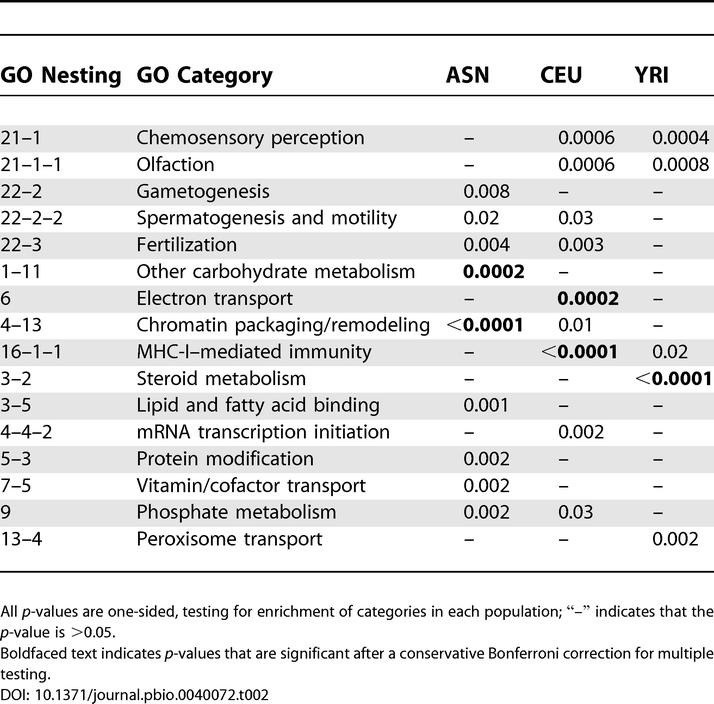
*p*-Values for Enrichment of GO Categories among Genes Showing Evidence for Partial Sweeps

In addition to the areas of overlap, we find enrichment in new categories not previously identified as targets of selection, including categories related to metabolism of carbohydrates, lipids, and phosphates, as well as vitamin transport. For some categories, the *p*-values are imprecise as there is clustering of related genes that are all significant (Table S4). We now describe in greater detail some of the categories of gene functions that show enrichment of selective signals. Except when noted, OMIM or EntrezGene provide references for the gene information given below [42,43]. The genes listed below are generally in the top 1%, and all are in the top 5% of signals in the relevant populations. However, some caution is required since the strongest signals often span both the target of selection as well as neighboring genes.

Recent reports have shown that genes involved in fertility and reproduction are subject to rapid adaptive evolution in primates due to sexual competition and perhaps defense against pathogens [10,11,44]. We observe signals for selection targeting several aspects of fertility and reproduction, including the basic protein structure of sperm (*RSBN1* in East Asians and Yoruba), sperm motility (*SPAG4* in Europeans and East Asians; *ODF2* in Europeans), sperm and egg viability (*ACVR1* in Europeans, CPEB2 in Yoruba), regulation of the female immune response to sperm (*TGM4*), egg fertilization (the *CRISP* gene cluster near 6p21.3 in Europeans), and testis determination (*NR0B1* in Europeans).

Some of the strongest signals of recent selection appear in various types of genes related to morphology. For example, four genes involved in skin pigmentation show clear evidence of selection in Europeans *(OCA2, MYO5A, DTNBP1, TYRP1)*. All four genes are associated with Mendelian disorders that cause lighter pigmentation or albinism, and all are in different genomic locations, indicating the action of separate selective events. One of these genes, *OCA2,* is associated with the third longest haplotype on a high frequency SNP anywhere in the genome for Europeans. A fifth gene, *SLC24A5,* has recently been shown by another group to impact skin pigmentation and to have a derived, selected allele near fixation in Europeans [45]. Though iHS has reduced power for alleles near fixation, SNPs near this gene also show strong iHS signals in Europeans (Table S2).

Various genes involved in skeletal development have also been targets of recent selection. Three related proteins involved in bone morphogenesis show signals of selection in Europeans (*BMP3* and *BMPR2*) and in East Asians (*BMP5*). In addition, *GDF5,* a gene in which mutations cause skeletal malformations, shows strong signals of selection in both Europeans and East Asians. Other morphological features also appear to be targets of selection, including hair formation and patterning in Yoruba (the keratin cluster near 17q12; and *FZD6*).

An important type of selective pressure that has confronted modern humans is the transition to novel food sources with the advent of agriculture and the colonization of new habitats [19,21]. As noted above, we see a strong signal of selection in the alcohol dehydrogenase *(ADH)* cluster in East Asians, including the third longest haplotype around a high frequency allele in East Asians. A variety of genes involved in carbohydrate metabolism have evidence for recent selection, including genes involved in metabolizing mannose (*MAN2A1* in Yoruba and East Asians), sucrose (*SI* in East Asians), and lactose (*LCT* in Europeans). Processing of dietary fatty acids is another system with signals of strong selection, including uptake (*SLC27A4* and *PPARD* in Europeans), oxidation (*SLC25A20* in East Asians) and regulation (NCOA1 in Yoruba and *LEPR* in East Asians). The latter gene *(LEPR)* is the leptin receptor and plays an important role in regulating adipose tissue mass.

Recent articles have proposed that genes involved in brain development and function may have been important targets of selection in recent human evolution [8,9]. While we do not find evidence for selection in the two genes reported in those studies *(MCPH1* and *ASPM),* we do find signals in two other microcephaly genes, namely, *CDK5RAP2* in Yoruba, and *CENPJ* in Europeans and East Asians [46]. Though there is not an overall enrichment for neurological genes in our gene ontology analysis, several other important brain genes also have signals of selection, including the primary inhibitory neurotransmitter *GABRA4,* an Alzheimer's susceptibility gene *PSEN1,* and *SYT1* in Yoruba; the serotonin transporter *SLC6A4* in Europeans and East Asians; and the dystrophin binding gene *SNTG1* in all populations.

Several other biological processes that have not previously been proposed as targets of selection also show an enrichment for signals of selection. For example, the category of electron transport genes is significant in Europeans, due in large part to selection in *CYP* genes. *CYP* genes are mainly expressed in the liver and catalyze many reactions involved in breaking down foreign compounds, including the majority of pharmaceutical agents. Genes in this class with evidence for selection include four genes in the *CYP450* gene cluster on Chromosome 1p33, as well as *CYP* genes in other genomic locations including *CYP3A5, CYP2E1,* and *CYP1A2*. Another category showing enrichment of selection signals is phosphate metabolism in East Asians and Europeans. Genes in the phosphatidylinositol pathway seem to be particularly overrepresented among the significant genes in this category, including *INPP5E, PI4K2B, IHPK1, IHPK2, IHPK3* in East Asians and *IMPA2* and *SYNJ1* in Europeans.

Curiously, Yoruba appears to have a greater number of signals on the X chromosome that map to genes compared with the other two populations. For example, the top 1% of 100-kb windows contain 15 genes in YRI (the largest of these windows containing only four genes), but six genes and two genes in Europeans and East Asians, respectively.

## Discussion

In this paper we provide the first genome-wide map of incomplete selective sweeps in humans. We find widespread signals of selection in all three populations. These selective events are generally very recent, falling mainly within the Holocene era, and substantially postdating the separation of the three populations. Selective sweeps in Yoruba tend to be narrower and apparently older than in the non-African populations, perhaps explaining why previous low-resolution scans for selection have reported a deficit of selective events in African populations [13–16]. (Two of those studies also used African-American samples, in which it is possible that European admixture may dilute evidence for selection in Africans.) Though most selected regions are not shared across populations, there is still a clear excess of shared selective events. Indeed, since we have incomplete power to detect selection, it is likely that we tend to underestimate the degree of sharing across populations.

Our analysis of the types of genes involved in recent selection provides a first insight into the type of biological processes that have been targets of selection in the latest stages of our evolution. Some of these functions (especially olfaction and fertilization-related genes) have also been found to show signals of sustained selection over much longer evolutionary timescales. However, other classes of selected genes (for example the skin pigmentation genes and metabolic genes) likely reflect the process of adaptation to modern conditions and new environments. Nonetheless, gaining a full view of the kinds of selective pressures that have faced modern humans, and our biological adaptations to those pressures, remains a challenging problem. For many selective signals there is uncertainty about the actual genetic target. Even when the target is clear, the nature of the adaptation is often not. Hence, interpreting the story of human adaptation promises to be an interesting research area for years to come.

The tremendous shifts experienced by modern human populations in habitats, food sources, population densities, and pathogen exposures have surely led to direct selection pressures on medically relevant phenotypes. At least three of the regions that we identified as targets of selection have previously been associated with complex phenotypes, including *CYP3A5* (salt-sensitive hypertension), *ADH* (alcoholism susceptibility), and the 17q21 inversion (recombination rates and fertility) [7,20,47]. These examples form part of an emerging pattern linking selection with complex trait phenotypes [21,22], as first proposed for diabetes in the “thrify genotype” hypothesis [19]. Therefore, selected haplotypes identified by our scan can be thought of as “selection candidates” for involvement in complex traits. With this in mind, we have developed an online tool for evaluating iHS at any HapMap SNP, and we provide files of tag-SNPs that can be used in genome-wide association studies to tag all the haplotypes with the strongest selection signals in each population.

## Materials and Methods

### HapMap project data information

All analysis is based on the HapMap Project Phase I/rel#16a datafiles (http://www.hapmap.org) [18]. For the CEU and YRI samples, we analyzed the data from the 60 unrelated parents. Due to their close genetic similarity, and in order to have a single larger sample, we pooled the CHB (Han Chinese from Beijing) and JPT (Japanese from Tokyo) samples to form a single sample of 89 unrelated Asian individuals, denoted as the ASN sample. Haplotype phase estimation for all the data was performed by the HapMap consortium using Phase 2.0 [18,23]. The phasing procedure also imputed all missing genotypes at SNPs with <20% missing data. Phase estimation by this method tends to be extremely accurate when parent-offspring trio data are available (CEU and YRI), and fairly accurate when all individuals are unrelated (ASN) [18,23]. In total, the numbers of polymorphic SNPs analyzed for each population were: 791,208 (ASN), 849,575 (CEU), and 885,926 (YRI).

### SNP annotation information

Annotation information was obtained from dbSNP build 123 for all SNPs, including physical positions (for genome build #34) and strand orientation. We also used the dbSNP classification of SNP functional states: non-synonymous, synonymous, within an intron or mRNA UTR, or within 2 kb of a gene (locus region). SNPs in any of these functional classes were considered genic. All other SNPs were considered nongenic.

### Obtaining ancestral states from dbSNP and chimp sequence alignment

For ∼30% of the SNPs, estimates of the ancestral state were already available from dbSNP using ftp://ftp.ncbi.nih.gov/snp/database/b124/mssql/data/SNPAncestralAllele.bcp.gz and an allele reference table at ftp://ftp.ncbi.nih.gov/snp/database/b124/mssql/data/Allele.bcp.gz. To obtain ancestral states for all SNPs, we obtained FASTA sequences for each SNP as specified for that rs entry in the dbSNP database, and aligned those to the draft genome build of the chimp sequence, obtained from the UCSC database [12] using blat, version 27 [48]. For each SNP, we selected the overall best alignment, preferring alignments with the highest overall blat score, and preferring alignments mapping to unique chimp chromosome over random or unmapped sequences when possible. We then inferred the ancestral state as the chimp allele at the appropriate position in the sequence, provided that the sequence quality score was ≥20 at that site, and that it matched one of the human alleles. The agreement between our ancestral state estimates and those in dbSNP was 94.5%. For each SNP, we accepted an ancestral state if it could be obtained either from dbSNP or from our own analysis.

### Simulations overview

We simulated data under a variety of neutral and selection models using the coalescent programs ms (for neutral models) and SelSim (for selection) [49,50]. Overall, we aimed to match the simulated analysis to the real analysis as closely as possible. We performed separate simulations for each population sample, matching the numbers of sampled chromosomes, the site frequency spectrum, and the average spacing of markers in units of *ρ* = 4*Nr* to the data and considering a range of appropriate demographic models for each population based on previous literature. As detailed below, our approach to dealing with ascertainment bias was to simulate a moderately large number of candidate SNPs under an assumed demographic model, and then use rejection sampling to thin the SNPs to match the observed frequency spectrum and SNP density. The rejection sampling function serves implicitly as a model of the ascertainment process.

For every simulated dataset, we estimated genetic distances from the data (as described below) in order to calculate iHS values. For the East Asian sample (where haplotyping is most difficult due to the lack of genotype data from relatives), we also incorporated haplotype estimation into the simulations. For each model and combination of parameters, we simulated 1,000 independent regions. The lengths of simulated regions averaged 500 units of *ρ* (a little more than 1 Mb at the genome-average recombination rate).

### Neutral demographic models

For each population we explored a range of demographic scenarios supported by the literature as plausible models for each population [16,35,36,51,52]. The models considered here spanned the range of models in the confidence sets identified by [35] for closely related populations. We treat the Hausa results from that study as being appropriate for Yoruba because Hausa and Yoruba are genetically very closely related. For the non-African populations, we simulated a family of bottleneck models where a population of constant size *N_A_* instantaneously shrinks in size to b · *N_A_* at time *t_start_* generations before the present. The population remains at that size for *t_dur_* generations and then instantaneously recovers to either its original size or to a larger (or smaller) size. For the African population, we considered models in which an ancestral population at equilibrium size *N_A_* grows exponentially beginning *t_onset_* generations in the past at rate α, such that the present population size is *N_A_e* ^α*t*_*onset*_^ [34]. The ms command lines corresponding to each demographic model simulated are provided in Protocol S4.

### Modeling variation in recombination rate

Since recombination rate variation plays an important role in determining patterns of LD [30–32], our simulations allowed for recombination hotspots, and modeled variation both in the background rate and in the spacing and intensity of hotspots. Hotspots were assumed to occur as a Poisson process, at an average rate of 1 hotspot per 100 kb [30,31]. The intensity of hotspots was modeled as a gamma distribution, with the mean intensity 28-fold (and the median 20-fold) the background rate, roughly matching that observed [30]. Hotspot length was gamma distributed with mean 2 kb. The background recombination rate was modeled with a gamma distribution, such that the total rate of recombination events in the background and in hotspots matched the average estimated rate from the data, for each population.

### Modeling SNP ascertainment

Due to SNP ascertainment, the allele frequency spectrum in the HapMap differs greatly from the frequency spectrum in DNA sequence data. In all three samples there is a deficit of rare variants, and an excess of high frequency variants. Moreover, the actual frequency distributions are substantially different among the three samples (Figure S29). Since SNP ascertainment can have a major impact on analysis, it is important to model this in the simulations. One potential approach would be to model the ascertainment procedure explicitly, and to attempt to recreate the observed frequency spectrum [52,53]. However, this is very difficult to do in a fully convincing way for the present data, since the full history of ascertainment of SNPs into dbSNP and from there into HapMap is complex and not well-documented.

Thus, as a practical alternative, we simulated data with complete ascertainment and then used rejection sampling to obtain the observed frequency spectrum (separately for each population sample). In effect, the rejection sampling function serves as an empirical model of the unknown ascertainment process. Let *f(a)* be the probability that a SNP in our dataset has *a* copies of the derived allele (i.e., *f(a)* is the observed frequency spectrum), where 0 < *a* < *n* and *n* is the total haploid sample size. Let *π(a)* be the corresponding frequency spectrum in a neutral model with complete SNP ascertainment, assuming a specified demographic model. To achieve the observed frequency spectrum, we simulated data from the chosen demographic model with complete ascertainment, and then retained sites with *a* copies of the derived allele with probability *f(a)/(cπ(a)),* where *c = max*[*f(a)/π(a)*]. The mutation rate was chosen so that after ascertainment the expected SNP density would match the observed density.

### Model for selective sweeps

We used the program *SelSim* to simulate data with selection and recombination [50]. The model assumes that a new mutation at a specified position in the sequence experiences a constant additive selection pressure *σ* = 2*NS* where *N* is the population size and *s* is the additive selective advantage per copy per generation. The data are sampled when the mutation reaches a specified frequency, or reaches fixation. The program only accommodates models of constant population size and hence is most appropriate for modeling the Yoruba (for whom a constant-size model falls within the confidence set of [35]). For each *σ,* we fixed the frequency of the selected site to *p* = 0.1, 0.2, … , 0.9, 1.0 and generated samples of size *n* = 120. We also simulated a model of selection where the sample was taken 200 generations after the fixation of the beneficial allele. 1,000 repetitions were generated for each scenario. The recombination hotspot model and mutation/ascertainment scheme were as described above. The rejection weights for the SNP ascertainment were determined under the neutral model, so that when *σ* = 0 the frequency spectrum matches the data (on average).

### Haplotype phase uncertainty

For the simulations of the African and European data, we ignored uncertainty in haplotype phase since for those populations the availability of trio data makes the reconstructed haplotypes very accurate. For the East Asian data, the phasing error rates are higher since all the individuals are unrelated. To assess the impact of this, all of our simulation data for the East Asians were treated first as diploid genotypes, and then haplotype phase was estimated using a new, rapid-phasing algorithm, *fastphase,* kindly provided by Paul Scheet and Matthew Stephens. This algorithm uses a similar model and achieves nearly the same accuracy as Phase 2, which was used to phase the HapMap data [54].

### Estimation of a fine-scale genetic map

To generate a high-resolution, genome-wide genetic map, we implemented a version of the Li and Stephens algorithm [24]. That algorithm was designed for estimating recombination rates over fine scales based on haplotype data. As input data, we used the physical position of each SNP, along with the phased haplotypes. This was performed separately for each of the three population samples as well as for each repetition of simulated data. The output is a high-resolution estimate of a genetic map for each population, where each SNP is assigned a position in units of *ρ* = 4*Nr* where *N* is the effective population size and *r* is the recombination rate per generation.

Our implementation follows the original algorithm closely, except in three regards. First, we maximized the likelihood using the E.M. algorithm, finding that this converges quickly and reliably, even on very large datasets. Second, we performed local smoothing of the rates as follows: the recombination rate between SNPs *n* and *n* + 1 is estimated as the maximum likelihood rate in an interval that extends from 20 kb to the left of SNP *n* to 20 kb to the right of SNP *n* + 1. This window size was chosen as a suitable compromise between wanting to detect recombination hotspots, and not wanting to be overly misled by random noise in the data. The smoothing has the effect of spreading out hotspots, to some extent, but over a scale that is much less than the physical length of “interesting” haplotypes. Third, we did not implement the bias correction suggested by [24] because this depends in part on the spacing of markers, which varies widely in the data, and because the bias cancels out in our test ratio anyway. Simulations (unpublished data) indicate that our implementation performs well, having only slight bias for the parameters relevant to HapMap, and correctly identifying regions of rate variation. Over large scales it is also highly correlated with the deCODE genetic map [55]. There is a modest downward bias in the estimated rates in partial sweep regions, but in simulations this bias has little discernible effect on power. This type of bias will tend to be conservative, in the sense of reducing signal.

### Calculation of iHS

The iHS was computed as described earlier in this paper for every SNP with ancestral state information and with minor allele frequency >5%. To compute the iHH, what we do is equivalent to the following. We plot the decay of EHH to the left and right of the core SNP, for each allele, using all available SNPs. The *x*-axis is in units of genetic distance, 4*Nr,* estimated as described above. The EHH values at successive SNPs are joined by straight lines, and then we compute the total area under each curve, between the nearest points to the left and right of the core SNP where the EHH drops below 0.05. Since the population genetics of the X chromosome differs from that of the autosomes (e.g., due to smaller effective population size), we standardized the X chromosome separately from the autosomes, and excluded it from our overall genome-wide analyses.

The SNP data include occasional large gaps between successive SNPs. If the region spanned by EHH > 0.05 reached a chromosome end or the start of a gap >200 kb, then no iHS value was reported for the core SNP. If there was a smaller gap (20–200 kb), then the genetic distance spanned by the gap was reduced by a multiplicative factor 20/*g,* where *g* is the gap size in kb. This is an ad hoc fix designed to eliminate spurious signals produced by occasional large gaps, while minimizing the loss of data. After applying the various filters, we were left with 676,718, 701,633, and 621,915 SNPs with iHS scores in the European, Yorubans, and East Asian populations respectively.

### Summaries of the frequency spectrum

Both Tajima's D and Fay and Wu's H tests use summaries of the site frequency spectrum to identify signatures of selection [37,56]. However, these tests were designed for full-sequence data, and not for pre-ascertained SNPs. Nonetheless, we have tested the value of using these statistics to identify regions whose frequency spectra are strongly different from the bulk of the genome, suggesting the influence of selection. To compute these statistics on ascertained data, we treated the ascertained genotypes as if they were the complete sequence data, and then computed D and H in the usual way. The ascertainment-biased summary statistics are denoted *D_asc_* and *H_asc_* to indicate that they are not expected to follow the usual distributions. Since the ascertained data are enriched for high-frequency variants, the average *D_asc_* is highly positive, whereas the average *H_asc_* is highly negative. We obtained empirical distributions of both *D_asc_* and *H_asc_* in all 50-marker windows across the entire autosomal genome for each population. The empirical *p*-value for a particular window represents the proportional rank of the statistic in that window compared with the overall genome distribution (i.e., low *p*-values indicate a more negative D or H than the bulk of the empirical distribution).

### Identifying candidate signals of selection

In order to identify genes or genomic regions with signals of selection, we focused on windows of consecutive SNPs. Where possible, we used a standard window size of 50 SNPs. For analyses of genes with ≤50 SNPs, we created windows of 50 SNPs centered on each gene. For the ∼5% of genes with >50 SNPs, we treated the window as containing all SNPs in the gene. To study the overlap of signals across populations, we modified our analysis to consider fixed, nonoverlapping, 100-kb windows so that the window positions would correspond exactly across populations.

For both kinds of window definition, the size of signal in each region was quantified by the proportion of SNPs with |iHS| > 2. Simulations indicated that this criterion provides a powerful signal of selection. Separately for each population and analysis, we determined empirical cutoffs for the top 1%, 5%, etc., of signals genome-wide, and considered these strongest signals to indicate candidate selection regions. Since the numbers of SNPs varied considerably across the fixed 100-kb windows, we grouped windows into bins with similar numbers of SNPs, and determined empirical cutoffs separately for each bin. Regions with <10 SNPs were dropped, as were bins with <20 regions. The X-chromosome data was analysed separately. The thresholds for empirical cutoffs for the X chromosome were based on the autosomal cutoffs. 100-kb windows and genes in the X chromosome that were above these thresholds were considered to be significant.

### Estimating haplotype ages

To obtain a crude estimate of the ages of sweeps, we assumed (1) a star phylogeny for the selected haplotypes, (2) that the two chromosomes which descended from a common ancestor and carry a selected allele are identical between the core SNP and the point of the nearest recombination event on either lineage, (3) that the two chromosomes start to differ immediately beyond the nearest recombination event. Hence, Pr[Homoz] = *e* ^−2*rg*^ where Pr[Homoz] is the probability that two chromosomes are homozygous at a recombination distance *r* from the selected site, given a common ancestor *g* generations before the present. Taking the generation time to be 25 y, the ancestor time in years becomes *t* = 25*g*. Then, for example in Africans, when Pr[Homoz] = 0.25, we observed that 2*r* = 0.32% (the distances in the text are the sum of *r* in both directions). Then *t* = −(25)(100)ln(.25/.32 ≍ 10,000 years. A similar calculation was performed for the other populations.

### Gene ontology analysis

The PANTHER Gene Ontology database was downloaded from https://panther.appliedbiosystems.com in March 2005. For each PANTHER “biological-process,” category we determined both the total number of genes, and the number of genes in the top 10% of empirical signals (defined as above). One-tailed *p*-values for enrichment of particular categories were obtained either using the standard Chi-square approximation if the expected number of counts exceeded five, or otherwise by exact enumeration of the binomial probabilities. We used a Bonferroni correction to assess *overall* experiment-wide significance, though this is likely to be particularly conservative in this case since the 222 PANTHER categories are partially nested and overlapping.

### Haplotype plotting

An online browser providing haplotype plots and iHS scores for all HapMap SNPs is at http://pritch.bsd.uchicago.edu/data.html.

## Supporting Information

Figure S1 Power to Detect Selection Using Various Test Statistics (3 KB PDF).

Figure S2 Single versus Multipoint iHS Power Plot (2 KB PDF).

Figure S3 Plots of Chromosome 1 SNPs with Extreme iHS Values Illustrate Discrete Clusters of Signals (118 KB PDF).

Figure S4 Plots of Chromosome 2 SNPs with Extreme iHS Values Illustrate Discrete Clusters of Signals (174 KB PDF).

Figure S5 Plots of Chromosome 3 SNPs with Extreme iHS Values Illustrate Discrete Clusters of Signals (122 KB PDF).

Figure S6 Plots of Chromosome 4 SNPs with Extreme iHS Values Illustrate Discrete Clusters of Signals (120 KB PDF).

Figure S7 Plots of Chromosome 5 SNPs with Extreme iHS Values Illustrate Discrete Clusters of Signals (91 KB PDF).

Figure S8 Plots of Chromosome 6 SNPs with Extreme iHS Values Illustrate Discrete Clusters of Signals (116 KB PDF).

Figure S9 Plots of Chromosome 7 SNPs with Extreme iHS Values Illustrate Discrete Clusters of Signals (85 KB PDF).

Figure S10 Plots of Chromosome 8 SNPs with Extreme iHS Values Illustrate Discrete Clusters of Signals (129 KB PDF).

Figure S11 Plots of Chromosome 9 SNPs with Extreme iHS Values Illustrate Discrete Clusters of Signals (144 KB PDF).

Figure S12 Plots of Chromosome 10 SNPs with Extreme iHS Values Illustrate Discrete Clusters of Signals (76 KB PDF).

Figure S13 Plots of Chromosome 11 SNPs with Extreme iHS Values Illustrate Discrete Clusters of Signals (62 KB PDF).

Figure S14 Plots of Chromosome 12 SNPs with Extreme iHS Values Illustrate Discrete Clusters of Signals (92 KB PDF).

Figure S15 Plots of Chromosome 13 SNPs with Extreme iHS Values Illustrate Discrete Clusters of Signals (58 KB PDF).

Figure S16 Plots of Chromosome 14 SNPs with Extreme iHS Values Illustrate Discrete Clusters of Signals (44 KB PDF).

Figure S17 Plots of Chromosome 15 SNPs with Extreme iHS Values Illustrate Discrete Clusters of Signals (45 KB PDF).

Figure S18 Plots of Chromosome 16 SNPs with Extreme iHS Values Illustrate Discrete Clusters of Signals (31 KB PDF).

Figure S19 Plots of Chromosome 17 SNPs with Extreme iHS Values Illustrate Discrete Clusters of Signals (31 KB PDF).

Figure S20 Plots of Chromosome 18 SNPs with Extreme iHS Values Illustrate Discrete Clusters of Signals (79 KB PDF).

Figure S21 Plots of Chromosome 19 SNPs with Extreme iHS Values Illustrate Discrete Clusters of Signals (21 KB PDF).

Figure S22 Plots of Chromosome 20 SNPs with Extreme iHS Values Illustrate Discrete Clusters of Signals (27 KB PDF).

Figure S23 Plots of Chromosome 21 SNPs with Extreme iHS Values Illustrate Discrete Clusters of Signals (37 KB PDF).

Figure S24 Plots of Chromosome 22 SNPs with Extreme iHS Values Illustrate Discrete Clusters of Signals (51 KB PDF).

Figure S25 Plots of the X Chromosome SNPs with Extreme iHS Values Illustrate Discrete Clusters of Signals (67 KB PDF).

Figure S26 Plot Comparing Fst with iHS (Europeans and East Asians) (17 KB PDF).

Figure S27 Plot Comparing Fst with iHS (East Asians and Yoruba) (17 KB PDF).

Figure S28 Plot Comparing Fst with iHS (Europeans and Yoruba) (17 KB PDF).

Figure S29 Polarized Frequency Spectra for Each HapMap Population (15 KB PDF).

Figure S30 Combined Supporting Information File (2.0 MB PDF).

Protocol S1 Summary of Strongest Regions of Selection Genome-Wide in East Asians (42 KB TXT).

Protocol S2 Summary of Strongest Regions of Selection Genome-Wide in Europeans (44 KB TXT).

Protocol S3 Summary of Strongest Regions of Selection Genome-Wide in Yoruba (46 KB TXT).

Protocol S4 Demographic Simulation Information Supplement (2.2 MB DOC).

Table S1 iHS Signal in Genic versus Non-Genic Regions (135 KB DOC).

Table S2 Genetic Spans of the Longest Haplotypes in Each Population (49 KB DOC).

Table S3 Comparison with Clark et al. 2003 Study (60 KB PDF).

Table S4 Gene Clusters That Contribute to Significant GO Categories (42 KB DOC).

## Abbreviations

ASN: East Asian(s)
CEU: northern and western European(s)
EHH: extended haplotype homozygosity
iHH: integrated EHH
iHS: integrated haplotype score
SNP: single nucleotide polymorphism
YRI: Yoruba

## References

[pbio-0040072-b001] Diamond J (2002). Evolution, consequences and future of plant and animal domestication. Nature.

[pbio-0040072-b002] Jobling MA, Hurles ME, Tyler-Smith C (2004). Human evolutionary genetics: Origins, peoples and disease.

[pbio-0040072-b003] Tishkoff SA, Varkonyi R, Cahinhinan N, Abbes S, Argyropoulos G (2001). Haplotype diversity and linkage disequilibrium at human G6PD: Recent origin of alleles that confer malarial resistance. Science.

[pbio-0040072-b004] Hamblin MT, Thompson EE, Di Rienzo A (2002). Complex signatures of natural selection at the Duffy blood group locus. Am J Hum Genet.

[pbio-0040072-b005] Sabeti PC, Reich DE, Higgins JM, Levine HZP, Richter DJ (2002). Detecting recent positive selection in the human genome from haplotype structure. Nature.

[pbio-0040072-b006] Bersaglieri T, Sabeti PC, Patterson N, Vanderploeg T, Schaffner SF (2004). Genetic signatures of strong recent positive selection at the lactase gene. Am J Hum Genet.

[pbio-0040072-b007] Thompson EE, Kuttab-Boulos H, Witonsky D, Yang L, Roe BA (2004). CYP3A variation and the evolution of salt-sensitivity variants. Am J Hum Genet.

[pbio-0040072-b008] Evans PD, Gilbert SL, Mekel-Bobrov N, Vallender EJ, Anderson JR (2005). Microcephalin, a gene regulating brain size, continues to evolve adaptively in humans. Science.

[pbio-0040072-b009] Mekel-Bobrov N, Gilbert SL, Evans PD, Vallender EJ, Anderson JR (2005). Ongoing adaptive evolution of ASPM, a brain size determinant in Homo sapiens. Science.

[pbio-0040072-b010] Clark AG, Glanowski S, Nielsen R, Thomas PD, Kejariwal A (2003). Inferring nonneutral evolution from human-chimp-mouse orthologous gene trios. Science.

[pbio-0040072-b011] Nielsen R, Bustamante C, Clark AG, Glanowski S, Sackton TB (2005). A scan for positively selected genes in the genomes of humans and chimpanzees. PLoS Biol.

[pbio-0040072-b012] Chimpanzee Sequencing and Analysis Consortium (2005). Initial sequence of the chimpanzee genome and comparison with the human genome. Nature.

[pbio-0040072-b013] Kayser M, Brauer S, Stoneking M (2003). A genome scan to detect candidate regions influenced by local natural selection in human populations. Mol Biol Evol.

[pbio-0040072-b014] Akey JM, Eberle MA, Rieder MJ, Carlson CS, Shriver MD (2004). Population history and natural selection shape patterns of genetic variation in 132 genes. PLoS Biol.

[pbio-0040072-b015] Storz JF, Payseur BA, Nachman MW (2004). Genome scans of DNA variability in humans reveal evidence for selective sweeps outside of Africa. Mol Biol Evol.

[pbio-0040072-b016] Stajich JE, Hahn MW (2005). Disentangling the effects of demography and selection in human history. Mol Biol Evol.

[pbio-0040072-b017] Carlson CS, Thomas DJ, Eberle MA, Swanson JE, Livingston RJ (2005). Genomic regions exhibiting positive selection identified from dense genotype data. Genome Res.

[pbio-0040072-b018] The International HapMap Consortium (2005). A haplotype map of the human genome. Nature.

[pbio-0040072-b019] Neel JV (1962). Diabetes mellitus: A “thrifty” genotype rendered detrimental by “progress”?. Am J Hum Genet.

[pbio-0040072-b020] Stefansson H, Helgason A, Thorleifsson G, Steinthorsdottir V, Masson G (2005). A common inversion under selection in Europeans. Nat Genet.

[pbio-0040072-b021] Di Rienzo A, Hudson RR (2005). An evolutionary framework for common diseases: the ancestral-susceptibility model. Trends Genet.

[pbio-0040072-b022] Vander Molen J, Frisse LM, Fullerton SM, Qian Y, Del Bosque-Plata L (2005). Population genetics of CAPN10 and GPR35: Implications for the evolution of type 2 diabetes variants. Am J Hum Genet.

[pbio-0040072-b023] Stephens M, Scheet P (2005). Accounting for decay of linkage disequilibrium in haplotype inference and missing-data imputation. Am J Hum Genet.

[pbio-0040072-b024] Li N, Stephens M (2003). Modeling linkage disequilibrium and identifying recombination hotspots using single-nucleotide polymorphism data. Genetics.

[pbio-0040072-b025] Hudson RR, Bailey K, Skarecky D, Kwiatowski J, Ayala FJ (1994). Evidence for positive selection in the superoxide dismutase (Sod) region of Drosophila melanogaster. Genetics.

[pbio-0040072-b026] Kirby DA, Stephan W (1995). Haplotype test reveals departure from neutrality in a segment of the white gene of Drosophila melanogaster. Genetics.

[pbio-0040072-b027] Andolfatto P, Wall JD, Kreitman M (1999). Unusual haplotype structure at the proximal breakpoint of In(2L)t in a natural population of Drosophila melanogaster. Genetics.

[pbio-0040072-b028] Slatkin M, Bertorelle G (2001). The use of intraallelic variability for testing neutrality and estimating population growth rate. Genetics.

[pbio-0040072-b029] Toomajian C, Kreitman M (2002). Sequence variation and haplotype structure at the human HFE locus. Genetics.

[pbio-0040072-b030] McVean GA, Myers SR, Hunt S, Deloukas P, Bently DR (2004). The fine-scale structure of recombination rate variation in the human genome. Science.

[pbio-0040072-b031] Crawford DC, Bhangale T, Li N, Hellenthal G, Rieder MJ (2004). Evidence for substantial fine-scale variation in recombination rates across the human genome. Nat Genet.

[pbio-0040072-b032] Myers S, Bottolo L, Freeman C, McVean G, Donnelly P (2005). A fine-scale map of recombination rates and hotspots across the human genome. Science.

[pbio-0040072-b033] Coelho M, Luiselli D, Bertorelle G, Lopes AI, Seixas S (2005). Microsatellite variation and evolution of human lactase persistence. Hum Genet.

[pbio-0040072-b034] Pluzhnikov A, Di Rienzo A, Hudson RR (2002). Inferences about human demography based on multilocus analyses of noncoding sequences. Genetics.

[pbio-0040072-b035] Voight BF, Adams A, Frisse L, Quan Y, Hudson RR (2005). Interrogating multiple aspects of variation in a full re-sequencing data set to infer human population size changes. PNAS.

[pbio-0040072-b036] Reich DE, Cargill M, Bolk S, Ireland J, Sabeti PC (2001). Linkage disequilibrium in the human genome. Nature.

[pbio-0040072-b037] Fay JC, Wu CI (2000). Hitchhiking under positive Darwinian selection. Genetics.

[pbio-0040072-b038] Osier MV, Pakstis AJ, Soodyall H, Comas D, Goldman D (2002). A global perspective on genetic variation at the ADH genes reveals unusual patterns of linkage disequilibrium and diversity. Am J Hum Genet.

[pbio-0040072-b039] Gilad Y, Bustamante CD, Lancet D, Paabo S (2003). Natural selection on the olfactory receptor gene family in humans and chimpanzees. Am J Hum Genet.

[pbio-0040072-b040] Maynard Smith J, Haigh J (1974). The hitch-hiking effect of a favourable gene. Genet Res Camb.

[pbio-0040072-b041] Thomas PD, Kejariwal A, Campbell MJ, Mi H, Diemer K (2003). PANTHER: A browsable database of gene products organized by biological function, using curated protein family and subfamily classification. Nucleic Acids Res.

[pbio-0040072-b042] OMIM (2000). Online Mendelian Inheritance in Man. McKusick-Nathans Institute for Genetic Medicine, Johns Hopkins University and National Center for Biotechnology Information.

[pbio-0040072-b043] Maglott D, Ostell J, Pruitt KD, Tatusova T (2005). Entrez Gene: Gene-centered information at NCBI. Nucleic Acids Res.

[pbio-0040072-b044] Clark NL, Swanson WJ (2005). Pervasive adaptive evolution in primate seminal proteins. PLoS Genet.

[pbio-0040072-b045] Lamason RL, Mohideen M, Mest JR, Wong AC, Norton HL (2005). SLC24A5, a putative cation exchanger, affects pigmentation in zebrafish and humans. Science.

[pbio-0040072-b046] Bond J, Roberts E, Springell K, Lizarraga SB, Scott S (2005). A centrosomal mechanism involving CDK5RAP2 and CENPJ controls brain size. Nat Genet.

[pbio-0040072-b047] Chen C, Lu R, Chen Y, Wang M, Chang Y (1999). Interaction between the functional polymorphisms of the alcohol-metabolism genes in protection against alcoholism. Am J Hum Genet.

[pbio-0040072-b048] Kent WJ (2002). BLAT–the BLAST-like alignment tool. Genome Res.

[pbio-0040072-b049] Hudson RR (2002). Generating samples under a Wright-Fisher neutral model of genetic variation. Bioinformatics.

[pbio-0040072-b050] Spencer CCA, Coop G (2004). SelSim: A program to simulate population genetic data with natural selection and recombination. Bioinformatics.

[pbio-0040072-b051] Adams AM, Hudson RR (2004). Maximum-likelihood estimation of demographic parameters using the frequency spectrum of unlinked single-nucleotide polymorphisms. Genetics.

[pbio-0040072-b052] Marth GT, Czabarka E, Murvai J, Sherry ST (2004). The allele frequency spectrum in genome-wide human variation data reveals signals of differential demographic history in three large world populations. Genetics.

[pbio-0040072-b053] Nielsen R (2004). Population genetic analysis of ascertained SNP data. Hum Genomics.

[pbio-0040072-b054] Scheet P, Stephens M (2006). A fast and flexible statistical model for large-scale population genotype data: Applications to inferring missing genotypes and haplotypic phase. Am J Hum Genet.

[pbio-0040072-b055] Kong A, Gudbjartsson DF, Sainz J, Jonsdottir GM, Gudjonsson SA (2002). A high-resolution recombination map of the human genome. Nat Genet.

[pbio-0040072-b056] Tajima F (1989). Statistical method for testing the neutral mutation hypothesis by DNA polymorphism. Genetics.

