# Correction: A Map of Recent Positive Selection in the Human Genome

**DOI:** 10.1371/journal.pbio.0040154

**Published:** 2006-04-11

**Authors:** Benjamin F Voight, Sridhar Kudaravalli, Xiaoquan Wen, Jonathan K Pritchard

In *PLoS Biology*, volume 4, issue 3, DOI: 10.1371/journal.pbio.0040072

Because of a typesetting error, the symbol “σ” is incorrectly displayed as “s” in the legends of Figures 1, 2, and 5.

In the Results section, the sentence “These data consist of ∼800,000 polymorphic SNPs in a total of 309 unrelated individuals” should be “These data consist of ∼800,000 polymorphic SNPs in a total of 209 unrelated individuals.”

In the section “Widespread Signals of Recent Selection,” the sentence “For example, in simulations matching aspects of the Yoruba data, only 0.1% of windows of 50 consecutive SNPs had more than 16 SNPs with |iHS| > 20” should be “For example, in simulations matching aspects of the Yoruba data, only 0.1% of windows of 50 consecutive SNPs had more than 16 SNPs with |iHS| > 2.0.”

