# Correction: A Map of Recent Positive Selection in the Human Genome

**DOI:** 10.1371/journal.pbio.0050147

**Published:** 2007-06-12

**Authors:** Benjamin F Voight, Sridhar Kudaravalli, Xiaoquan Wen, Jonathan K Pritchard

In *PLoS Biology*, volume 4, issue 3: doi: 10.1371/journal.pbio.0040072

1. In the supplementary materials, the intended simulation models for the population growth scenarios in the Yoruba were incorrectly specified. The corrected command lines, matching the models that were the intended simulations, are as follows:

Exponential growth from initial size N_A_ to N_0_, occurring t_onset_ generations in the past.

N_A_ = 11156, N_0_ = 11156, t_onset_ = 1000: ms 120 1 -s **S** -r ρ L -G 0 -eG 0.02241 0.0

N_A_ = 10659, N_0_ = 22600, t_onset_ = 1000: ms 120 1 -s **S** -r ρ L -G 67.94 -eG 0.011062 0.0

N_A_ = 7834, N_0_ = 57900, t_onset_ = 4000: ms 120 1 -s **S** -r ρ L -G 115.81 -eG 0.017271 0.0

N_A_ = 10424, N_0_ = 938000, t_onset_ = 500: ms 120 1 -s **S** -r ρ L -G 33765.3 -eG 0.00013326 0.0

N_A_ = 9944, N_0_ = 44600, t_onset_ = 1500: ms 120 1 -s **S** -r ρ L -G 178.49 -eG 0.008408 0.0

N_A_ = 11020, N_0_0 = 23300, t_onset_ = 250: ms 120 1 -s **S** -r ρ L -G 279.13 -eG 0.002682 0.0

N_A_ = 9912, N_0_ = 199000, t_onset_ = 1000: ms 120 1 -s **S** -r ρ L -G 2387.65 -eG 0.0012563 0.0

N_A_ = 10018, N_0_ = 1910000, t_onset_ = 750: ms 120 1 -s **S** -r ρ L -G 53484.84 -eG 9.8167E-5 0.0

2. As a result of the above, Figure 4 as presented in the manuscript is incorrect, and the revision is presented below. The fundamental result and interpretation of the figure as they pertain to the manuscript are unchanged: The observed iHS scores for the Yoruban Hapmap sample are more extreme than under the (correctly) simulated growth models presented above.

We regret any confusion this may have caused readers.

## 

**Figure 1:**
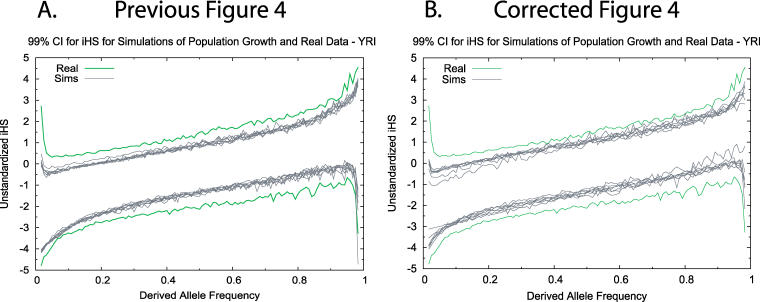
Revised Figure 4 (A) Previous published and (B) corrected simulation results for the central 99% range of unstandardized iHS for SNPs in the Yoruba data and for SNPs in matched neutral simulations, originally presented as Figure 4 in Voight et al. As before, the upper and lower lines mark the boundaries of the central 99% distribution of the unstandardized iHS ratio, as a function of derived allele frequency. The gray lines plot results for a range of plausible demographic models. Note the similarity of the revised figure (panel B) to the previously published figure 4 (panel A), which continue to demonstrate that the observed iHS scores for the Yoruban Hapmap sample are more extreme than under the (correctly) simulated growth models presented in panel B, above.

