# Supplementary material for: A Map of Recent Positive Selection in the Human Genome
Source: PLoS Biol. 2006 Mar 7;4(3):e72. doi: 10.1371/journal.pbio.0040072 (PMC1382018; doi:10.1371/journal.pbio.0040072)
Supplement: Supplementary file 1 [file pbio.0040072.sg001.pdf]

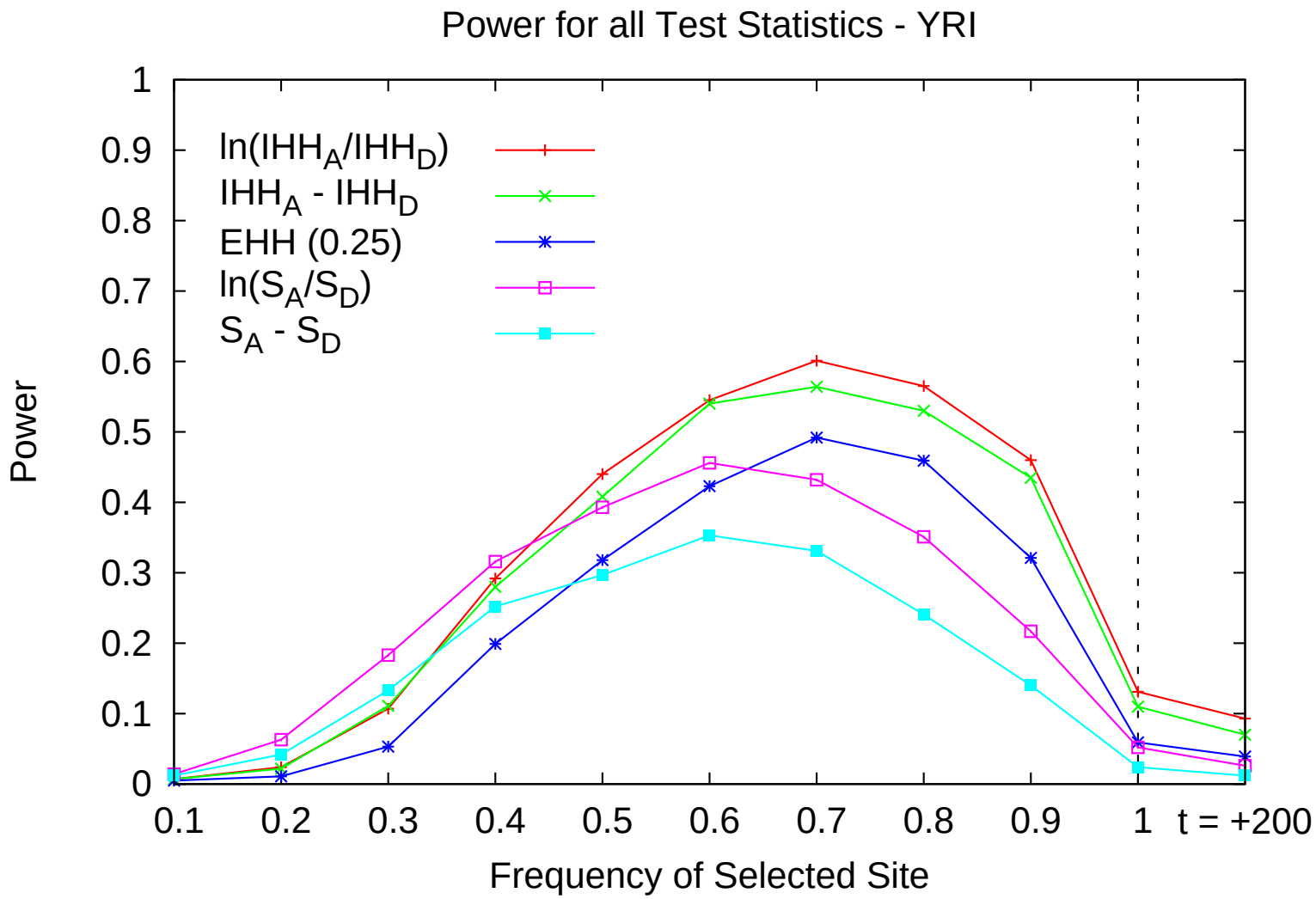

Figure S1: **Power to detect selection using various test statistics.** IHH_A and IHH_D refer to the integrals of the EHH curves for the ancestral and derived alleles respectively. Unlike those, S_A and S_D integrate over the frequency of the most common haplotype in the sample (rather than over EHH). We examined the difference or the log ratio of these measures, each standardized. "EHH $= 0.25$" is a statistic which, after standardization, is based on the log ratio of genetic distances measured at the single position where the EHH decays below 0.25, for the ancestral alleles over the derived allele, respectively.
