# Supplementary material for: A Map of Recent Positive Selection in the Human Genome
Source: PLoS Biol. 2006 Mar 7;4(3):e72. doi: 10.1371/journal.pbio.0040072 (PMC1382018; doi:10.1371/journal.pbio.0040072)
Supplement: Supplementary file 2 [file pbio.0040072.sg002.pdf]

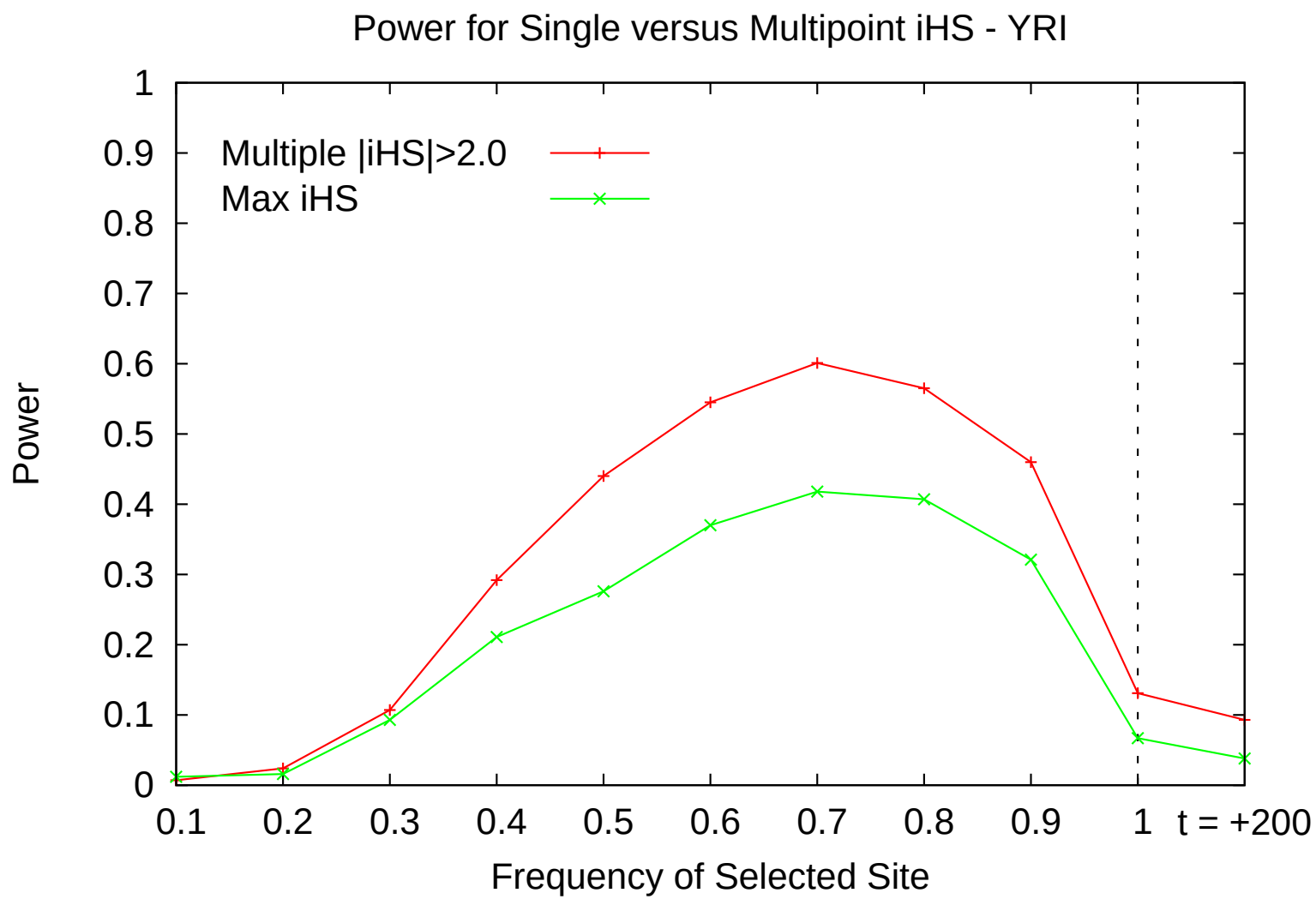

Figure S2: **Single vs multi-point iHS power plot.** Power to detect selection using iHS numerous extreme scores in a 50-SNP window versus the single most significant SNP (p=0.01; $\sigma = 150$)
