# Supplementary material for: A Map of Recent Positive Selection in the Human Genome
Source: PLoS Biol. 2006 Mar 7;4(3):e72. doi: 10.1371/journal.pbio.0040072 (PMC1382018; doi:10.1371/journal.pbio.0040072)
Supplement: Supplementary file 13 [file pbio.0040072.sg013.pdf]

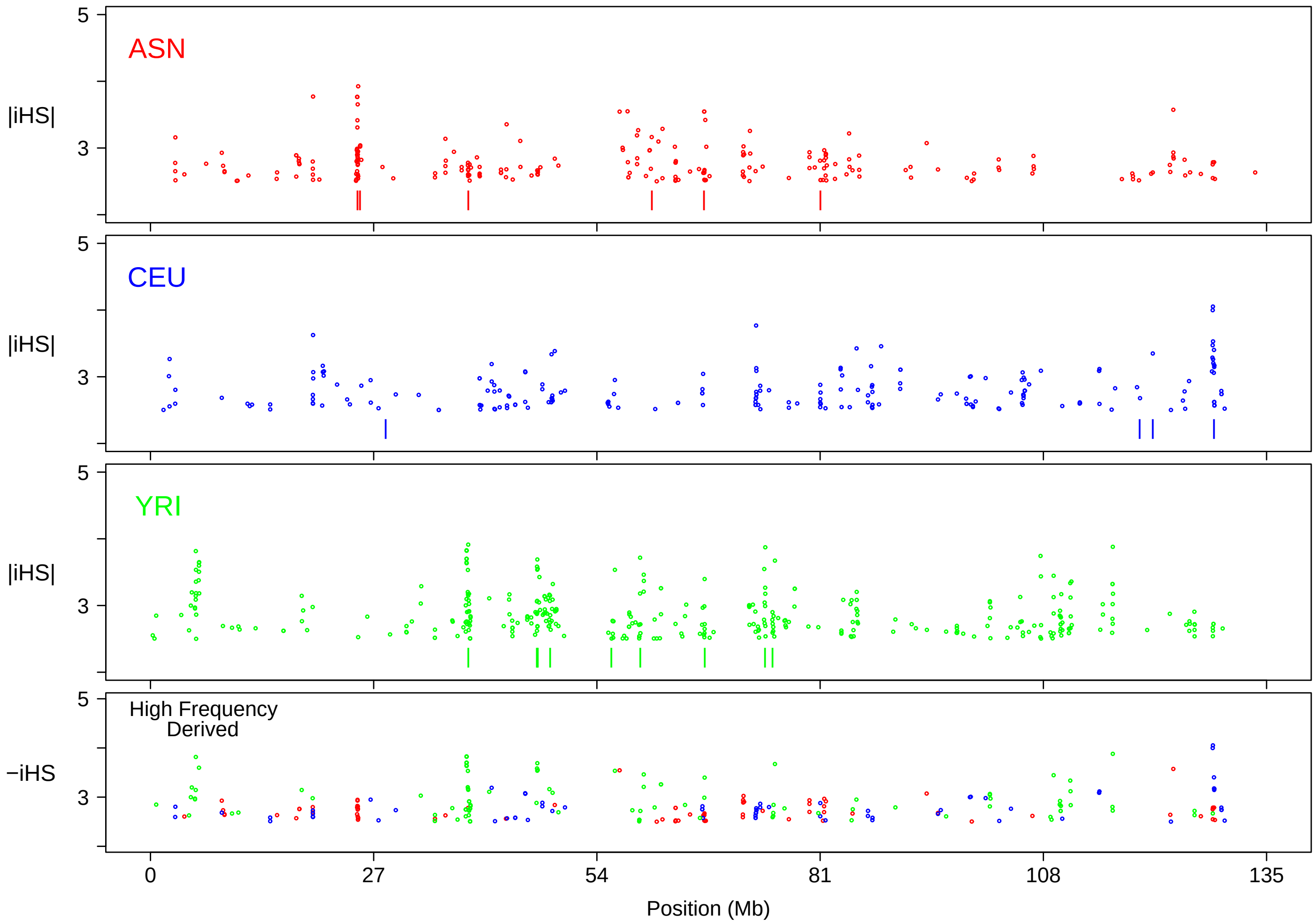


Figure S13: **Plots of Chromosome 11 SNPs with extreme iHS values illustrate discrete clusters of signals.** SNPs with $|\text{iHS}| > 2.5$ (top 1%) are plotted. The bottom plot combines signals for all three populations, plotting only SNPs with derived frequency $> 0.5$ and iHS $< -2.5$. Such SNPs correspond to high frequency derived SNPs in the range for which our test is most powerful. The short vertical bars below each plot indicate 100Kb windows whose signals are in the top 1% of windows genome-wide.
