# Supplementary material for: A Map of Recent Positive Selection in the Human Genome
Source: PLoS Biol. 2006 Mar 7;4(3):e72. doi: 10.1371/journal.pbio.0040072 (PMC1382018; doi:10.1371/journal.pbio.0040072)
Supplement: Supplementary file 26 [file pbio.0040072.sg026.pdf]

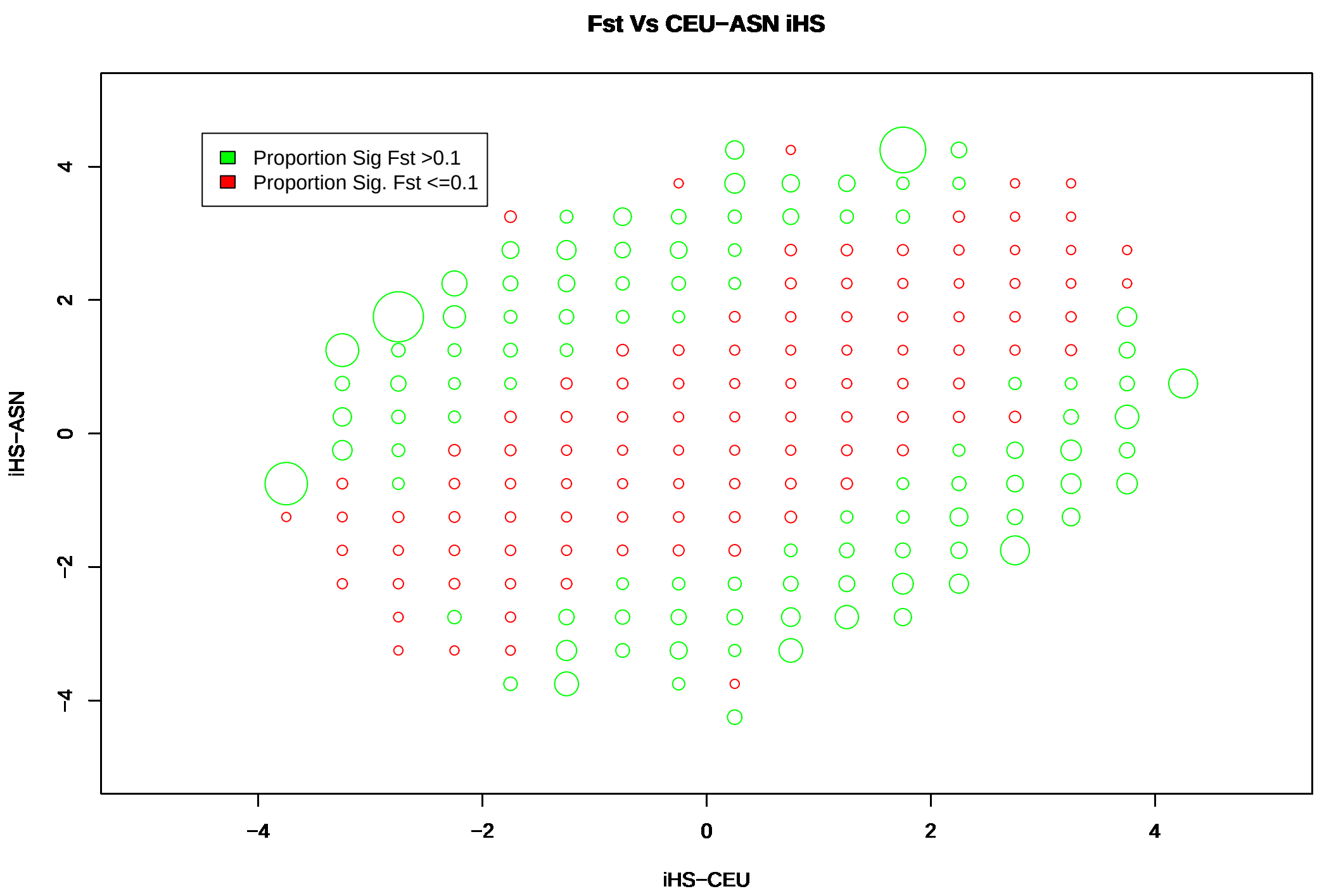

Figure S26: **Plot comparing Fst with iHS (CEU and ASN).** iHS in each population were grouped into bins of 0.5. The size of the circles in the plot represent the proportion of the SNPs with Fst greater than the genome wide empirical 5% threshold. Circles in red represent bins where the proportion of SNPs with Fst greater than the 5% threshold exceeds 10% and those in green represent bins where the proportion is less than 10%.
