# Supplementary material for: A Map of Recent Positive Selection in the Human Genome
Source: PLoS Biol. 2006 Mar 7;4(3):e72. doi: 10.1371/journal.pbio.0040072 (PMC1382018; doi:10.1371/journal.pbio.0040072)
Supplement: Supplementary file 30 [file pbio.0040072.sg030.pdf]

# Supplementary Material

A map of recent positive selection in the human genome

Benjamin F. Voight, Sridhar Kudaravalli,
Xiaoquan Wen, Jonathan K. Pritchard

Department of Human Genetics
University of Chicago

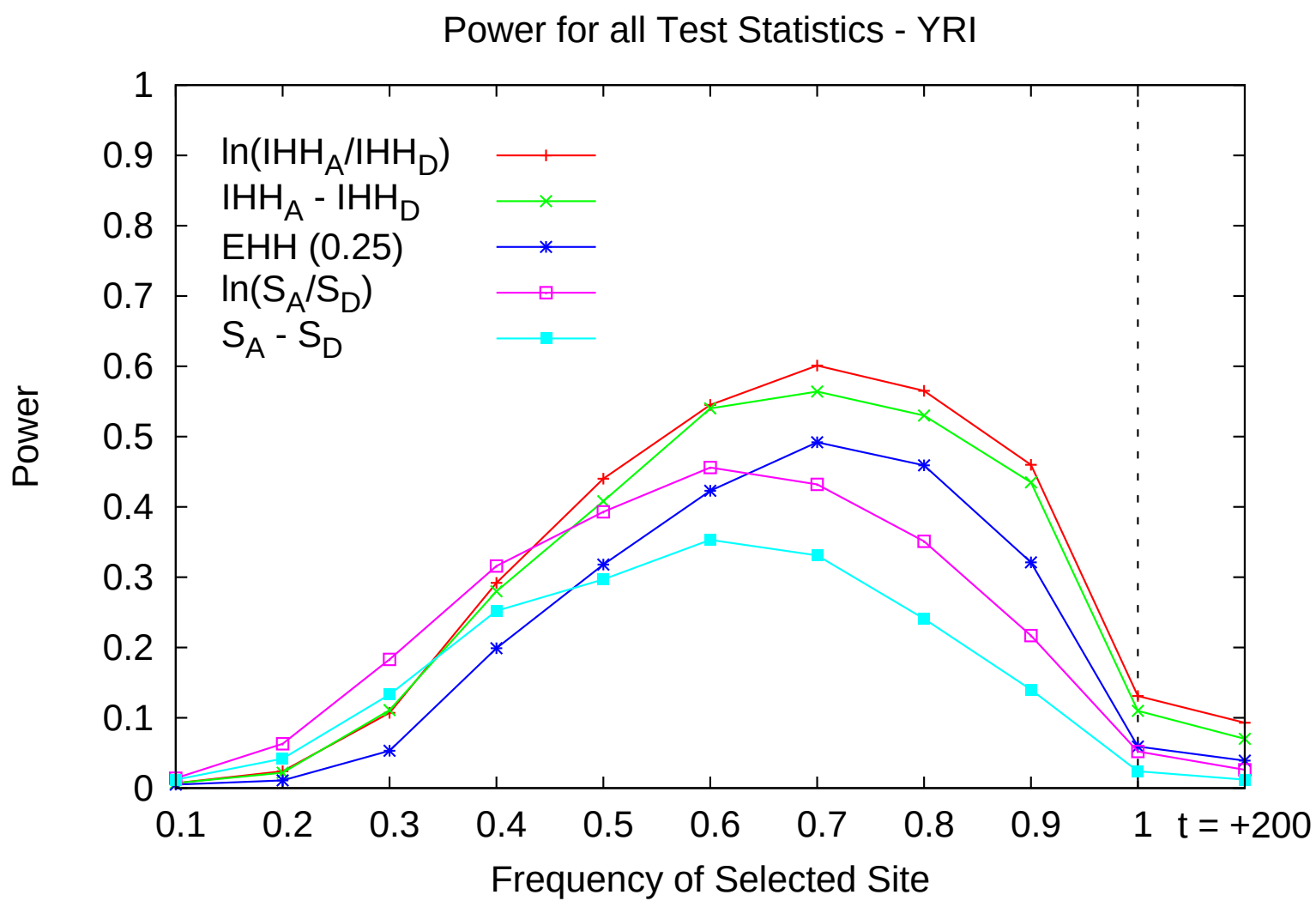


Figure S1: **Power to detect selection using various test statistics.** $\mathrm{IHH}_A$ and $\mathrm{IHH}_D$ refer to the integrals of the EHH curves for the ancestral and derived alleles respectively. Unlike those, $S_A$ and $S_D$ integrate over the frequency of the most common haplotype in the sample (rather than over EHH). We examined the difference or the log ratio of these measures, each standardized. "EHH $= 0.25$" is a statistic which, after standardization, is based on the log ratio of genetic distances measured at the single position where the EHH decays below 0.25, for the ancestral alleles over the derived allele, respectively.

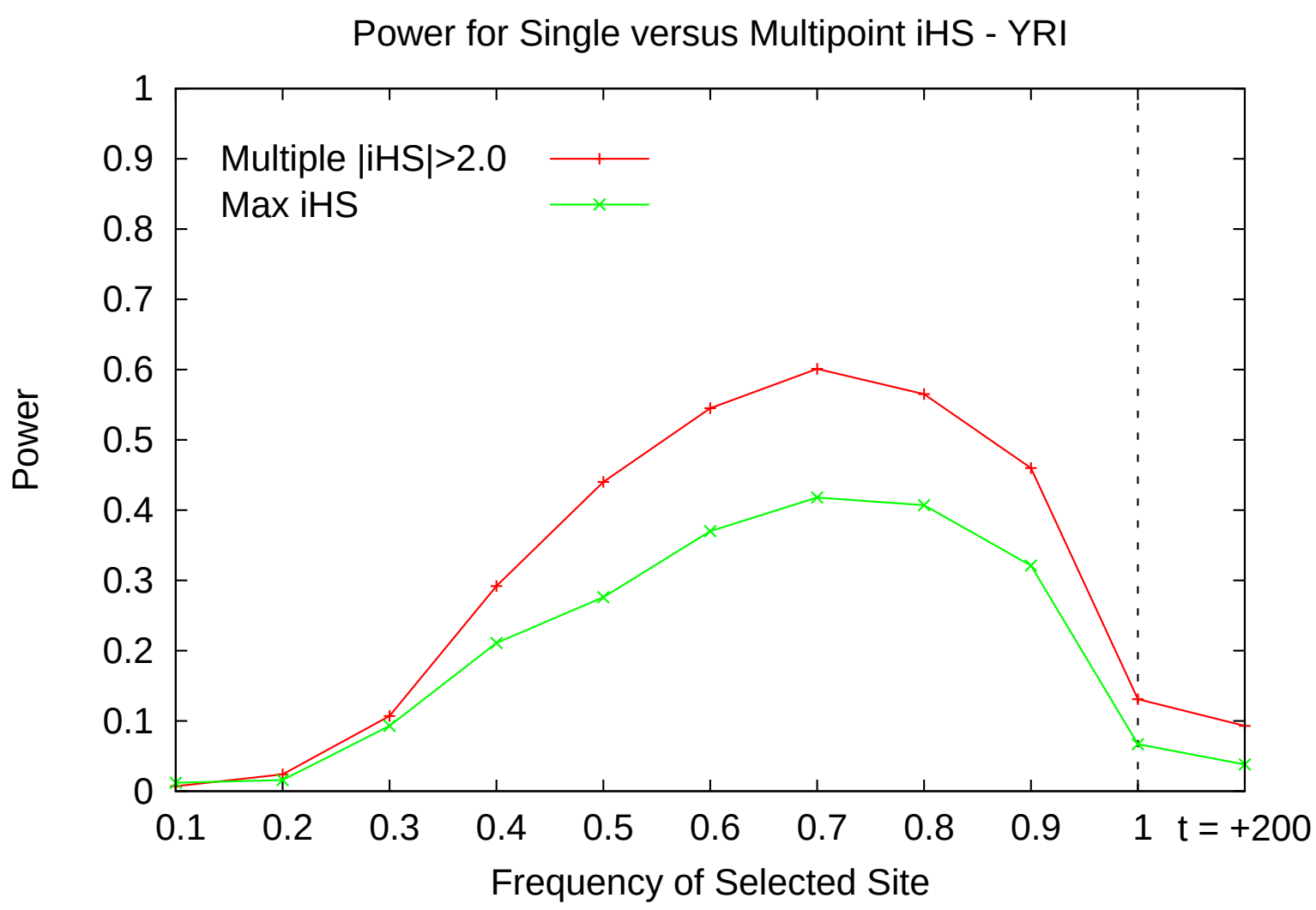


Figure S2: **Single vs multi-point iHS power plot.** Power to detect selection using iHS numerous extreme scores in a 50-SNP window versus the single most significant SNP (p=0.01; $\sigma = 150$)

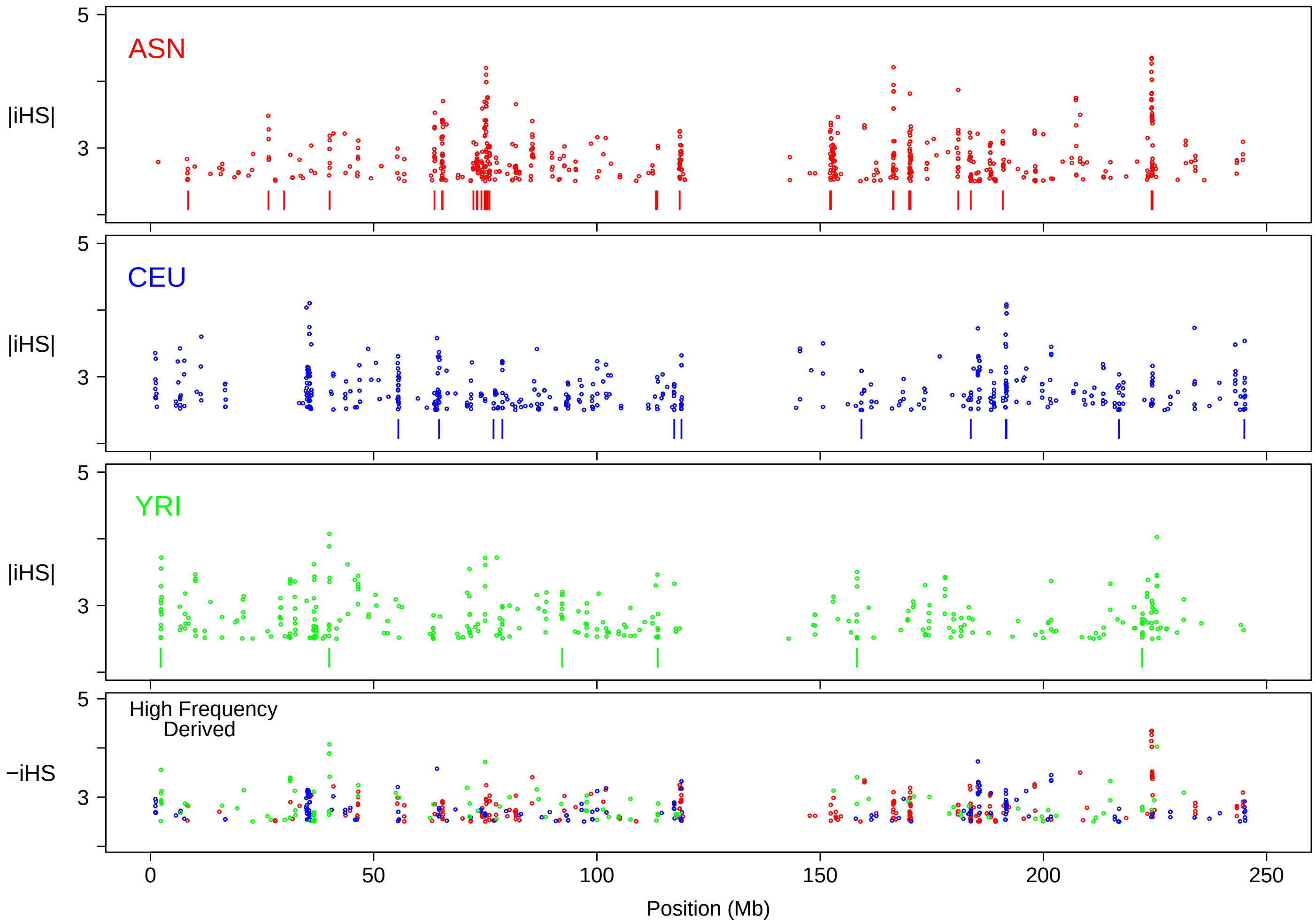


Figure S3: **Plots of Chromosome 1 SNPs with extreme iHS values illustrate discrete clusters of signals.** SNPs with |iHS| $> 2.5$ (top 1%) are plotted. The bottom plot combines signals for all three populations, plotting only SNPs with derived frequency $> 0.5$ and iHS $< -2.5$. Such SNPs correspond to high frequency derived SNPs in the range for which our test is most powerful. The short vertical bars below each plot indicate 100Kb windows whose signals are in the top 1% of windows genome-wide.

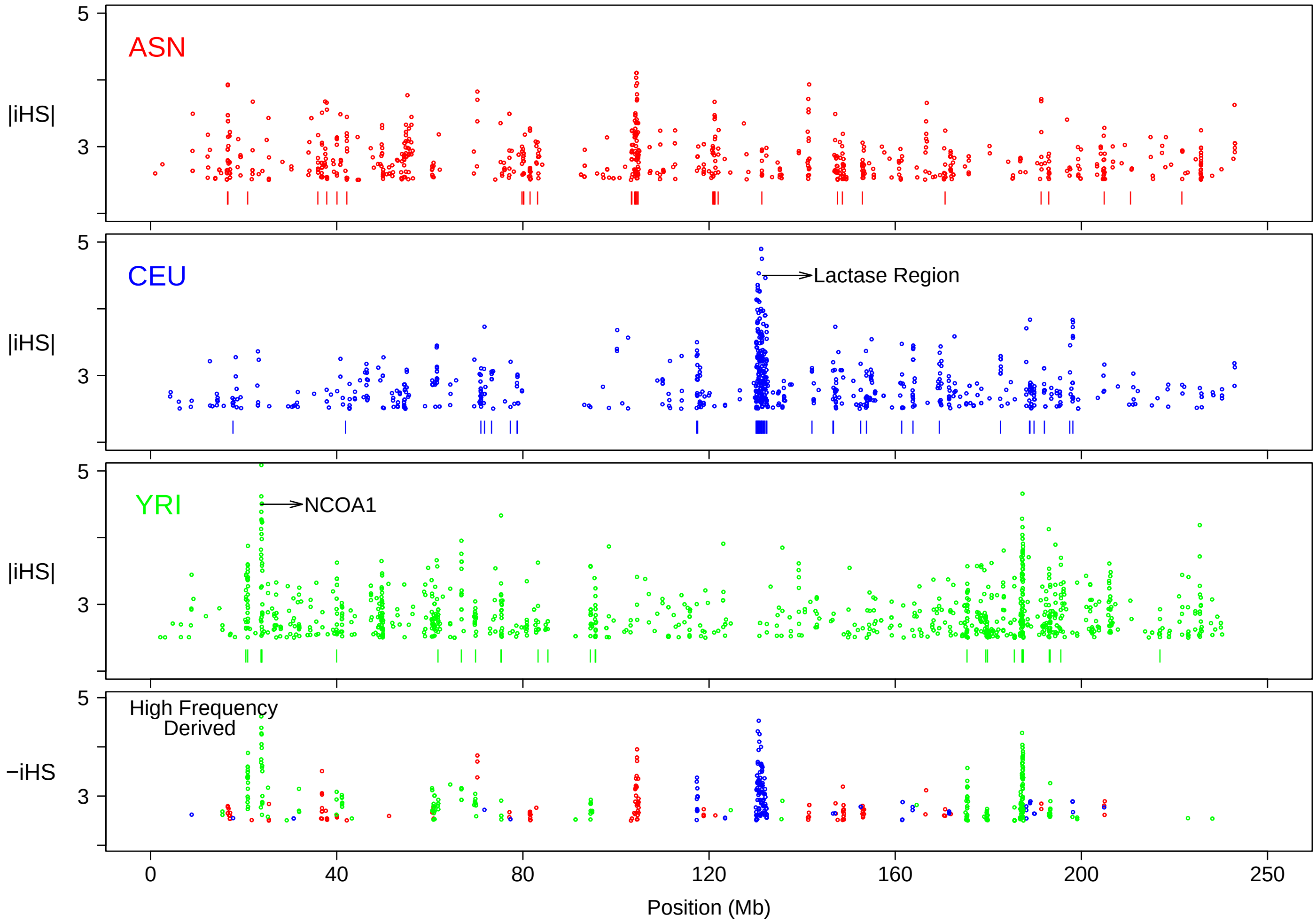


Figure S4: **Plots of Chromosome 2 SNPs with extreme iHS values illustrate discrete clusters of signals.** SNPs with $|\text{iHS}| > 2.5$ (top 1%) are plotted. The bottom plot combines signals for all three populations, plotting only SNPs with derived frequency $> 0.5$ and iHS $< -2.5$. Such SNPs correspond to high frequency derived SNPs in the range for which our test is most powerful. The short vertical bars below each plot indicate 100Kb windows whose signals are in the top 1% of windows genome-wide.

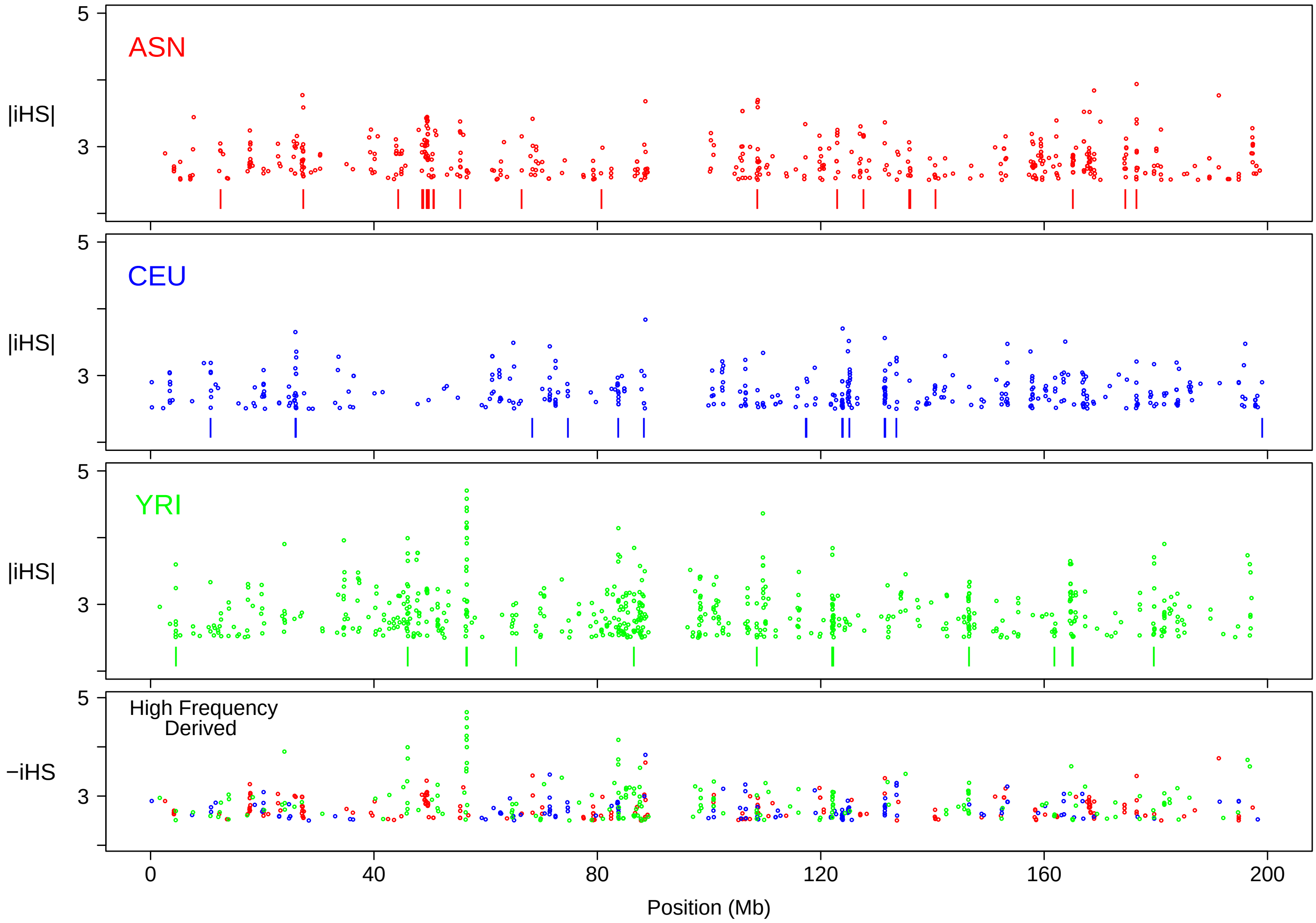


Figure S5: **Plots of Chromosome 3 SNPs with extreme iHS values illustrate discrete clusters of signals.** SNPs with |iHS| > 2.5 (top 1%) are plotted. The bottom plot combines signals for all three populations, plotting only SNPs with derived frequency > 0.5 and iHS < −2.5. Such SNPs correspond to high frequency derived SNPs in the range for which our test is most powerful. The short vertical bars below each plot indicate 100Kb windows whose signals are in the top 1% of windows genome-wide.

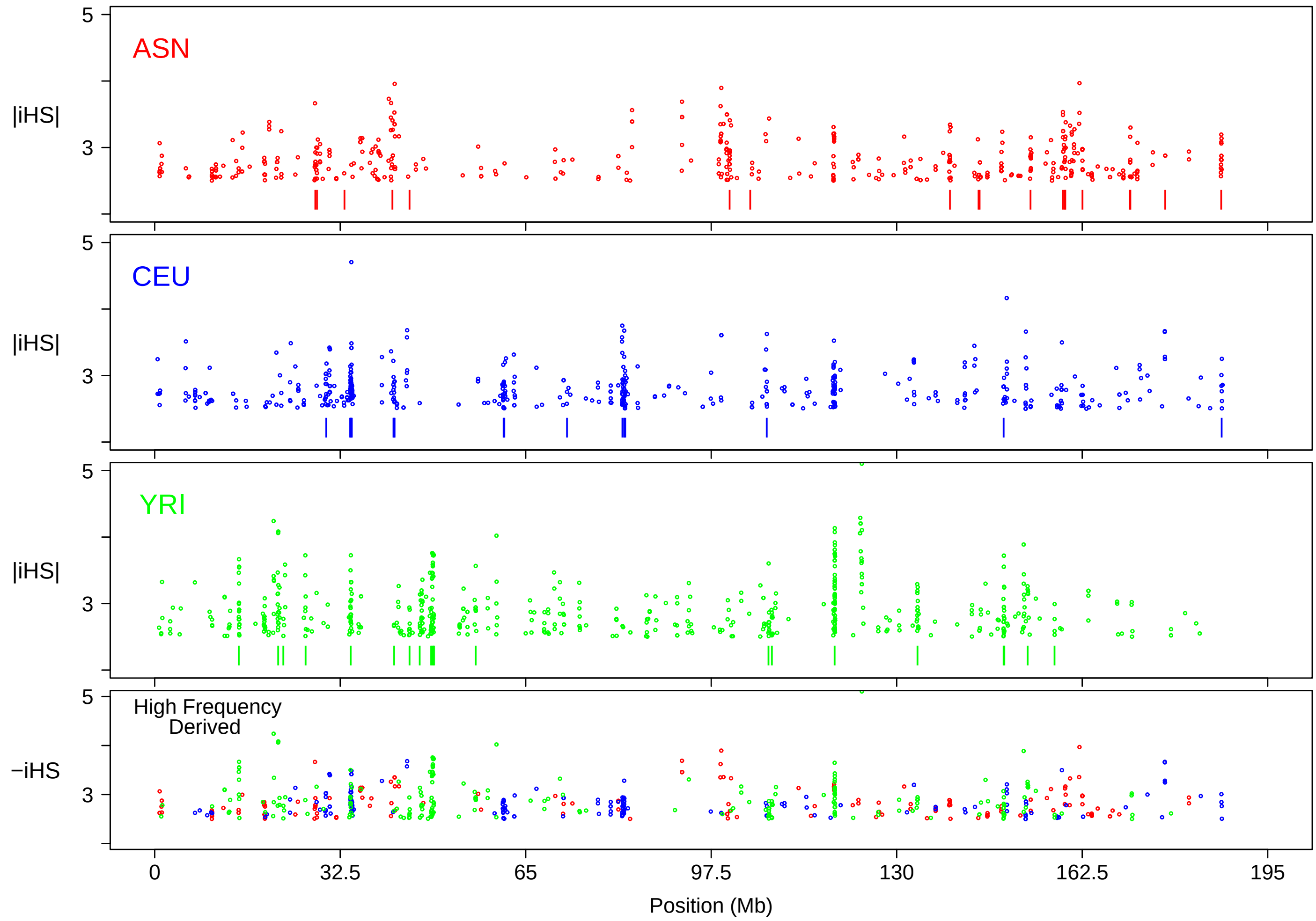


Figure S6: **Plots of Chromosome 4 SNPs with extreme iHS values illustrate discrete clusters of signals.** SNPs with $|\text{iHS}| > 2.5$ (top 1%) are plotted. The bottom plot combines signals for all three populations, plotting only SNPs with derived frequency $> 0.5$ and iHS $< -2.5$. Such SNPs correspond to high frequency derived SNPs in the range for which our test is most powerful. The short vertical bars below each plot indicate 100Kb windows whose signals are in the top 1% of windows genome-wide.

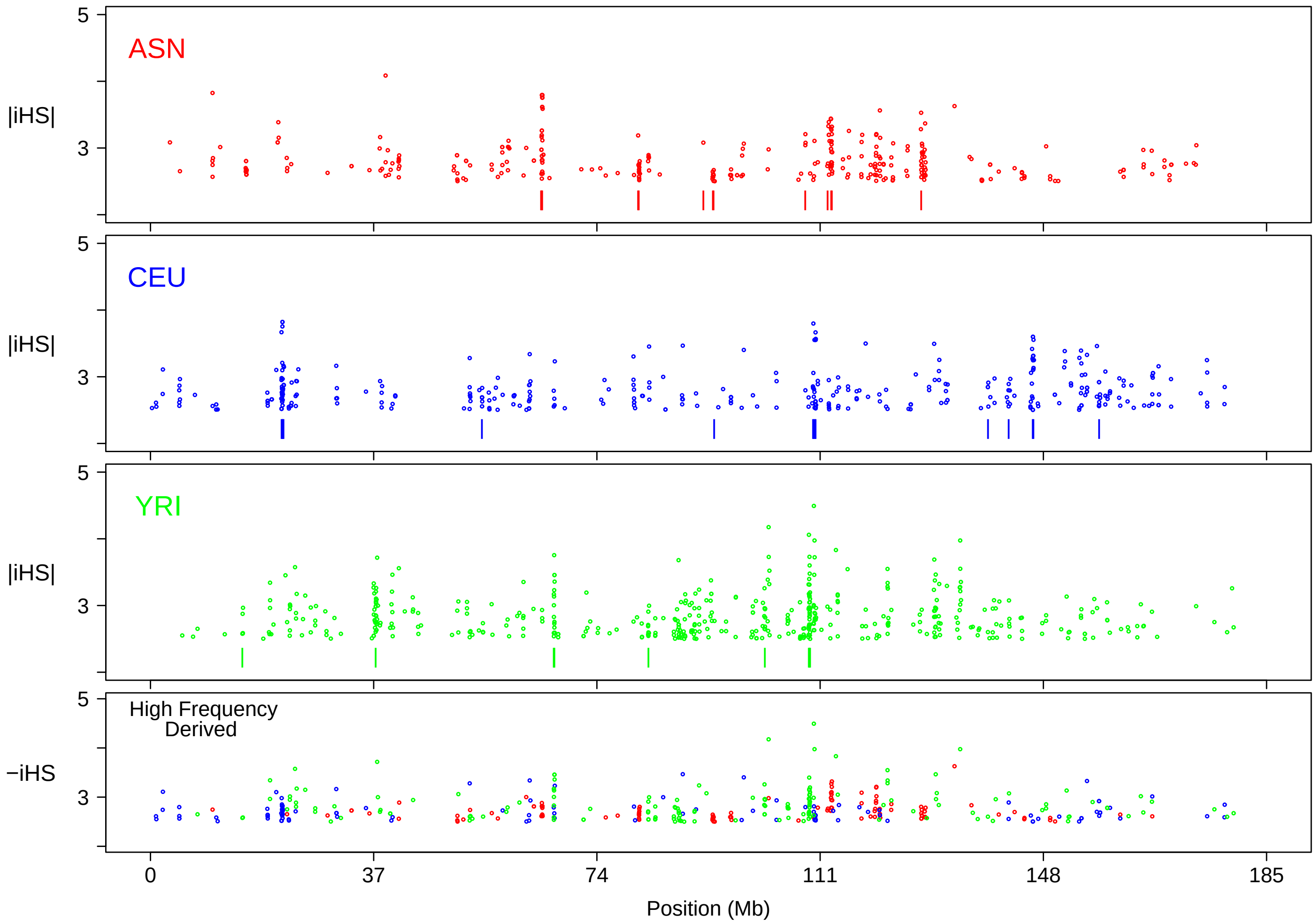


Figure S7: **Plots of Chromosome 5 SNPs with extreme iHS values illustrate discrete clusters of signals.** SNPs with $|iHS| > 2.5$ (top 1%) are plotted. The bottom plot combines signals for all three populations, plotting only SNPs with derived frequency $> 0.5$ and iHS $< -2.5$. Such SNPs correspond to high frequency derived SNPs in the range for which our test is most powerful. The short vertical bars below each plot indicate 100Kb windows whose signals are in the top 1% of windows genome-wide.

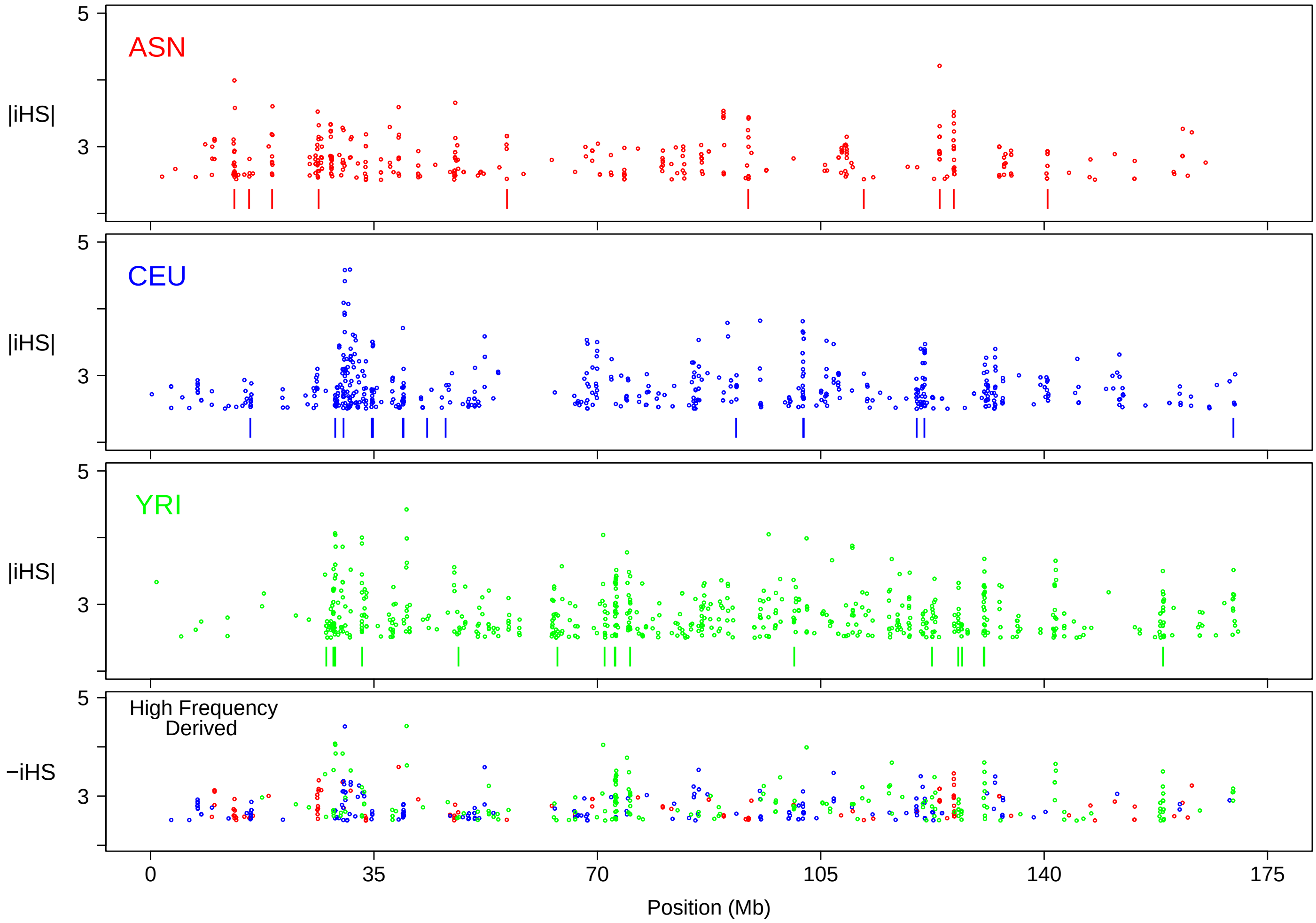


Figure S8: **Plots of Chromosome 6 SNPs with extreme iHS values illustrate discrete clusters of signals.** SNPs with |iHS| > 2.5 (top 1%) are plotted. The bottom plot combines signals for all three populations, plotting only SNPs with derived frequency > 0.5 and iHS < −2.5. Such SNPs correspond to high frequency derived SNPs in the range for which our test is most powerful. The short vertical bars below each plot indicate 100Kb windows whose signals are in the top 1% of windows genome-wide.

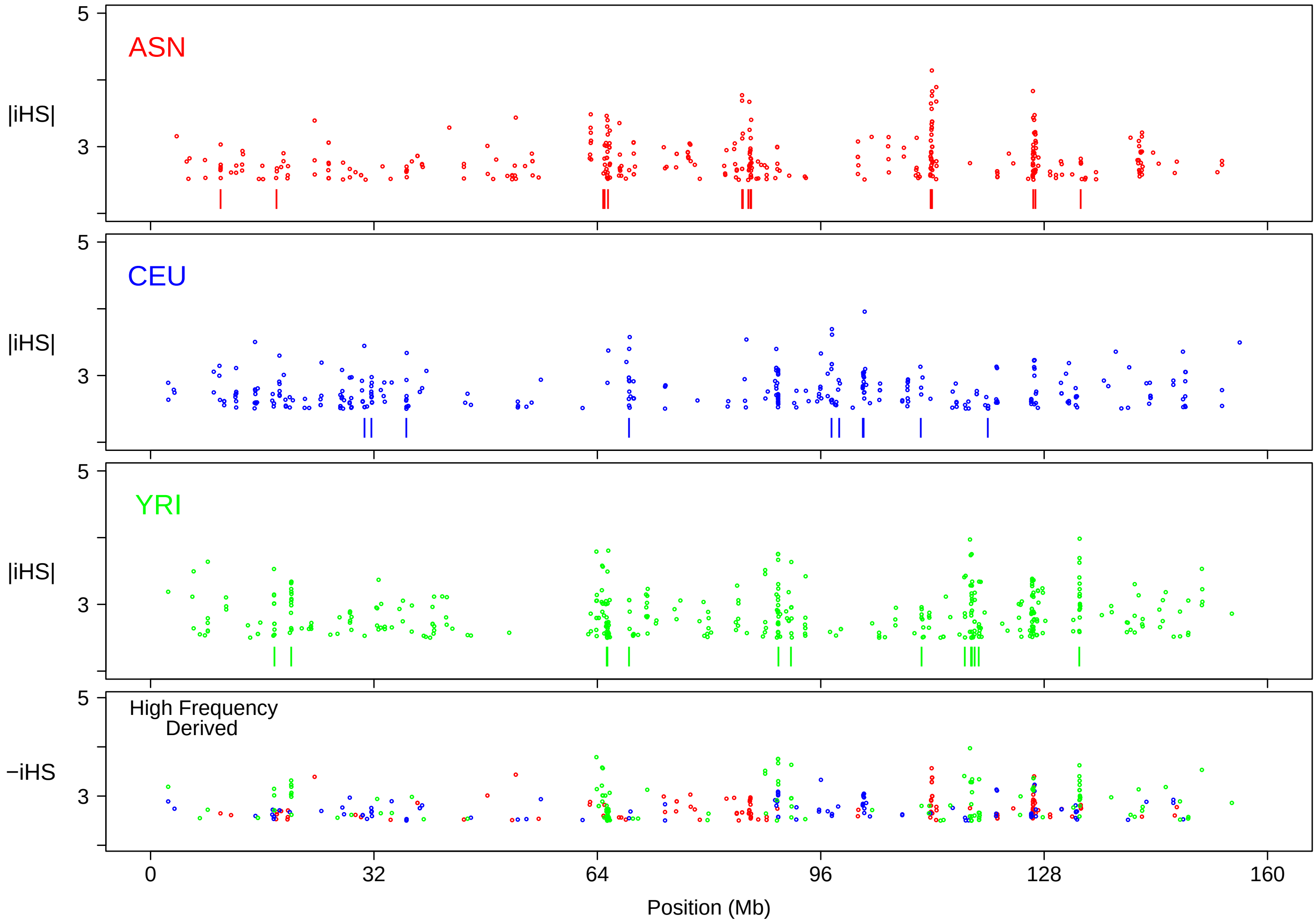


Figure S9: **Plots of Chromosome 7 SNPs with extreme iHS values illustrate discrete clusters of signals.** SNPs with $|\text{iHS}| > 2.5$ (top 1%) are plotted. The bottom plot combines signals for all three populations, plotting only SNPs with derived frequency $> 0.5$ and iHS $< -2.5$. Such SNPs correspond to high frequency derived SNPs in the range for which our test is most powerful. The short vertical bars below each plot indicate 100Kb windows whose signals are in the top 1% of windows genome-wide.

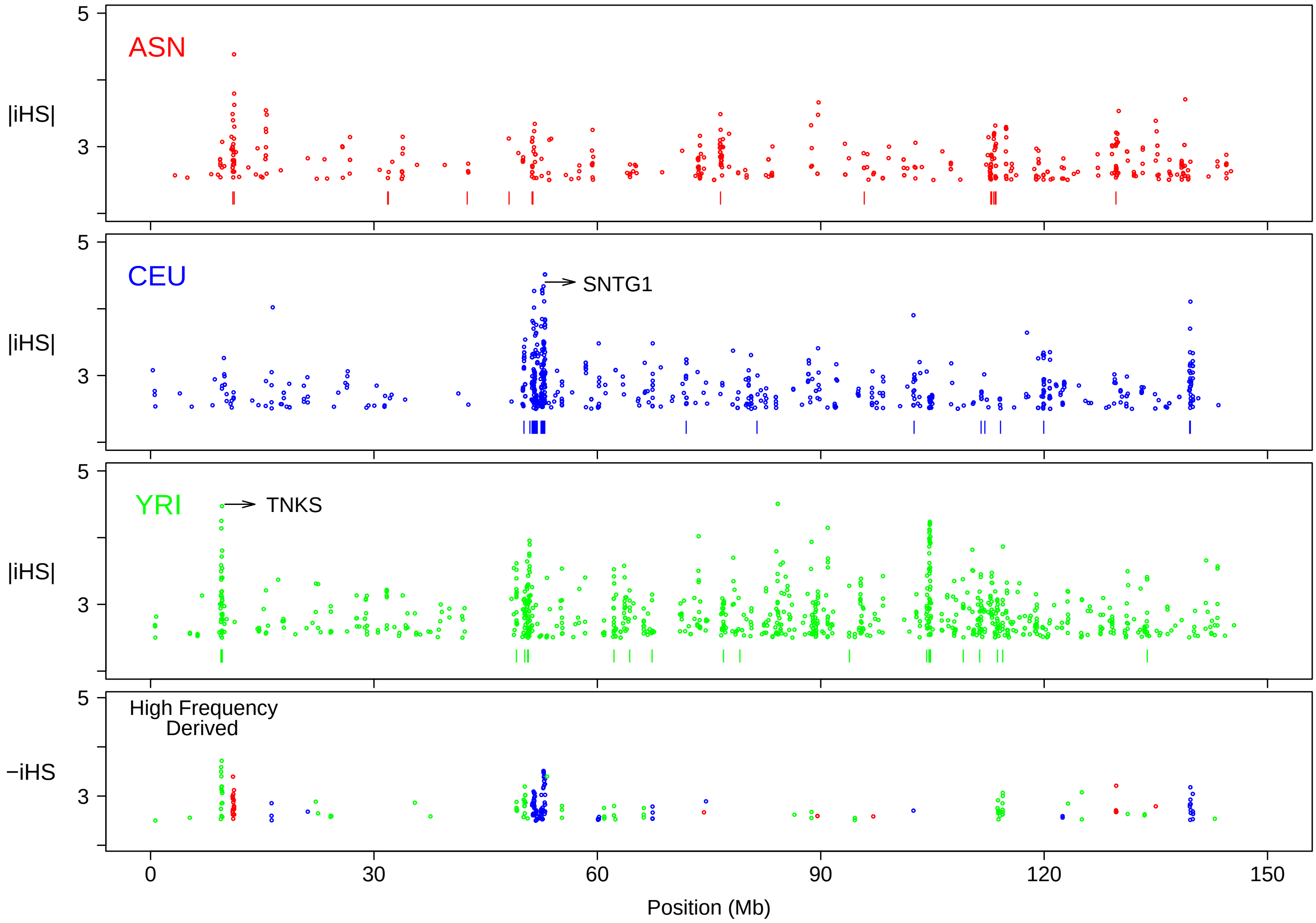


Figure S10: **Plots of Chromosome 8 SNPs with extreme iHS values illustrate discrete clusters of signals.** SNPs with |iHS| $> 2.5$ (top 1%) are plotted. The bottom plot combines signals for all three populations, plotting only SNPs with derived frequency $> 0.5$ and iHS $< -2.5$. Such SNPs correspond to high frequency derived SNPs in the range for which our test is most powerful. The short vertical bars below each plot indicate 100Kb windows whose signals are in the top 1% of windows genome-wide.

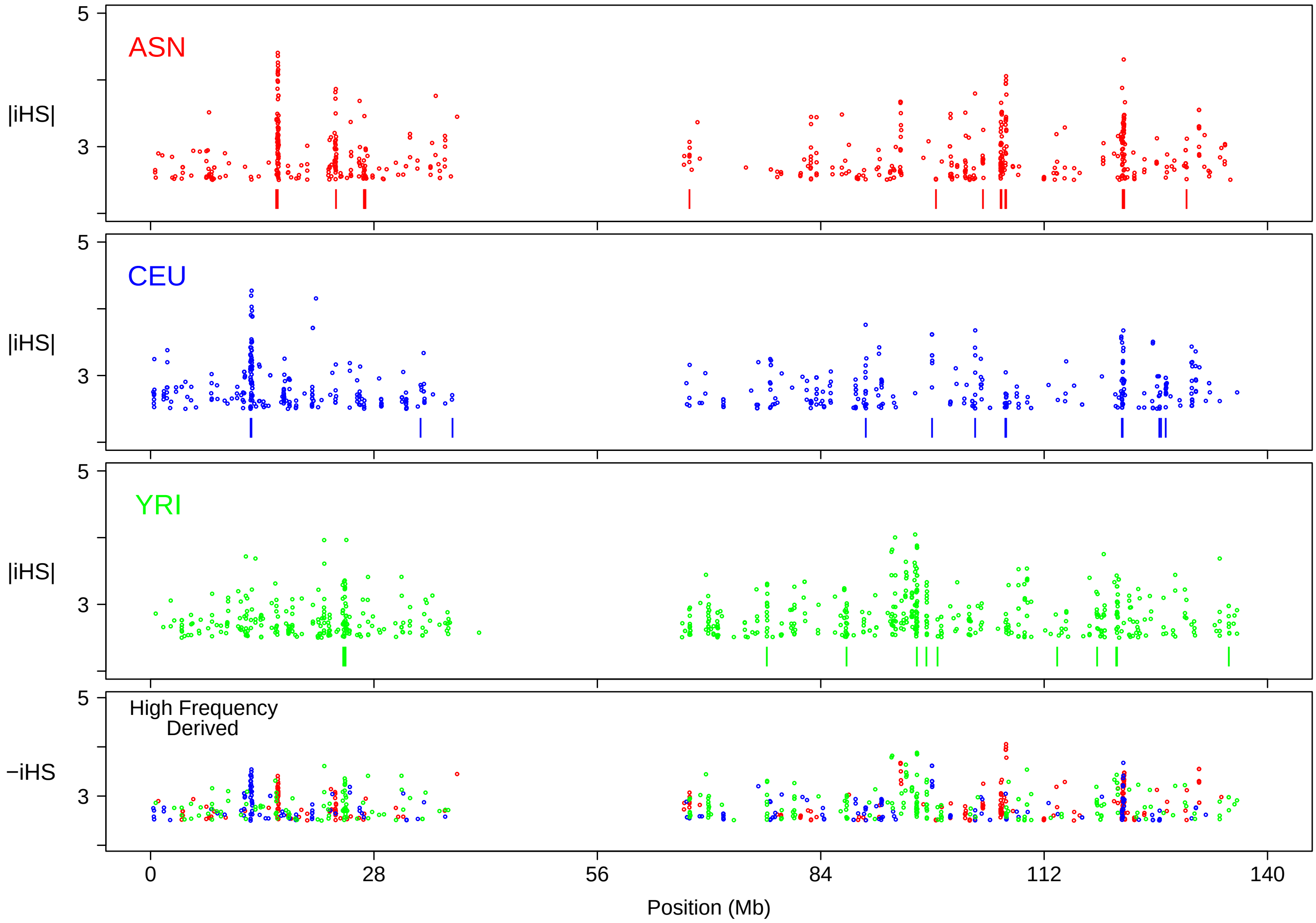


Figure S11: **Plots of Chromosome 9 SNPs with extreme iHS values illustrate discrete clusters of signals.** SNPs with |iHS| > 2.5 (top 1%) are plotted. The bottom plot combines signals for all three populations, plotting only SNPs with derived frequency > 0.5 and iHS < −2.5. Such SNPs correspond to high frequency derived SNPs in the range for which our test is most powerful. The short vertical bars below each plot indicate 100Kb windows whose signals are in the top 1% of windows genome-wide.

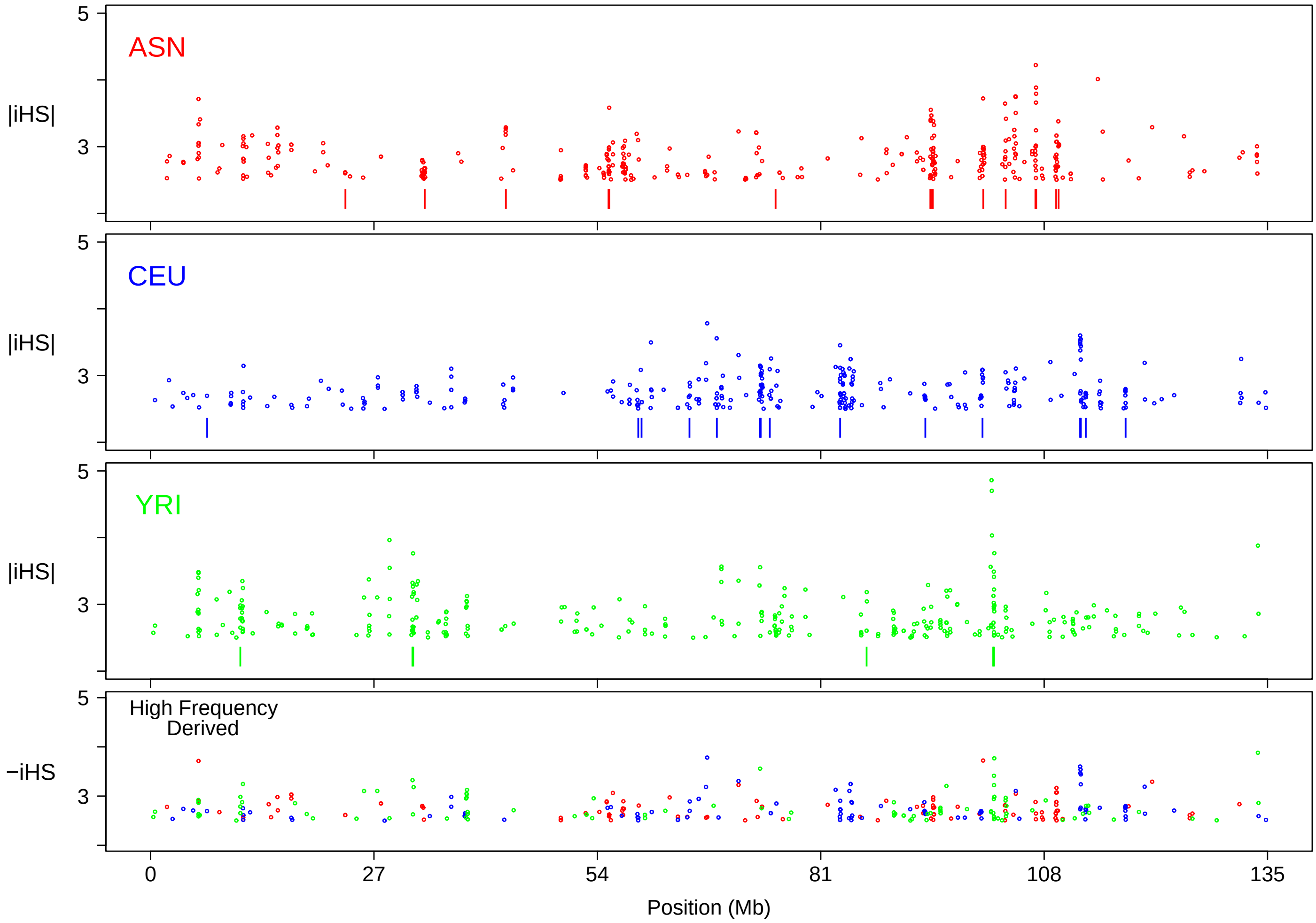


Figure S12: **Plots of Chromosome 10 SNPs with extreme iHS values illustrate discrete clusters of signals.** SNPs with |iHS| > 2.5 (top 1%) are plotted. The bottom plot combines signals for all three populations, plotting only SNPs with derived frequency > 0.5 and iHS < −2.5. Such SNPs correspond to high frequency derived SNPs in the range for which our test is most powerful. The short vertical bars below each plot indicate 100Kb windows whose signals are in the top 1% of windows genome-wide.

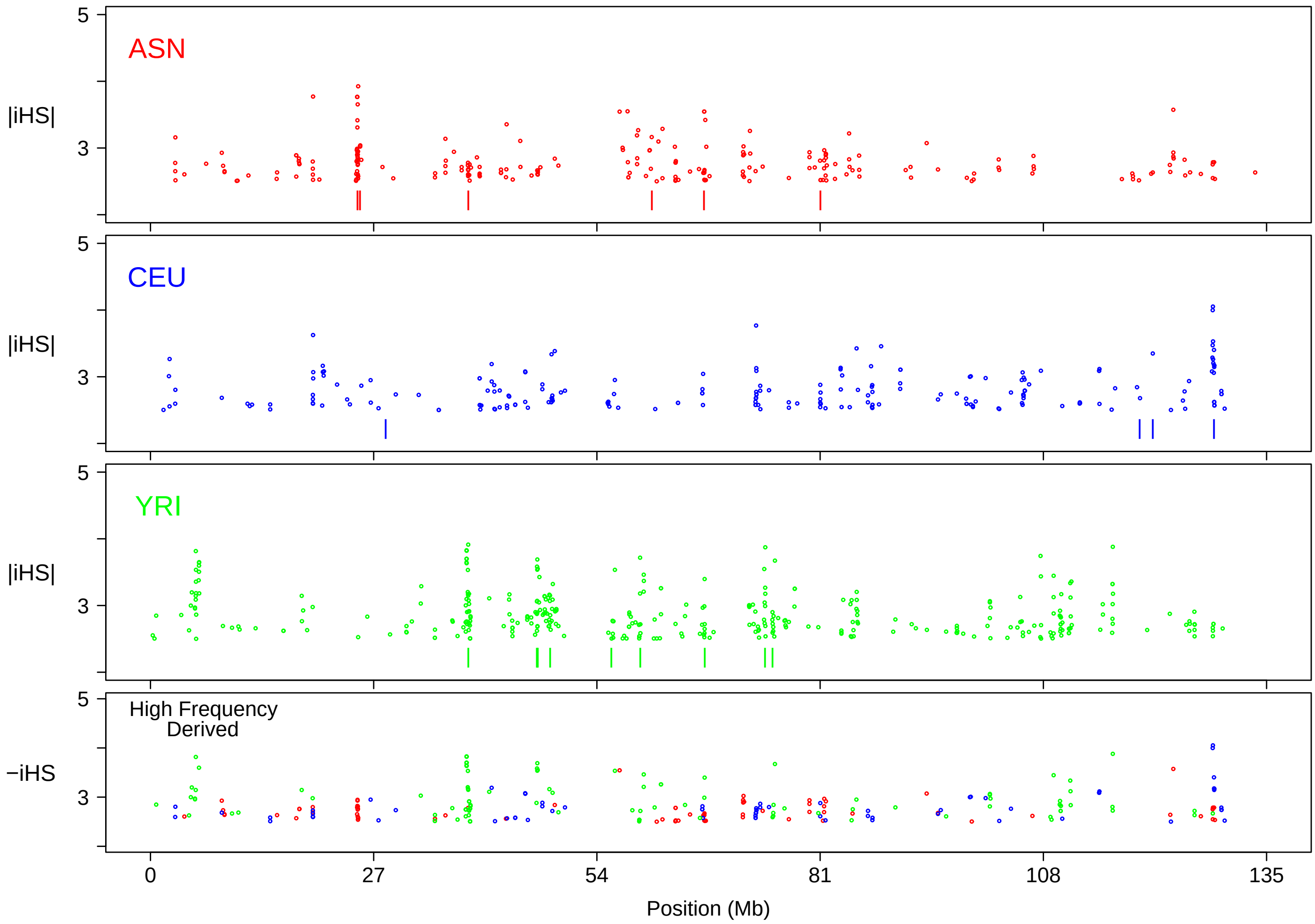


Figure S13: **Plots of Chromosome 11 SNPs with extreme iHS values illustrate discrete clusters of signals.** SNPs with $|\text{iHS}| > 2.5$ (top 1%) are plotted. The bottom plot combines signals for all three populations, plotting only SNPs with derived frequency $> 0.5$ and $\text{iHS} < -2.5$. Such SNPs correspond to high frequency derived SNPs in the range for which our test is most powerful. The short vertical bars below each plot indicate 100Kb windows whose signals are in the top 1% of windows genome-wide.

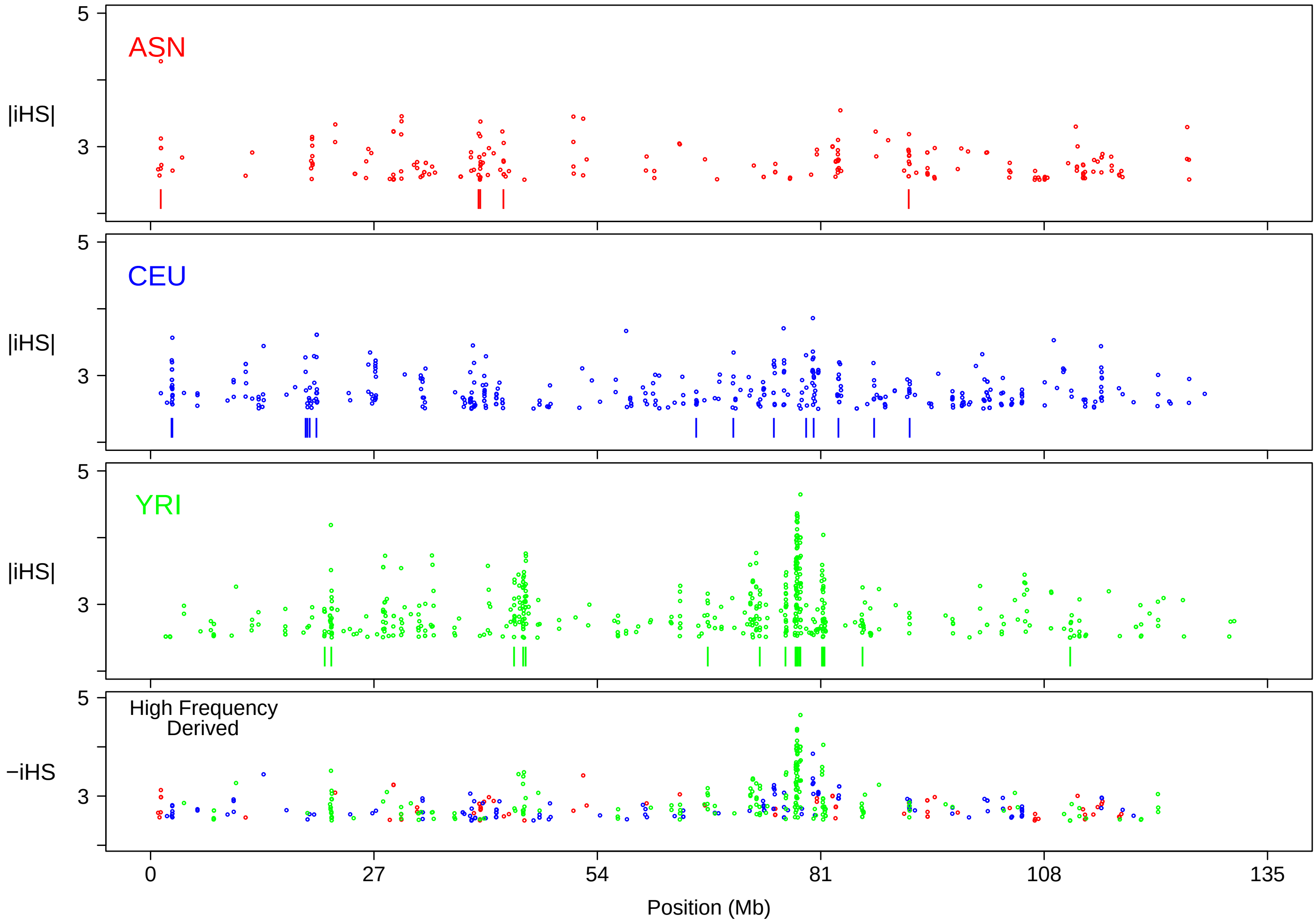


Figure S14: **Plots of Chromosome 12 SNPs with extreme iHS values illustrate discrete clusters of signals.** SNPs with $|iHS| > 2.5$ (top 1%) are plotted. The bottom plot combines signals for all three populations, plotting only SNPs with derived frequency $> 0.5$ and $iHS < -2.5$. Such SNPs correspond to high frequency derived SNPs in the range for which our test is most powerful. The short vertical bars below each plot indicate 100Kb windows whose signals are in the top 1% of windows genome-wide.

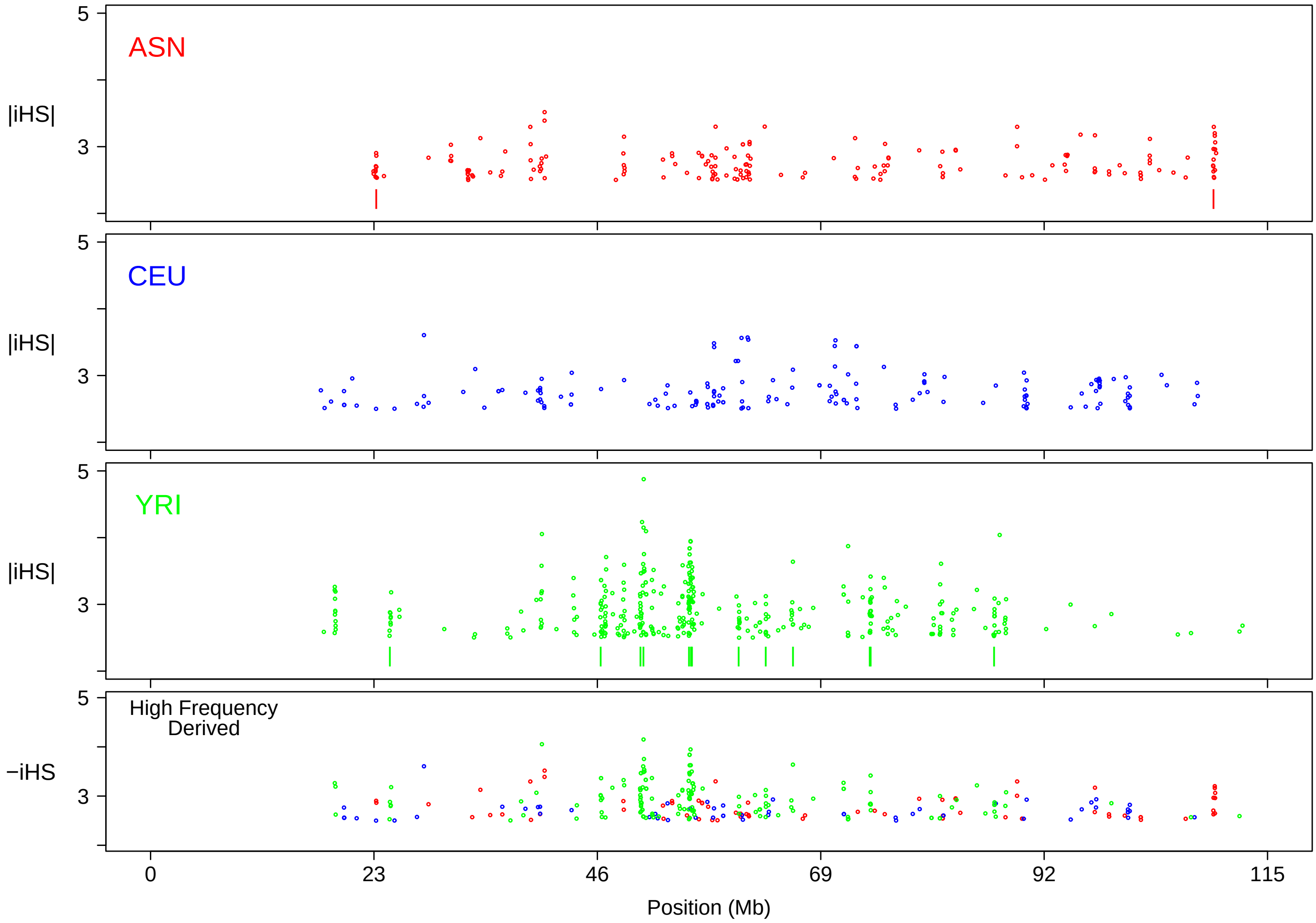


Figure S15: **Plots of Chromosome 13 SNPs with extreme iHS values illustrate discrete clusters of signals.** SNPs with $|\text{iHS}| > 2.5$ (top 1%) are plotted. The bottom plot combines signals for all three populations, plotting only SNPs with derived frequency $> 0.5$ and iHS $< -2.5$. Such SNPs correspond to high frequency derived SNPs in the range for which our test is most powerful. The short vertical bars below each plot indicate 100Kb windows whose signals are in the top 1% of windows genome-wide.

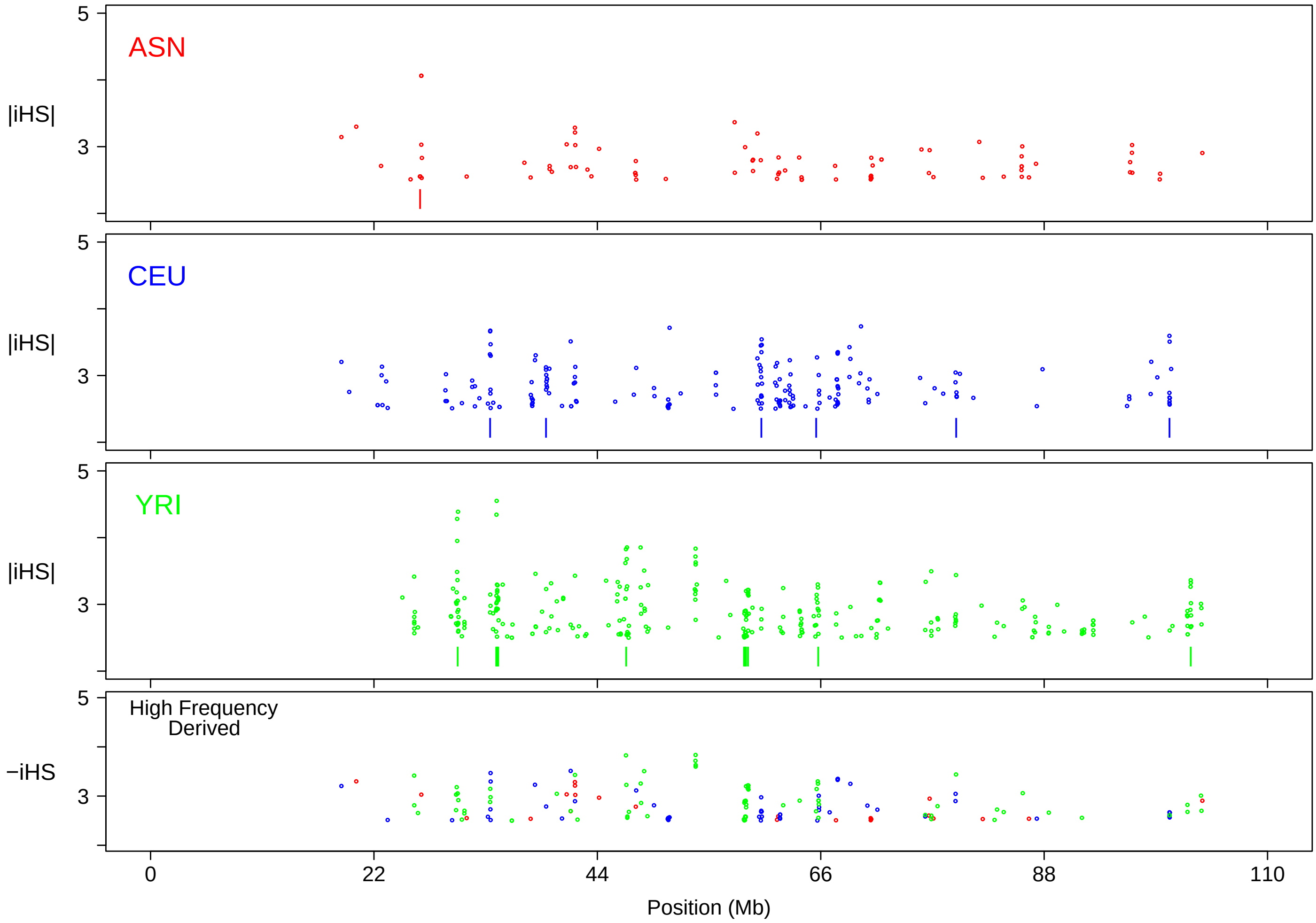


Figure S16: **Plots of Chromosome 14 SNPs with extreme iHS values illustrate discrete clusters of signals.** SNPs with |iHS| $> 2.5$ (top 1%) are plotted. The bottom plot combines signals for all three populations, plotting only SNPs with derived frequency $> 0.5$ and iHS $< -2.5$. Such SNPs correspond to high frequency derived SNPs in the range for which our test is most powerful. The short vertical bars below each plot indicate 100Kb windows whose signals are in the top 1% of windows genome-wide.

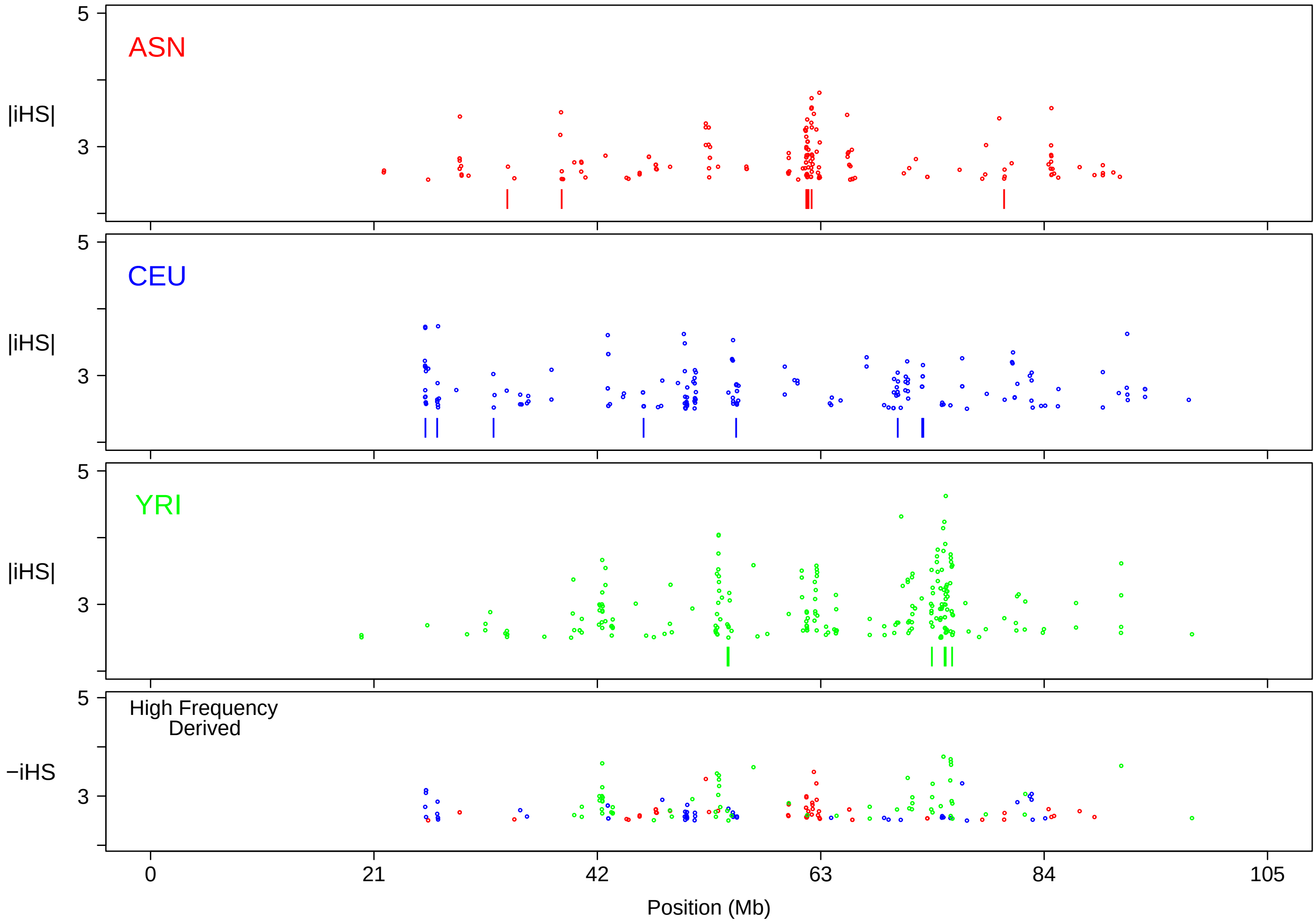


Figure S17: **Plots of Chromosome 15 SNPs with extreme iHS values illustrate discrete clusters of signals.** SNPs with $|\text{iHS}| > 2.5$ (top 1%) are plotted. The bottom plot combines signals for all three populations, plotting only SNPs with derived frequency $> 0.5$ and iHS $< -2.5$. Such SNPs correspond to high frequency derived SNPs in the range for which our test is most powerful. The short vertical bars below each plot indicate 100Kb windows whose signals are in the top 1% of windows genome-wide.

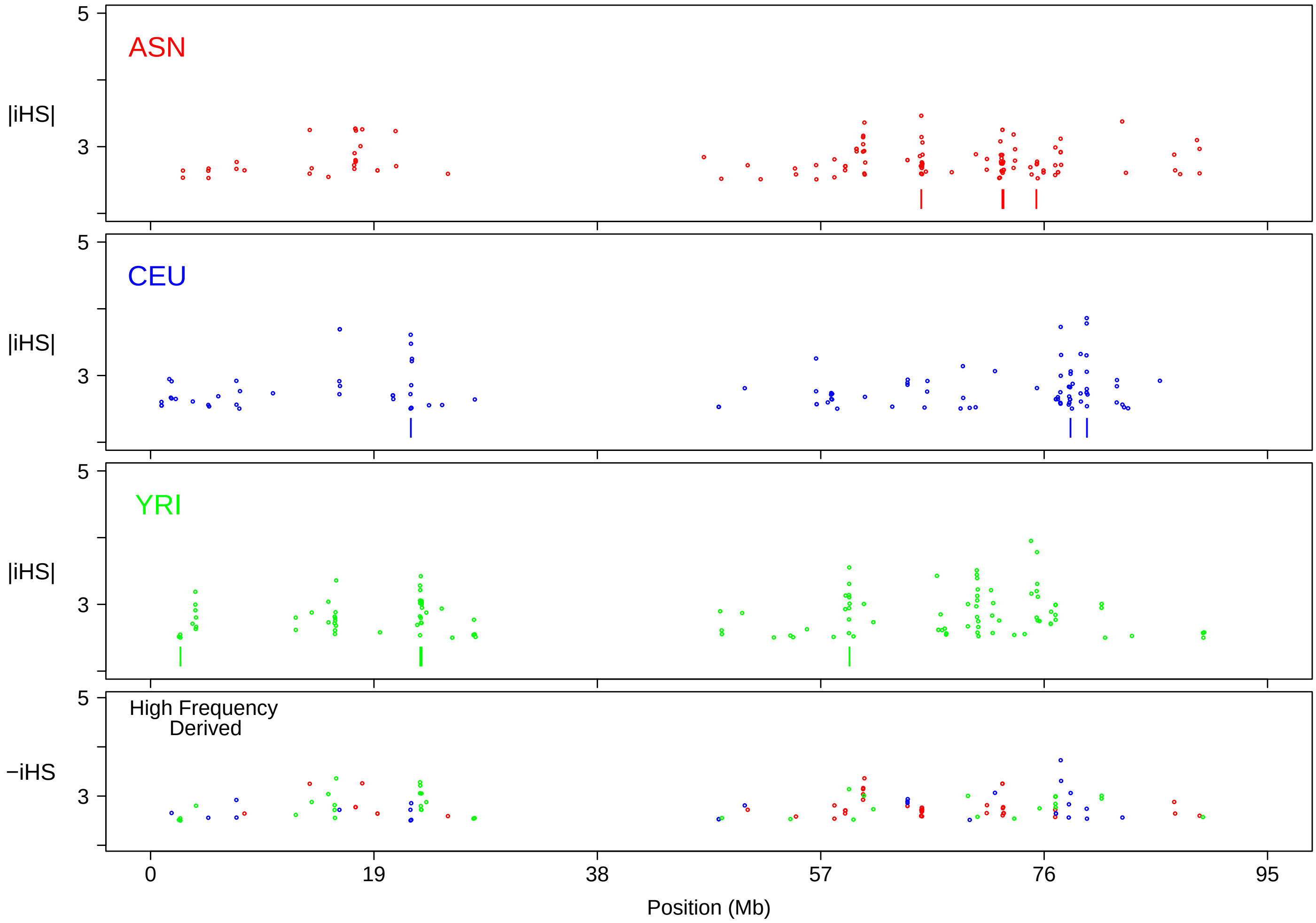


Figure S18: **Plots of Chromosome 16 SNPs with extreme iHS values illustrate discrete clusters of signals.** SNPs with |iHS| > 2.5 (top 1%) are plotted. The bottom plot combines signals for all three populations, plotting only SNPs with derived frequency > 0.5 and iHS < −2.5. Such SNPs correspond to high frequency derived SNPs in the range for which our test is most powerful. The short vertical bars below each plot indicate 100Kb windows whose signals are in the top 1% of windows genome-wide.

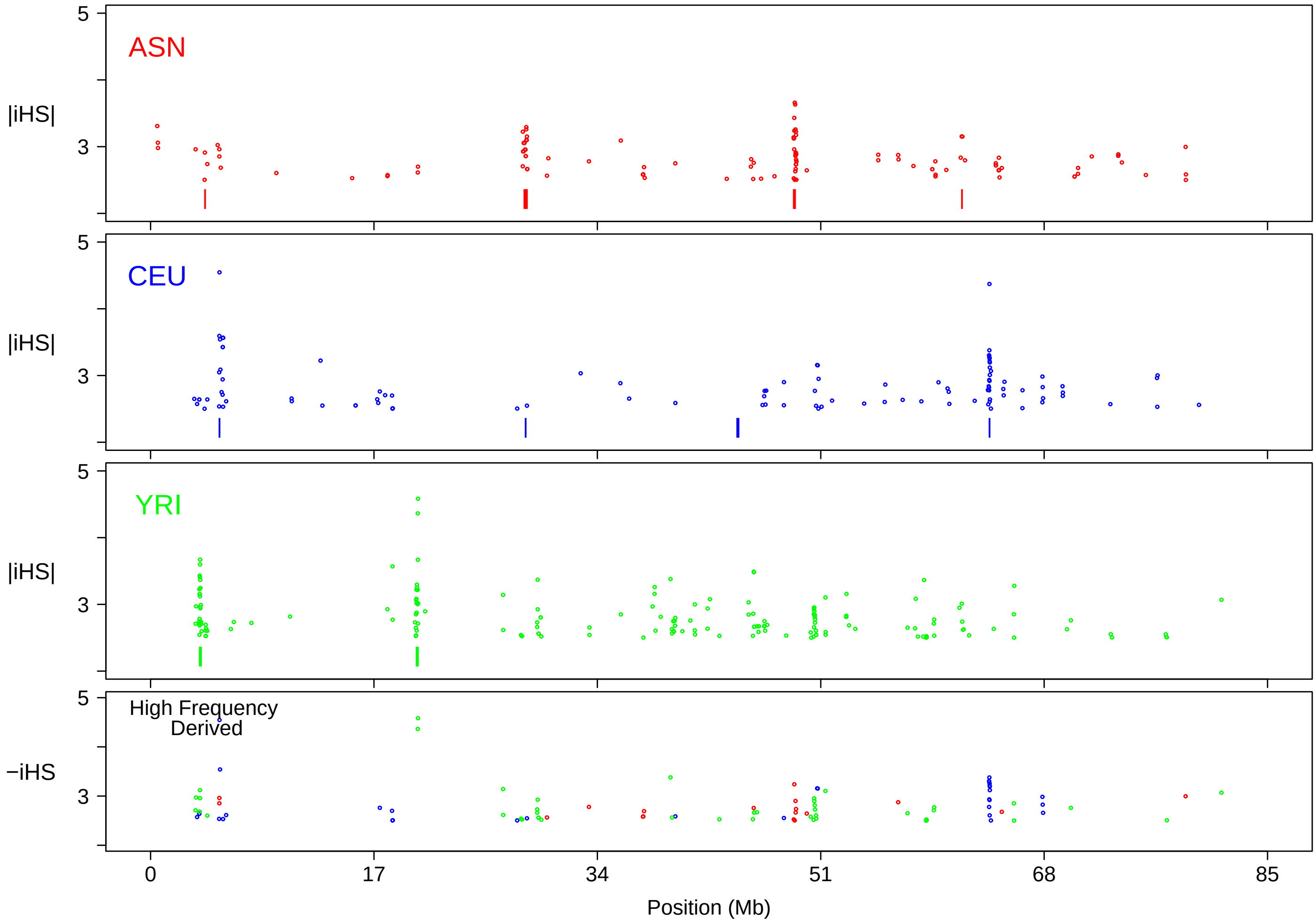


Figure S19: **Plots of Chromosome 17 SNPs with extreme iHS values illustrate discrete clusters of signals.** SNPs with $|\text{iHS}| > 2.5$ (top 1%) are plotted. The bottom plot combines signals for all three populations, plotting only SNPs with derived frequency $> 0.5$ and iHS $< -2.5$. Such SNPs correspond to high frequency derived SNPs in the range for which our test is most powerful. The short vertical bars below each plot indicate 100Kb windows whose signals are in the top 1% of windows genome-wide.

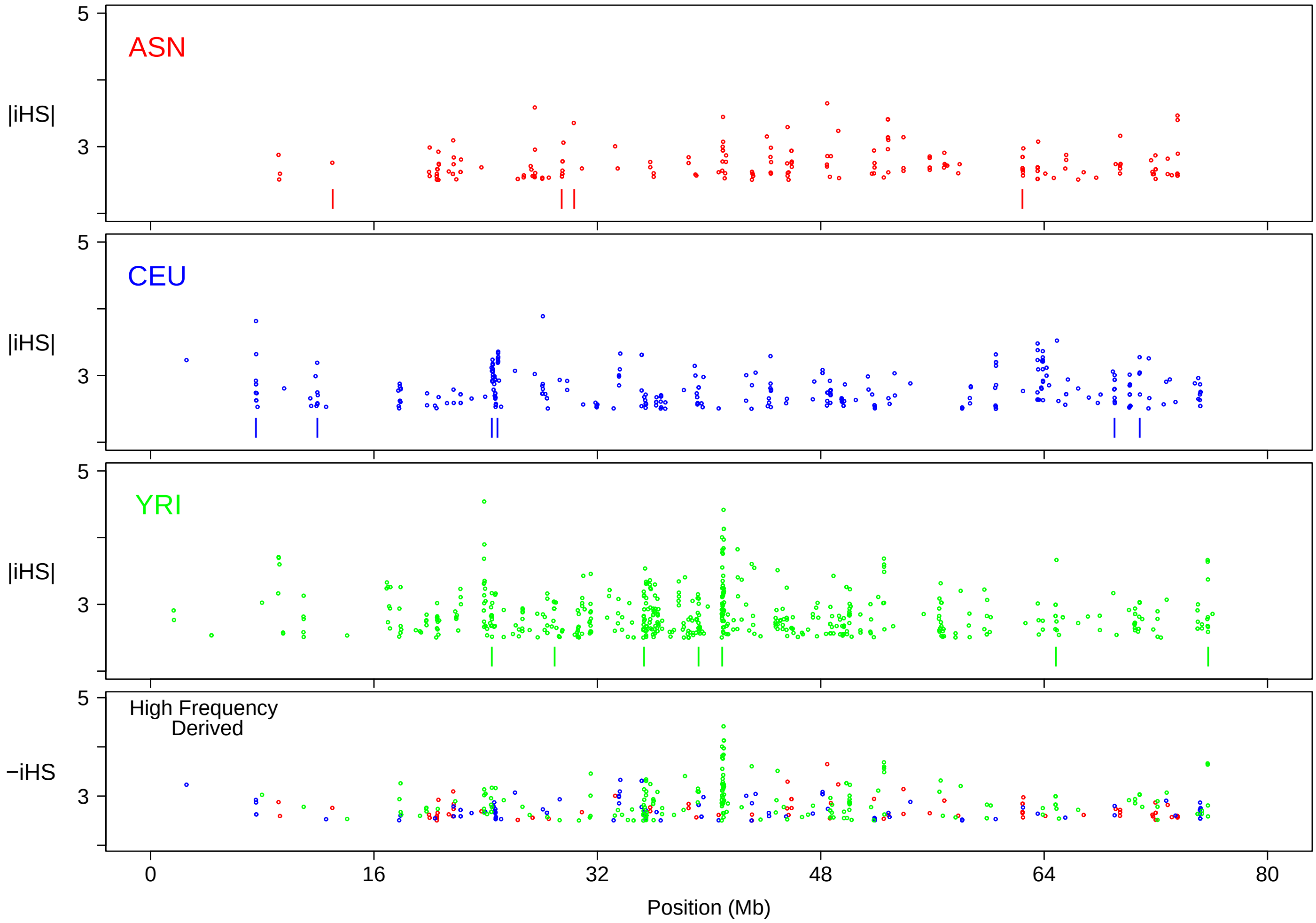


Figure S20: **Plots of Chromosome 18 SNPs with extreme iHS values illustrate discrete clusters of signals.** SNPs with |iHS| > 2.5 (top 1%) are plotted. The bottom plot combines signals for all three populations, plotting only SNPs with derived frequency > 0.5 and iHS < −2.5. Such SNPs correspond to high frequency derived SNPs in the range for which our test is most powerful. The short vertical bars below each plot indicate 100Kb windows whose signals are in the top 1% of windows genome-wide.

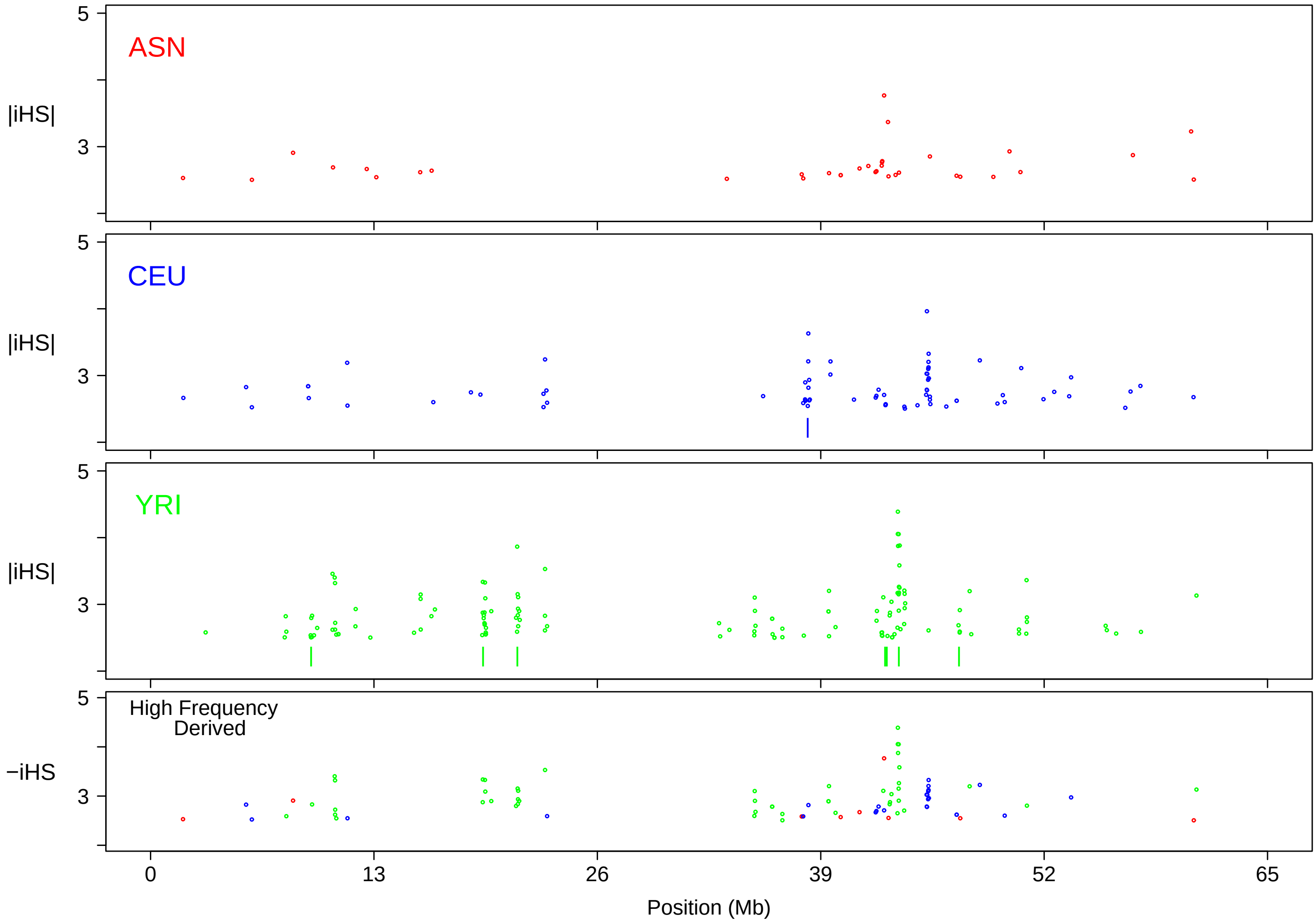


Figure S21: **Plots of Chromosome 19 SNPs with extreme iHS values illustrate discrete clusters of signals.** SNPs with |iHS| $> 2.5$ (top 1%) are plotted. The bottom plot combines signals for all three populations, plotting only SNPs with derived frequency $> 0.5$ and iHS $< -2.5$. Such SNPs correspond to high frequency derived SNPs in the range for which our test is most powerful. The short vertical bars below each plot indicate 100Kb windows whose signals are in the top 1% of windows genome-wide.

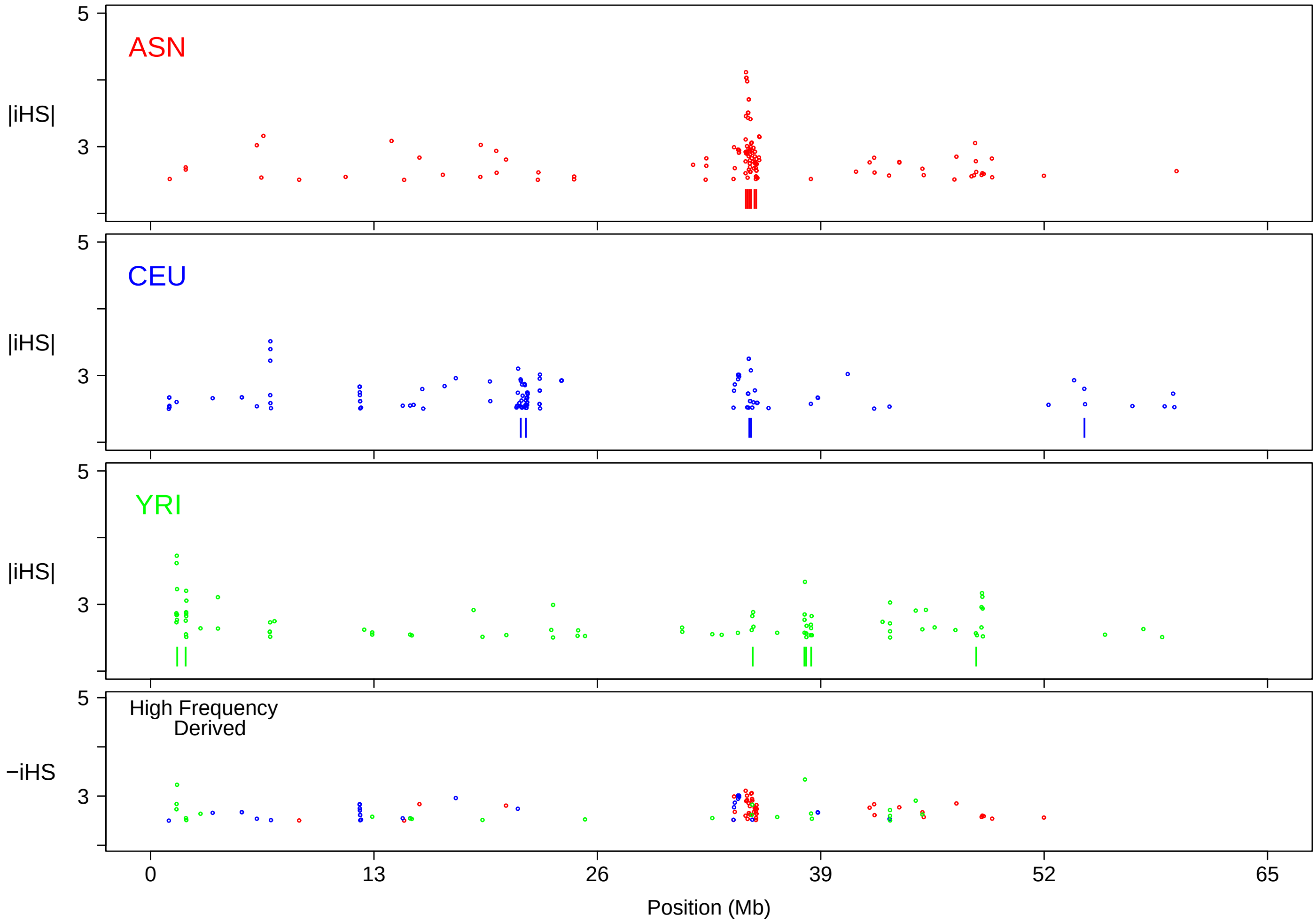


Figure S22: **Plots of Chromosome 20 SNPs with extreme iHS values illustrate discrete clusters of signals.** SNPs with |iHS| > 2.5 (top 1%) are plotted. The bottom plot combines signals for all three populations, plotting only SNPs with derived frequency > 0.5 and iHS < −2.5. Such SNPs correspond to high frequency derived SNPs in the range for which our test is most powerful. The short vertical bars below each plot indicate 100Kb windows whose signals are in the top 1% of windows genome-wide.

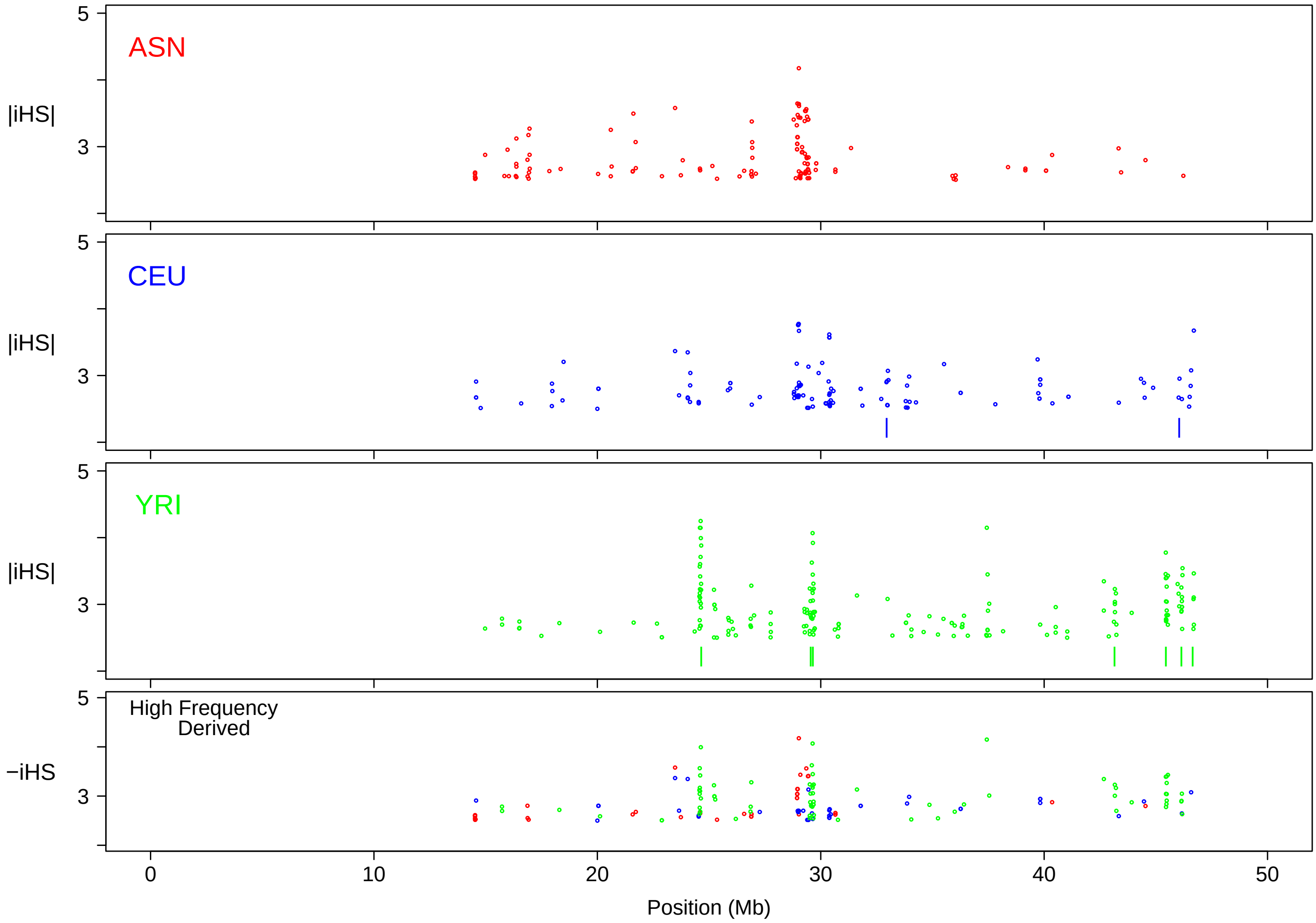


Figure S23: **Plots of Chromosome 21 SNPs with extreme iHS values illustrate discrete clusters of signals.** SNPs with |iHS| $> 2.5$ (top 1%) are plotted. The bottom plot combines signals for all three populations, plotting only SNPs with derived frequency $> 0.5$ and iHS $< -2.5$. Such SNPs correspond to high frequency derived SNPs in the range for which our test is most powerful. The short vertical bars below each plot indicate 100Kb windows whose signals are in the top 1% of windows genome-wide.

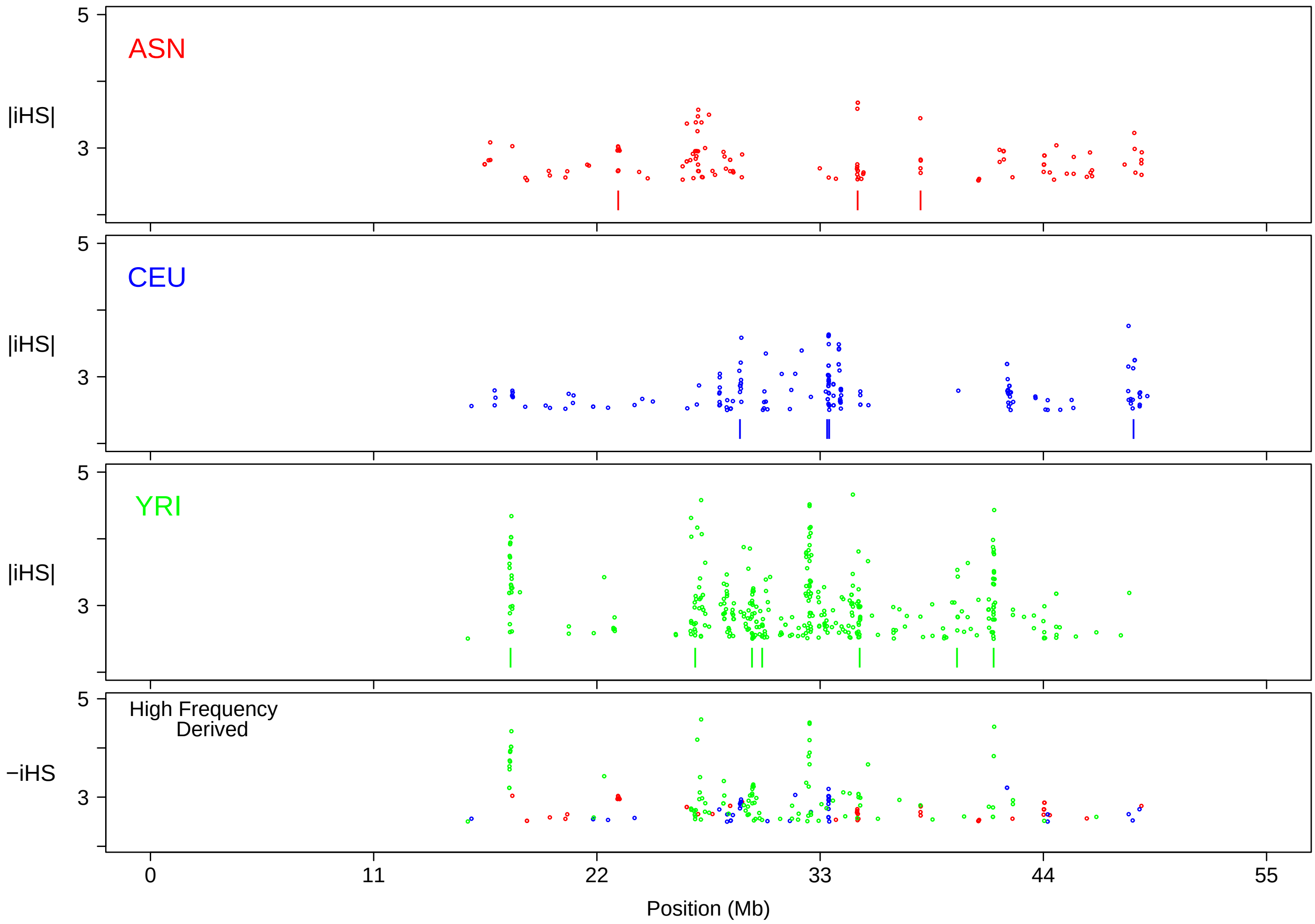


Figure S24: **Plots of Chromosome 22 SNPs with extreme iHS values illustrate discrete clusters of signals.** SNPs with |iHS| > 2.5 (top 1%) are plotted. The bottom plot combines signals for all three populations, plotting only SNPs with derived frequency > 0.5 and iHS < −2.5. Such SNPs correspond to high frequency derived SNPs in the range for which our test is most powerful. The short vertical bars below each plot indicate 100Kb windows whose signals are in the top 1% of windows genome-wide.

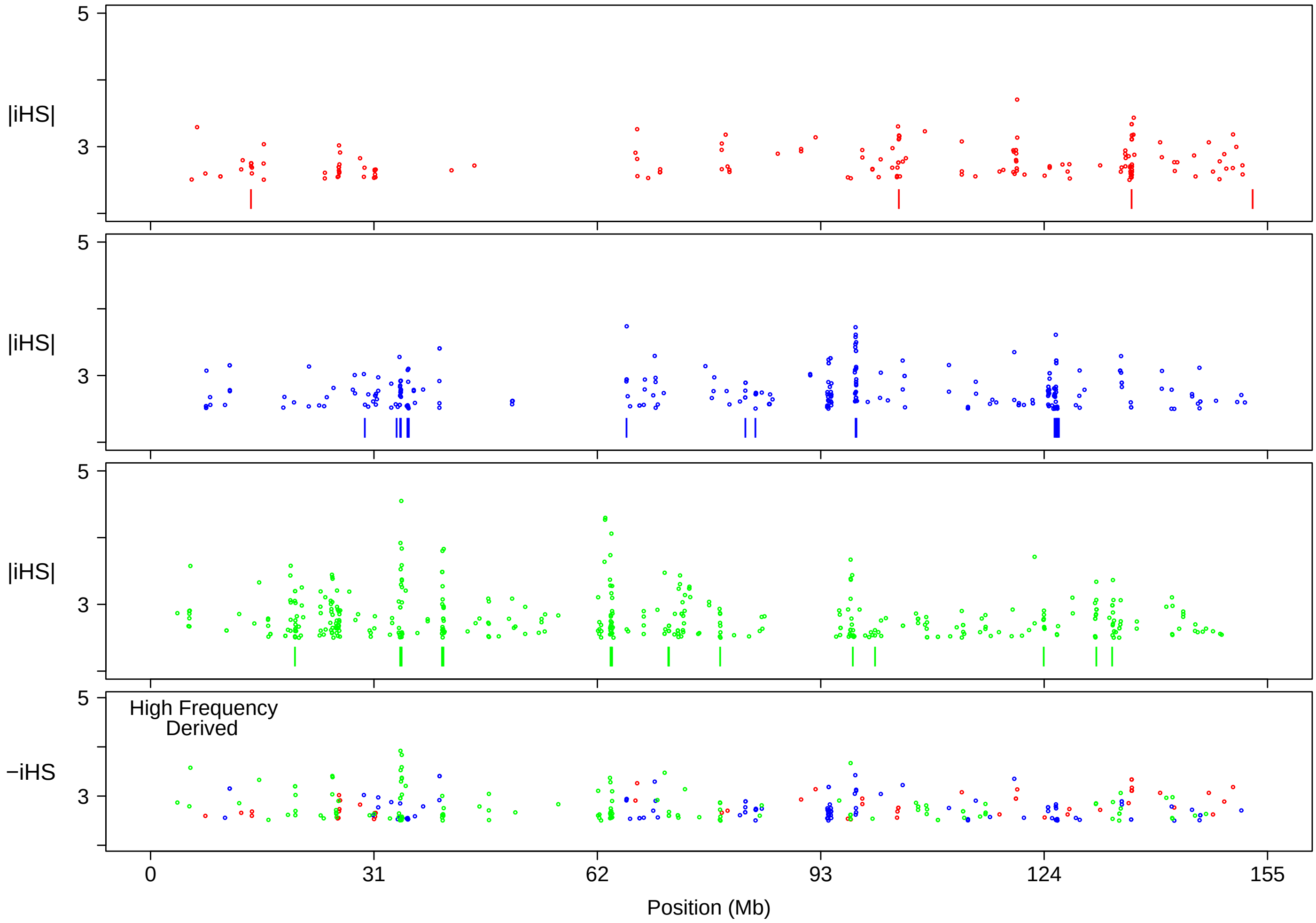


Figure S25: **Plots of the X Chromosome SNPs with extreme iHS values illustrate discrete clusters of signals.** SNPs with $|\text{iHS}| > 2.5$ (top 1%) are plotted. The bottom plot combines signals for all three populations, plotting only SNPs with derived frequency $> 0.5$ and iHS $< -2.5$. Such SNPs correspond to high frequency derived SNPs in the range for which our test is most powerful. The short vertical bars below each plot indicate 100Kb windows whose signals are in the top 1% of windows genome-wide.

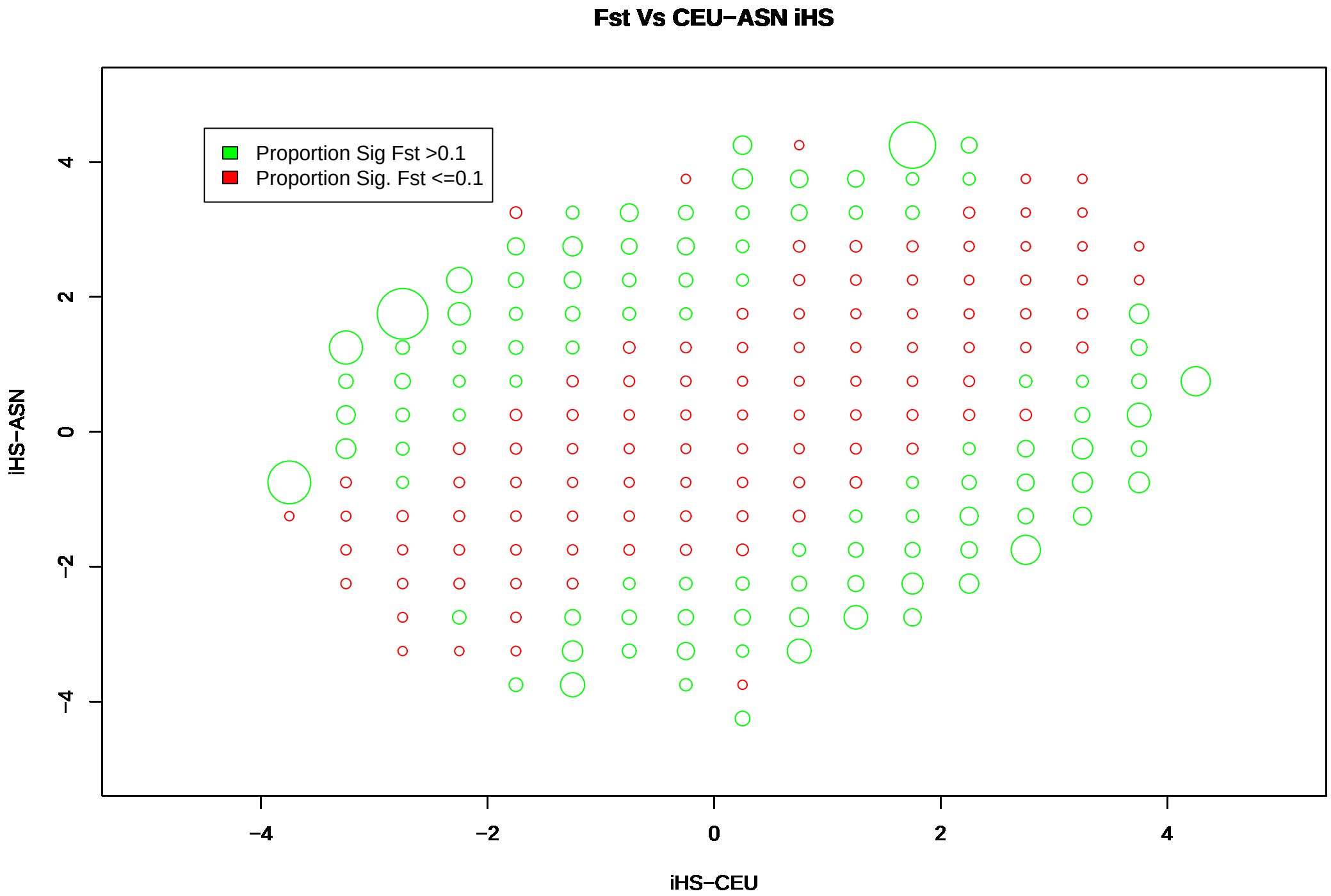


Figure S26: **Plot comparing Fst with iHS (CEU and ASN).** iHS in each population were grouped into bins of 0.5. The size of the circles in the plot represent the proportion of the SNPs with Fst greater than the genome wide empirical 5% threshold. Circles in red represent bins where the proportion of SNPs with Fst greater than the 5% threshold exceeds 10% and those in green represent bins where the proportion is less than 10%.

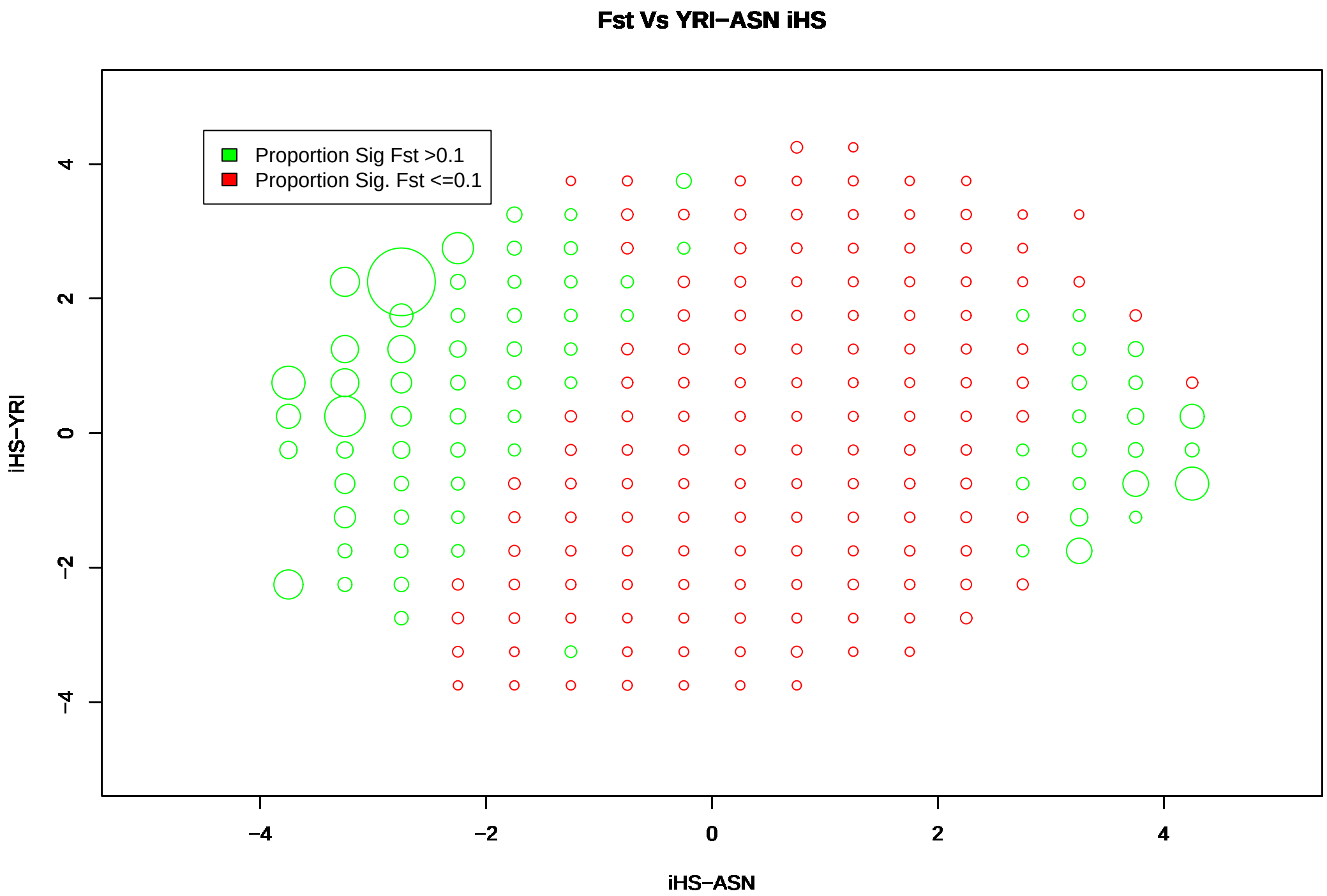


Figure S27: **Plot comparing Fst with iHS (ASN and YRI).** iHS in each population were grouped into bins of 0.5. The size of the circles in the plot represent the proportion of the SNPs with Fst greater than the genome wide empirical 5% threshold. Circles in red represent bins where the proportion of SNPs with Fst greater than the 5% threshold exceeds 10% and those in green represent bins where the proportion is less than 10%.

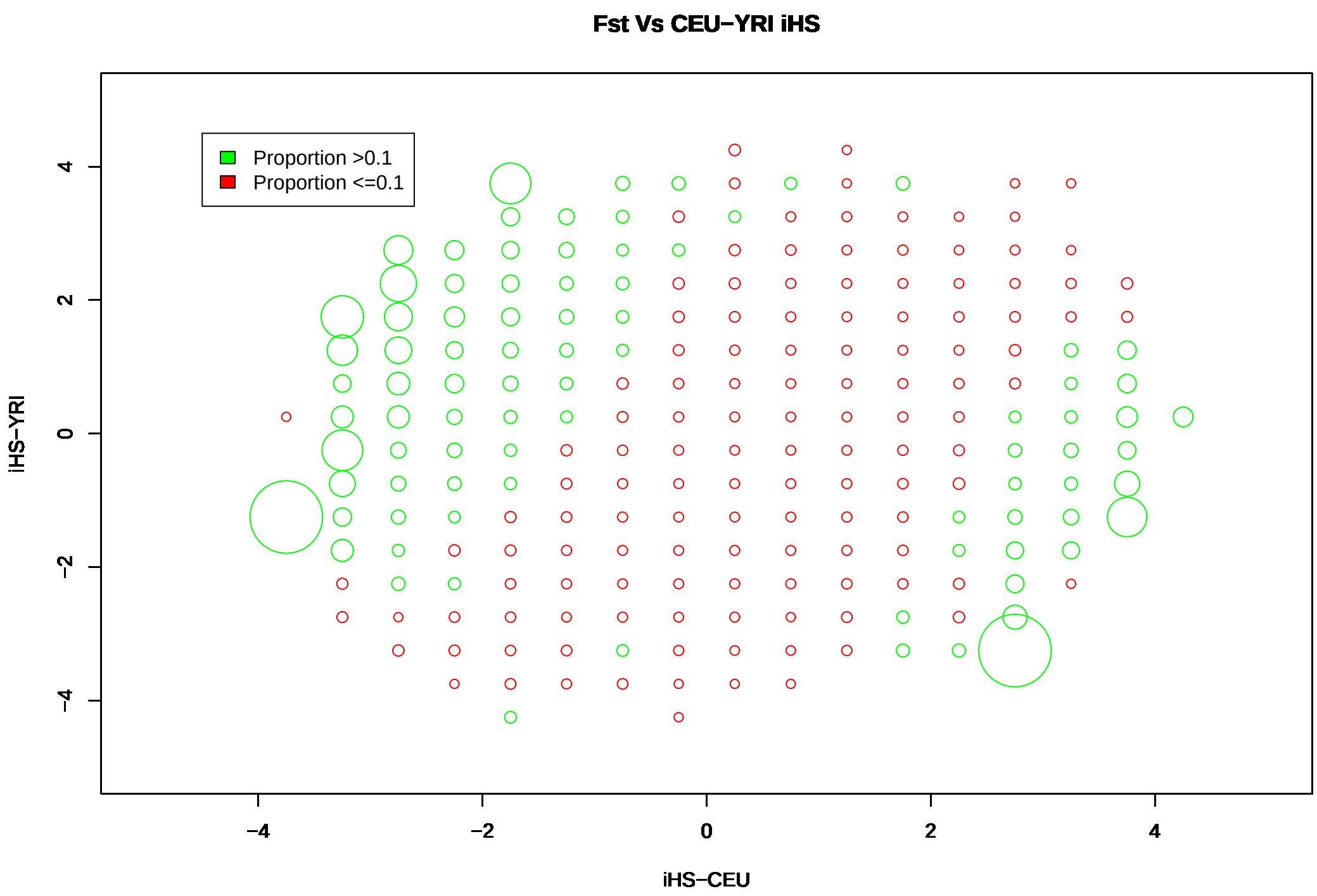


Figure S28: **Plot comparing Fst with iHS (CEU and YRI).** iHS in each population were grouped into bins of 0.5. The size of the circles in the plot represent the proportion of the SNPs with Fst greater than the genome wide empirical 5% threshold. Circles in red represent bins where the proportion of SNPs with Fst greater than the 5% threshold exceeds 10% and those in green represent bins where the proportion is less than 10%.

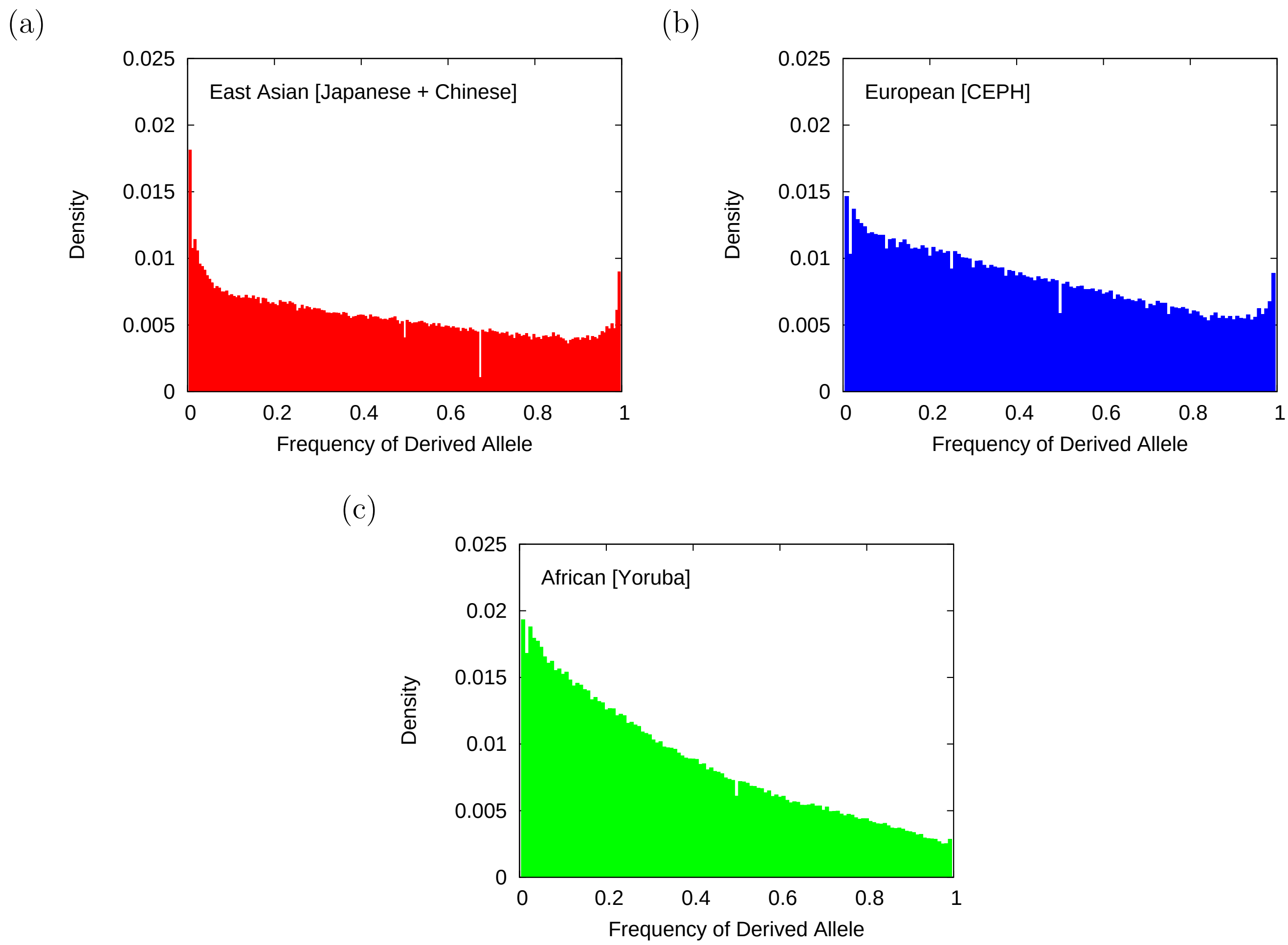


Figure S29: **Polarized frequency spectra for each HapMap population.**

| | CEU | CEU | YRI | YRI | ASN | ASN |
|---|---|---|---|---|---|---|
| | \|iHS\| $>$ 2.0 | \|iHS\| $\leq$ 2.0 | \|iHS\| $>$ 2.0 | \|iHS\| $\leq$ 2.0 | \|iHS\| $>$ 2.0 | \|iHS\| $\leq$ 2.0 |
| Genic | 13619 | 267085 | 14783 | 272331 | 12513 | 249407 |
| Nongenic | 15565 | 354514 | 16195 | 367950 | 14348 | 322565 |
| OR (95% CI) | | 1.16(1.14–1.19) | | 1.23(1.20–1.26) | | 1.13(1.1–1.16) |
| | \|iHS\| $>$ 2.0 | \|iHS\| $\leq$ 2.0 | \|iHS\| $>$ 2.0 | \|iHS\| $\leq$ 2.0 | \|iHS\| $>$ 2.0 | \|iHS\| $\leq$ 2.0 |
| Overlapping Genes | 261 | 4474 | 297 | 4473 | 263 | 4240 |
| Single Genes | 13358 | 262611 | 14486 | 267858 | 12250 | 245167 |
| OR (95% CI) | | 1.15(1.01–1.3) | | 1.22(1.09–1.38) | | 1.24(1.09–1.41) |

Table S1: **iHS signal in genic versus non-genic regions.** Table comparing the number of SNPs in genic and nongenic regions with high |iHS| score. The lower table compares the number of SNPs with high |iHS| that are represented in multiple overlapping open reading frames (ORFs) to those present in single ORF.

| Population | rsnumber | iHS | cM Span | Genes |
|---|---|---|---|---|
| East Asian | rs4971100 | -2.842 | 1.39 | GBA, MTX1, ADAM15, LENEP * |
| | rs7441655 | -2.344 | 1.29 | NEK1, SH3MD2,CLCN3 * |
| | rs1442490 | -2.665 | 1.16 | ADH cluster, MTP * |
| CEPH | rs6035877 | -2.194 | 1.25 | XRN2, PAX1, NKX2-2 * |
| | rs1426721 | -2.237 | 1.15 | SLC24A5, SLC12A1, MYEF2, FBN1 * |
| | rs6497253 | -3.118 | 1.13 | OCA2, HERC2 |
| Yoruba | rs1160025 | -2.577 | 0.97 | FBXL7 |
| | rs13332060 | -2.547 | 0.94 | ATPV0C, CCNF, NTN2L, PDPK1 * |
| | rs9536046 | -4.15 | 0.74 | NEK3, ATP7B, CKAP2, LECT1 * |

Table S2: **Genetic spans of the longest haplotypes in each population.** Recorded are the SNPs with derived allele frequency $> 50\%$ that have the longest spans between the first point to the left, and first point to the right of the core SNP at which EHH drops below 0.25. The SNPs considered for this analysis were those SNPs with the most negative iHS in each of the top 1% of all surveyed regions. * indicates that the genes listed in the table are among many genes in the region spanned by the haplotypes. The span around the OCA2 gene was manually truncated due to a large gap in the SNP data on one side.

| | CEU | CEU | YRI | YRI | ASN | ASN |
|---|---|---|---|---|---|---|
| | iHS S | iHS NS | iHS S | iHS NS | iHS S | iHS NS |
| $P_2 < 0.05$ | 61 | 492 | 41 | 426 | 61 | 475 |
| $P_2 \geq 0.05$ | 420 | 4602 | 438 | 4660 | 418 | 4545 |
| $\chi^2$(pvalue) | | 4.5(<0.035) | | 0.02(<0.9) | | 5.32(<0.025) |

Table S3: **Comparison to Clark et al. (2003) study.** Independence test between genes tested via the iHS and the genes testing significant (Model 2) along the human lineage by Clark et al. (2003).

| GO Nesting | GO Category | ASN | CEU | YRI |
|---|---|---|---|---|
| 21-1 | Chemosensory Perception | NS | <0.0006[A] | <0.0004[B] |
| 21-1-1 | Olfaction | NS | <0.0006[A] | <0.0008[B] |
| 22-2 | Gametogenesis | <0.008[C] | NS | NS |
| 22-2-2 | Spermatogenesis and Motility | <0.02 | <0.03 | NS |
| 22-3 | Fertilization | <0.004 | <0.003 | NS |
| 1-11 | Other Carbohydrate Metabolism | <**0.0002**[D] | NS | NS |
| 6 | Electron Transport | NS | <**0.0002**[E] | NS |
| 4-13 | Chromatin Packaging/Remodeling | <**0.0001**[F] | <0.01[G] | NS |
| 16-1-1 | MHC-I Mediated Immunity | NS | <**0.0001**[H] | <0.02 |
| 3-2 | Steroid Metabolism | NS | NS | <**0.0001**[I] |
| 3-5 | Lipid and Fatty Acid Binding | <0.001 | NS | NS |
| 4-4-2 | mRNA Transcription Initiation | NS | <0.002[J] | NS |
| 5-3 | Protein Modification | <0.002[K] | NS | NS |
| 7-5 | Vitamin/Cofactor Transport | <0.002[L] | NS | NS |
| 9 | Phosphate Metabolism | <0.002[M] | <0.03[N] | NS |
| 13-4 | Peroxisome Transport | NS | NS | <0.002 |

Table S4: **Gene clusters that contribute to significant GO categories.** The significance in some categories can be partially attributed to clusters of significant genes with similar ontological function. The clusters are in the following locations, with total numbers of genes showing signals in square brackets, and the numbers in each cluster in parentheses: **A**: [29], 1q44 (11), 6p22.1 (10); **B**: [34], 11p15.4 (8), 11q11 (6), 11p11.2 (5); **C**: [28], 6p21-22 (5); **D**: [9], 4q21-23 (7); **E**: [27] 1p33-34 (6), 7q21-22 (5); **F**: [37] 6p21.3 (29); **G**: [13], 6p21.3 (10); **H**: [7] 6p21.3 (7); **I**: [23], 22q12-13 (8); **J**: [13], 2q33.1 (3); **K**: [109], 3p21 (7), 11q13 (5), 20q11 (5), 22q11 (4); **L**: [8], 3q23 (2), 11q13-14 (2); **M**: [8] 6p21 (3); **N**: [11] 6p21 (3).

# Demographic Simulation Supplement

Demographic simulations were performed using the coalescent program, `ms` (Hudson, 2001), as described in the Methods. Simulations assumed an ancestral population size of 10,659 and a total epoch of 40K. "**S**" denotes the number of segregating sites simulated, "$\rho$" the recombination rate, and "**L**" the survey length. The simulations were performed assuming a hotspot model, which is why the values for **S**, $\rho$, and **L** vary from simulation to simulation. The exact details regarding the recombination rate and demographic models are also described in the Methods.

## Demgraphic models for East Asia [ASN]

Bottleneck for $t_{dur}$ generations, instantaneous recovery to $N_A$ afterwards.
$t_{dur} = 300$, b = 0.05: ms 178 1 **S** -r $\rho$ **L** -eN 0.037527 1.0 -eN 0.03049 0.05 -eN 0.0 1.0
$t_{dur} = 800$, b = 0.05: ms 178 1 **S** -r $\rho$ **L** -eN 0.037527 1.0 -eN 0.01876 0.05 -eN 0.0 1.0
$t_{dur} = 600$, b = 0.1: ms 178 1 -s **S** -r $\rho$ **L** -eN 0.037527 1.0 -eN 0.02345 0.1 -eN 0.0 1.0
$t_{dur} = 800$, b = 0.2: ms 178 1 -s **S** -r $\rho$ **L** -eN 0.037527 1.0 -eN 0.01876 0.2 -eN 0.0 1.0
$t_{dur} = 1200$, b = 0.2: ms 178 1 **S** -r $\rho$ **L** -eN 0.037527 1.0 -eN 0.009382 0.2 -eN 0.0 1.0
$t_{dur} = 1500$, b = 0.275: ms 178 1 -s **S** -r $\rho$ **L** -eN 0.037527 1.0 -eN 0.002345 0.275 -eN 0.0 1.0

Bottleneck for $t_{dur}$ generations, instantaneous recovery to $5 \cdot N_A$ afterwards.
$t_{dur} = 300$, b = 0.05: ms 178 1 -s **S** -r $\rho$ **L** -eN 0.037527 1.0 -eN 0.03049 0.05 -eN 0.0 5.0
$t_{dur} = 800$, b = 0.05: ms 178 1 -s **S** -r $\rho$ **L** -eN 0.037527 1.0 -eN 0.01876 0.05 -eN 0.0 5.0
$t_{dur} = 600$, b = 0.1: ms 178 1 -s **S** -r $\rho$ **L** -eN 0.037527 1.0 -eN 0.02345 0.1 -eN 0.0 5.0
$t_{dur} = 800$, b = 0.2: ms 178 1 -s **S** -r $\rho$ **L** -eN 0.037527 1.0 -eN 0.01876 0.2 -eN 0.0 5.0
$t_{dur} = 1200$, b = 0.2: ms 178 1 -s **S** -r $\rho$ **L** -eN 0.037527 1.0 -eN 0.009382 0.2 -eN 0.0 5.0
$t_{dur} = 1500$, b = 0.275: ms 178 1 -s **S** -r $\rho$ **L** -eN 0.037527 1.0 -eN 0.002345 0.275 -eN 0.0 5.0

Bottleneck for $t_{dur}$ generations, instantaneous recovery to $0.5 \cdot N_A$ afterwards.
$t_{dur} = 300$, b = 0.05: ms 178 1 -s **S** -r $\rho$ **L** -eN 0.037527 1.0 -eN 0.03049 0.05 -eN 0.0 0.5
$t_{dur} = 800$, b = 0.05: ms 178 1 -s **S** -r $\rho$ **L** -eN 0.037527 1.0 -eN 0.01876 0.05 -eN 0.0 0.5
$t_{dur} = 600$, b = 0.1: ms 178 1 -s **S** -r $\rho$ **L** -eN 0.037527 1.0 -eN 0.02345 0.1 -eN 0.0 0.5
$t_{dur} = 1200$, b = 0.2: ms 178 1 -s **S** -r $\rho$ **L** -eN 0.037527 1.0 -eN 0.009382 0.2 -eN 0.0 0.5
$t_{dur} = 1500$, b = 0.275: ms 178 1 -s **S** -r $\rho$ **L** -eN 0.037527 1.0 -eN 0.002345 0.275 -eN 0.0 0.5

## Demgraphic models for CEPH [CEU]

Bottleneck for $t_{dur}$ generations, instantaneous recovery to $N_A$ afterwards.
$t_{dur}$ = 300, b = 0.05: ms 120 1 -s **S** -r $\rho$ **L** -eN 0.037527 1.0 -eN 0.03049 0.05 -eN 0.0 1.0
$t_{dur}$ = 800, b = 0.05: ms 120 1 -s **S** -r $\rho$ **L** -eN 0.037527 1.0 -eN 0.01876 0.05 -eN 0.0 1.0
$t_{dur}$ = 600, b = 0.1: ms 120 1 -s **S** -r $\rho$ **L** -eN 0.037527 1.0 -eN 0.02345 0.1 -eN 0.0 1.0
$t_{dur}$ = 800, b = 0.2: ms 120 1 -s **S** -r $\rho$ **L** -eN 0.037527 1.0 -eN 0.01876 0.2 -eN 0.0 1.0
$t_{dur}$ = 1200, b = 0.2: ms 120 1 -s **S** -r $\rho$ **L** -eN 0.037527 1.0 -eN 0.009382 0.2 -eN 0.0 1.0
$t_{dur}$ = 1500, b = 0.275: ms 120 1 -s **S** -r $\rho$ **L** -eN 0.037527 1.0 -eN 0.002345 0.275 -eN 0.0 1.0

Bottleneck for $t_{dur}$ generations, instantaneous recovery to $5 \cdot N_A$ afterwards.
$t_{dur}$ = 800, b = 0.05: ms 120 1 -s **S** -r $\rho$ **L** -eN 0.037527 1.0 -eN 0.01876 0.05 -eN 0.0 5.0
$t_{dur}$ = 800, b = 0.2: ms 120 1 -s **S** -r $\rho$ **L** -eN 0.037527 1.0 -eN 0.01876 0.2 -eN 0.0 5.0
$t_{dur}$ = 1200, b = 0.2: ms 120 1 -s **S** -r $\rho$ **L** -eN 0.037527 1.0 -eN 0.009382 0.2 -eN 0.0 5.0
$t_{dur}$ = 1500, b = 0.275: ms 120 1 -s **S** -r $\rho$ **L** -eN 0.037527 1.0 -eN 0.002345 0.275 -eN 0.0 5.0

Bottleneck for $t_{dur}$ generations, instantaneous recovery to $0.5 \cdot N_A$ afterwards.
$t_{dur}$ = 300, b = 0.05: ms 120 1 -s **S** -r $\rho$ **L** -eN 0.037527 1.0 -eN 0.03049 0.05 -eN 0.0 0.5
$t_{dur}$ = 800, b = 0.05: ms 120 1 -s **S** -r $\rho$ **L** -eN 0.037527 1.0 -eN 0.01876 0.05 -eN 0.0 0.5
$t_{dur}$ = 600, b = 0.1: ms 120 1 -s **S** -r $\rho$ **L** -eN 0.037527 1.0 -eN 0.02345 0.1 -eN 0.0 0.5
$t_{dur}$ = 800, b = 0.2: ms 120 1 -s **S** -r $\rho$ **L** -eN 0.037527 1.0 -eN 0.01876 0.2 -eN 0.0 0.5
$t_{dur}$ = 1200, b = 0.2: ms 120 1 -s **S** -r $\rho$ **L** -eN 0.037527 1.0 -eN 0.009382 0.2 -eN 0.0 0.5
$t_{dur}$ = 1500, b = 0.275: ms 120 1 -s **S** -r $\rho$ **L** -eN 0.037527 1.0 -eN 0.002345 0.275 -eN 0.0 0.5

## Demgraphic models for Yoruba [YRI]

Exponential growth from initial size $N_A$ to $N_0$, occurring $t_{onset}$ generations in the past.
$N_A = 11156$, $N_0 = 11156$, $t_{onset}$: 1000: ms 120 1 -s **S** -r $\rho$ **L** -eG 0.02241 0
$N_A = 10659$, $N_0 = 22600$, $t_{onset}$: 1000: ms 120 1 -s **S** -r $\rho$ **L** -eG 0.02345 31.977
$N_A = 7834$, $N_0 = 57900$, $t_{onset}$: 4000: ms 120 1 -s **S** -r $\rho$ **L** -eG 0.12765 15.668
$N_A = 10424$, $N_0 = 938000$, $t_{onset}$: 500: ms 120 1 -s **S** -r $\rho$ **L** -eG 0.01199 375.264
$N_A = 9944$, $N_0 = 44600$, $t_{onset}$: 1500: ms 120 1 -s **S** -r $\rho$ **L** -eG 0.03771 39.776
$N_A = 11020$, $N_0 = 23300$, $t_{onset}$: 250: ms 120 1 -s **S** -r $\rho$ **L** -eG 0.005672 132.24
$N_A = 9912$, $N_0 = 199000$, $t_{onset}$: 1000: ms 120 1 -s **S** -r $\rho$ **L** -eG 0.02522 118.944
$N_A = 10018$, $N_0 = 1910000$, $t_{onset}$: 750: ms 120 1 -s **S** -r $\rho$ **L** -eG 0.01872 280.504
