# Supplementary material for: A Map of Recent Positive Selection in the Human Genome
Source: PLoS Biol. 2006 Mar 7;4(3):e72. doi: 10.1371/journal.pbio.0040072 (PMC1382018; doi:10.1371/journal.pbio.0040072)
Supplement: Supplementary file 31 [file pbio.0040072.st003.pdf]

	CEU	CEU	YRI	YRI	ASN	ASN
	iHS S	iHS NS	iHS S	iHS NS	iHS S	iHS NS
$P_2 < 0.05$	61	492	41	426	61	475
$P_2 \geq 0.05$	420	4602	438	4660	418	4545
χ^2(pvalue)		4.5(<0.035)		0.02(<0.9)		5.32(<0.025)

Table S3: **Comparison to Clark et al. (2003) study.** Independence test between genes tested via the iHS and the genes testing significant (Model 2) along the human lineage by Clark et al. (2003).
