# Supplementary material for: A Map of Recent Positive Selection in the Human Genome
Source: PLoS Biol. 2006 Mar 7;4(3):e72. doi: 10.1371/journal.pbio.0040072 (PMC1382018; doi:10.1371/journal.pbio.0040072)
Supplement: Supplementary file 32 [file pbio.0040072.sd004.doc]

[];a       
Demographic Simulation Supplement
Demographic simulations were performed using the coalescent program, ms (Hudson, 2001), as described in the Methods. Simulations assumed an ancestral population size of 10,659 and a total epoch of 40K. “" denotes the number of segregating sites simulated, “" the recombination rate, and “L" the survey length. The simulations were performed assuming a hotspot model, which is why the values for S, , and L vary from simulation to simulation. The exact details regarding the recombination rate and demographic models are also described in the Methods. 
Demgraphic models for East Asia [ASN]
1.	Bottleneck for  generations, instantaneous recovery to  afterwards. 
2.	 = 300, b = 0.05: ms 178 1  -r   -eN 0.037527 1.0 -eN 0.03049 0.05 -eN 0.0 1.0 
3.	 = 800, b = 0.05: ms 178 1  -r   -eN 0.037527 1.0 -eN 0.01876 0.05 -eN 0.0 1.0 
4.	 = 600, b = 0.1: ms 178 1 -s  -r   -eN 0.037527 1.0 -eN 0.02345 0.1 -eN 0.0 1.0 
5.	 = 800, b = 0.2: ms 178 1 -s  -r   -eN 0.037527 1.0 -eN 0.01876 0.2 -eN 0.0 1.0 
6.	 = 1200, b = 0.2: ms 178 1  -r   -eN 0.037527 1.0 -eN 0.009382 0.2 -eN 0.0 1.0 
7.	 = 1500, b = 0.275: ms 178 1 -s  -r   -eN 0.037527 1.0 -eN 0.002345 0.275 -eN 0.0 1.0  
8.	Bottleneck for  generations, instantaneous recovery to  afterwards. 
9.	 = 300, b = 0.05: ms 178 1 -s  -r   -eN 0.037527 1.0 -eN 0.03049 0.05 -eN 0.0 5.0 
10.	 = 800, b = 0.05: ms 178 1 -s  -r   -eN 0.037527 1.0 -eN 0.01876 0.05 -eN 0.0 5.0 
11.	 = 600, b = 0.1: ms 178 1 -s  -r   -eN 0.037527 1.0 -eN 0.02345 0.1 -eN 0.0 5.0 
12.	 = 800, b = 0.2: ms 178 1 -s  -r   -eN 0.037527 1.0 -eN 0.01876 0.2 -eN 0.0 5.0 
13.	 = 1200, b = 0.2: ms 178 1 -s  -r   -eN 0.037527 1.0 -eN 0.009382 0.2 -eN 0.0 5.0 
14.	 = 1500, b = 0.275: ms 178 1 -s  -r   -eN 0.037527 1.0 -eN 0.002345 0.275 -eN 0.0 5.0  
15.	Bottleneck for  generations, instantaneous recovery to  afterwards. 
16.	 = 300, b = 0.05: ms 178 1 -s  -r   -eN 0.037527 1.0 -eN 0.03049 0.05 -eN 0.0 0.5 
17.	 = 800, b = 0.05: ms 178 1 -s  -r   -eN 0.037527 1.0 -eN 0.01876 0.05 -eN 0.0 0.5 
18.	 = 600, b = 0.1: ms 178 1 -s  -r   -eN 0.037527 1.0 -eN 0.02345 0.1 -eN 0.0 0.5 
19.	 = 1200, b = 0.2: ms 178 1 -s  -r   -eN 0.037527 1.0 -eN 0.009382 0.2 -eN 0.0 0.5 
20.	 = 1500, b = 0.275: ms 178 1 -s  -r   -eN 0.037527 1.0 -eN 0.002345 0.275 -eN 0.0 0.5  
Demgraphic models for CEPH [CEU]
1.	Bottleneck for  generations, instantaneous recovery to  afterwards. 
2.	 = 300, b = 0.05: ms 120 1 -s  -r   -eN 0.037527 1.0 -eN 0.03049 0.05 -eN 0.0 1.0 
3.	 = 800, b = 0.05: ms 120 1 -s  -r   -eN 0.037527 1.0 -eN 0.01876 0.05 -eN 0.0 1.0 
4.	 = 600, b = 0.1: ms 120 1 -s  -r   -eN 0.037527 1.0 -eN 0.02345 0.1 -eN 0.0 1.0 
5.	 = 800, b = 0.2: ms 120 1 -s  -r   -eN 0.037527 1.0 -eN 0.01876 0.2 -eN 0.0 1.0 
6.	 = 1200, b = 0.2: ms 120 1 -s  -r   -eN 0.037527 1.0 -eN 0.009382 0.2 -eN 0.0 1.0 
7.	 = 1500, b = 0.275: ms 120 1 -s  -r   -eN 0.037527 1.0 -eN 0.002345 0.275 -eN 0.0 1.0  
8.	Bottleneck for  generations, instantaneous recovery to  afterwards. 
9.	 = 800, b = 0.05: ms 120 1 -s  -r   -eN 0.037527 1.0 -eN 0.01876 0.05 -eN 0.0 5.0 
10.	 = 800, b = 0.2: ms 120 1 -s  -r   -eN 0.037527 1.0 -eN 0.01876 0.2 -eN 0.0 5.0 
11.	 = 1200, b = 0.2: ms 120 1 -s  -r   -eN 0.037527 1.0 -eN 0.009382 0.2 -eN 0.0 5.0 
12.	 = 1500, b = 0.275: ms 120 1 -s  -r   -eN 0.037527 1.0 -eN 0.002345 0.275 -eN 0.0 5.0  
13.	Bottleneck for  generations, instantaneous recovery to  afterwards. 
14.	 = 300, b = 0.05: ms 120 1 -s  -r   -eN 0.037527 1.0 -eN 0.03049 0.05 -eN 0.0 0.5 
15.	 = 800, b = 0.05: ms 120 1 -s  -r   -eN 0.037527 1.0 -eN 0.01876 0.05 -eN 0.0 0.5 
16.	 = 600, b = 0.1: ms 120 1 -s  -r   -eN 0.037527 1.0 -eN 0.02345 0.1 -eN 0.0 0.5 
17.	 = 800, b = 0.2: ms 120 1 -s  -r   -eN 0.037527 1.0 -eN 0.01876 0.2 -eN 0.0 0.5 
18.	 = 1200, b = 0.2: ms 120 1 -s  -r   -eN 0.037527 1.0 -eN 0.009382 0.2 -eN 0.0 0.5 
19.	 = 1500, b = 0.275: ms 120 1 -s  -r   -eN 0.037527 1.0 -eN 0.002345 0.275 -eN 0.0 0.5  
Demgraphic models for Yoruba [YRI]
1.	Exponential growth from initial size  to , occurring  generations in the past. 
2.	 = 11156,  = 11156, : 1000: ms 120 1 -s  -r   -eG 0.02241 0 
3.	 = 10659,  = 22600, : 1000: ms 120 1 -s  -r   -eG 0.02345 31.977 
4.	 = 7834,  = 57900, : 4000: ms 120 1 -s  -r   -eG 0.12765 15.668 
5.	 = 10424,  = 938000, : 500: ms 120 1 -s  -r   -eG 0.01199 375.264 
6.	 = 9944,  = 44600, : 1500: ms 120 1 -s  -r   -eG 0.03771 39.776 
7.	 = 11020,  = 23300, : 250: ms 120 1 -s  -r   -eG 0.005672 132.24 
8.	 = 9912,  = 199000, : 1000: ms 120 1 -s  -r   -eG 0.02522 118.944 
9.	 = 10018,  = 1910000, : 750: ms 120 1 -s  -r   -eG 0.01872 280.504
