# Supplementary material for: A Map of Recent Positive Selection in the Human Genome
Source: PLoS Biol. 2006 Mar 7;4(3):e72. doi: 10.1371/journal.pbio.0040072 (PMC1382018; doi:10.1371/journal.pbio.0040072)
Supplement: Supplementary file 33 [file pbio.0040072.st001.doc]

|  | CEU | CEU | YRI | YRI | ASN | ASN |
| --- | --- | --- | --- | --- | --- | --- |
|  | iHS $>$ 2.0 | iHS $\leq$ 2.0 | iHS $>$ 2.0 | iHS $\leq$ 2.0 | iHS $>$ 2.0 | iHS $\leq$ 2.0 |
| Genic | 13619 | 267085 | 14783 | 272331 | 12513 | 249407 |
| Nongenic | 15565 | 354514 | 16195 | 367950 | 14348 | 322565 |
| OR (95% CI) |  | 1.16(1.14–1.19) |  | 1.23(1.20–1.26) |  | 1.13(1.1–1.16) |
|  |  |  |  |  |  |  |
|  | iHS $>$ 2.0 | iHS $\leq$ 2.0 | iHS $>$ 2.0 | iHS $\leq$ 2.0 | iHS $>$ 2.0 | iHS $\leq$ 2.0 |
| Overlapping Genes | 261 | 4474 | 297 | 4473 | 263 | 4240 |
| Single Genes | 13358 | 262611 | 14486 | 267858 | 12250 | 245167 |
| OR (95% CI) |  | 1.15(1.01–1.3) |  | 1.22(1.09–1.38) |  | 1.24(1.09–1.41) |

Table S1. Table comparing the number of SNPs in genic and nongenic regions with high iHS score. The lower table compares the number of SNPs with high iHS that are represented in multiple overlapping open reading frames (ORFs) to those present in single ORF.
