# Supplementary material for: A Map of Recent Positive Selection in the Human Genome
Source: PLoS Biol. 2006 Mar 7;4(3):e72. doi: 10.1371/journal.pbio.0040072 (PMC1382018; doi:10.1371/journal.pbio.0040072)
Supplement: Supplementary file 34 [file pbio.0040072.st002.doc]

| Population | rsnumber | iHS | cM Span | Genes |
| --- | --- | --- | --- | --- |
| East Asian | rs4971100 | -2.842 | 1.39 | GBA, MTX1, ADAM15, LENEP |
|  | rs7441655 | -2.344 | 1.29 | NEK1, SH3MD2,CLCN3 |
|  | rs1442490 | -2.665 | 1.16 | ADH cluster, MTP |
| CEPH | rs6035877 | -2.194 | 1.25 | XRN2, PAX1, NKX2-2 |
|  | rs1426721 | -2.237 | 1.15 | SLC24A5, SLC12A1, MYEF2, FBN1 |
|  | rs6497253 | -3.118 | 1.13 | OCA2, HERC2 |
| Yoruba | rs1160025 | -2.577 | 0.97 | FBXL7 |
|  | rs13332060 | -2.547 | 0.94 | ATPV0C, CCNF, NTN2L, PDPK1 |
|  | rs9536046 | -4.15 | 0.74 | NEK3, ATP7B, CKAP2, LECT1 |

Table S2. Genetic spans of the longest haplotypes in each population. Recorded are the SNPs with derived allele frequency $>50\%$ that have the longest spans between the first point to the left, and first point to the right of the core SNP at which EHH drops below 0.25. The SNPs considered for this analysis were those SNPs with the most negative iHS in each of the top 1% of all surveyed regions.  indicates that the genes listed in the table are among many genes in the region spanned by the haplotypes. The span around the OCA2 gene was manually truncated due to a large gap in the SNP data on one side.
