# Supplementary material for: A Map of Recent Positive Selection in the Human Genome
Source: PLoS Biol. 2006 Mar 7;4(3):e72. doi: 10.1371/journal.pbio.0040072 (PMC1382018; doi:10.1371/journal.pbio.0040072)
Supplement: Supplementary file 35 [file pbio.0040072.st004.doc]

| GO Nesting | GO Category | ASN | CEU | YRI |
| --- | --- | --- | --- | --- |
| 21-1 | Chemosensory Perception | NS | *<*0.0006*A* | *<*0.0004*B* |
| 21-1-1 | Olfaction | NS | *<*0.0006*A* | *<*0.0008*B* |
| 22-2 | Gametogenesis | *<*0.008*C* | NS | NS |
| 22-2-2 | Spermatogenesis and Motility | *<*0.02 | *<*0.03 | NS |
| 22-3 | Fertilization | *<*0.004 | *<*0.003 | NS |
| 1-11 | Other Carbohydrate Metabolism | *<*0.0002*D* | NS | NS |
| 6 | Electron Transport | NS | *<*0.0002*E* | NS |
| 4-13 | Chromatin Packaging/Remodeling | *<*0.0001*F* | *<*0.01*G* | NS |
| 16-1-1 | MHC-I Mediated Immunity | NS | *<*0.0001*H* | *<*0.02 |
| 3-2 | Steroid Metabolism | NS | NS | *<*0.0001*I* |
| 3-5 | Lipid and Fatty Acid Binding | *<*0.001 | NS | NS |
| 4-4-2 | mRNA Transcription Initiation | NS | *<*0.002*J* | NS |
| 5-3 | Protein Modiﬁcation | *<*0.002*K* | NS | NS |
| 7-5 | Vitamin/Cofactor Transport | *<*0.002*L* | NS | NS |
| 9 | Phosphate Metabolism | *<*0.002*M* | *<*0.03*N* | NS |
| 13-4 | Peroxisome Transport | NS | NS | *<*0.002 |

Table S4: Gene clusters that contribute to signiﬁcant GO categories. The signiﬁcance in some categories can be partially attributed to clusters of signiﬁcant genes with similar ontological function. The clusters are in the following locations, with total numbers of genes showing signals in square brackets, and the numbers in each cluster in parentheses: A: [29], 1q44 (11), 6p22.1 (10); B: [34], 11p15.4 (8), 11q11 (6), 11p11.2 (5); C: [28], 6p21-22 (5); D: [9], 4q21-23 (7); E: [27] 1p33-34 (6), 7q21-22 (5); F: [37] 6p21.3 (29); G: [13], 6p21.3 (10); H: [7] 6p21.3 (7); I: [23], 22q12-13 (8); J: [13], 2q33.1 (3); K: [109], 3p21 (7), 11q13 (5), 20q11 (5), 22q11 (4); L: [8], 3q23 (2), 11q13-14 (2); M: [8] 6p21 (3); N: [11] 6p21 (3).
